# Small cities face greater impact from automation

**DOI:** 10.1098/rsif.2017.0946

**Published:** 2018-02-07

**Authors:** Morgan R. Frank, Lijun Sun, Manuel Cebrian, Hyejin Youn, Iyad Rahwan

**Affiliations:** 1 Media Laboratory, Systems, & Society, Massachusetts Institute of Technology, Cambridge, MA, USA; 2 Institute for Data, Systems, & Society, Massachusetts Institute of Technology, Cambridge, MA, USA; 3 Data61 Unit, Commonwealth Scientific and Industrial Research Organization, Melbourne, Victoria, Australia; 4 Kellogg School of Management, Northwestern University, Evanston, IL, USA; 5 Northwestern Institute on Complex Systems, Northwestern University, Evanston, IL 60208, USA; 6 London Mathematical Lab, London WC2N 6DF, UK

**Keywords:** city science, automation, future of work, resilience

## Abstract

The city has proved to be the most successful form of human agglomeration and provides wide employment opportunities for its dwellers. As advances in robotics and artificial intelligence revive concerns about the impact of automation on jobs, a question looms: how will automation affect employment in cities? Here, we provide a comparative picture of the impact of automation across US urban areas. Small cities will undertake greater adjustments, such as worker displacement and job content substitutions. We demonstrate that large cities exhibit increased occupational and skill specialization due to increased abundance of managerial and technical professions. These occupations are not easily automatable, and, thus, reduce the potential impact of automation in large cities. Our results pass several robustness checks including potential errors in the estimation of occupational automation and subsampling of occupations. Our study provides the first empirical law connecting two societal forces: urban agglomeration and automation's impact on employment.

## 1. Introduction

Cities, which accommodate over half of the world's population [1], are modern society's hubs for economic productivity [2–4] and innovation [5–7]. As job migration is the leading factor in urbanization [1,8], policymakers are increasingly concerned about the impact of artificial intelligence and automation on employment in cities [9–11]. While researchers have investigated automation in national economies and individual employment, it remains unclear *a priori* how cities naturally respond to this threat. In a world struggling between localism and globalism, a question emerges: *how will different cities cope with automation?* Answering this question has implications on everything from urban migration to investment, and from social welfare policy to educational initiatives.

To construct a comparative picture of automation in cities, our first challenge is to get reliable estimates of how automation impacts workers. Existing estimates are wide ranging. Frey & Osborne [12] estimate that 47% of US employment is at ‘high risk of computerization’ in the foreseeable future, while an alternative OECD study concludes a more modest 9% of employment is at risk [13]. Note that these results do not tell us about the impact of automation in cities as they are presented at a national level. Differences in these predictions arise from discrepancies over two main skill dynamics: the substitution of routine skills, and complementarity of non-routine and communication skills [14–16]. Additionally, technology-driven efficiency may redefine the skill requirements of occupations and actually increase employment in low-skilled jobs [17,18].

Nevertheless, even if we take current estimates of the *absolute* risk of computerization of jobs with skepticism, these estimates can provide useful guidance about *relative* risk to different cities that is robust to errors in the estimates provided by Frey & Osborne [12] and Arntz *et al*. [13]. We can interpret the ‘risk of computerization’ estimates as an educated guess about which occupations will experience greater adjustment due to machine substitution of a large portion of their content. These adjustments represent a significant cost to an urban system from both technological unemployment and expensive worker retraining programmes.

*A priori*, it is not obvious whether large cities will experience more or less impact from automation. On one hand, an influx of occupational diversity explains the wealth creation, innovation and success of cities [19–22]. On the other hand, cities connect people with greater efficiency [22,23]. This enables a greater division of labour that increases overall productivity [24–26] through occupational specialization. However, the division of labour may facilitate automation as it identifies routine tasks and encourages worker modularity. If these modular jobs are at greater risk of computerization, then more workers may be impacted by automation in large cities. These observations pose a puzzle: *are the forces of diversity, specialization and the division of labour shaping a city's ability to accommodate automation?*

Here, we undertake a comparative examination of cities while measuring the relative impact of automation on employment. We also contextualize these measurements through a detailed analysis of the skill composition of different cities. Note that *impact* includes unemployment, but may also manifest itself through the changing skill demands of occupations as automation diminishes the need for individual types of skills [17,18]. In the light of imminent automation technology, we highlight a complicated relationship between labour diversity and specialization in cities, and discover that small cities are susceptible to the negative impact of automation.

## 2. Material and methods

### 2.1. Datasets

The US Bureau of Labor Statistics (BLS) data identify the employment distribution of about 700 different occupations across each of 380 US metropolitan statistical areas (MSAs) and combined statistical areas (CSAs) in 2014. (We refer to both CSAs and MSAs as ‘cities’.) We consider MSAs in isolation only when they are not part of a CSA. CSAs have arisen as the best approximation for determining cities [5,6,27–31]. The resulting list of occupations considered in this study represents 99.99% of national employment according to the occupational employment statistics data produced annually by BLS. From these employment distributions, we calculate the probability of a worker in city *m* having job *j* according to2.1
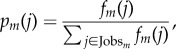
where Jobs_*m*_ denotes the set of job types in city *m* according to BLS data, and *f*_*m*_(*j*) denotes the number of workers in city *m* with job *j*.

For each occupation, the BLS O*NET dataset details the importance of 230 different workplace skills, such as Manual Dexterity, Finger Dexterity, Complex Problem Solving, Time Management and Negotiation. BLS obtains this information through several separate surveys which group the raw O*NET skills into the following categories: Abilities, Education/Training/Experience, Interests Knowledge, Skills, Work Activities and Work Context. We normalize the raw survey responses to obtain a value between 0 (irrelevant to the occupation) and 1 (essential to the occupation) indicating the absolute importance of that skill to that occupation. We refer to these values of skill importance as *raw skill values*.

### 2.2. Measures for specialization and diversity

We assess the specialization or diversity of the employment distribution in city *m* by calculating the normalized Shannon entropy. Shannon entropy [32], an information-theoretic measure for the expected information in a distribution, can be normalized according to2.2
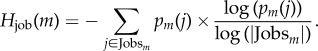
This quantity measures the predictability of an employment distribution given the set of unique occupations in a city. The measure is maximized when the distribution is least predictable (i.e. the distribution is uniform). Therefore, the denominator of log(|Jobs_*m*_|) normalizes the entropy score so that we can compare the distributions of jobs in cities with different sets of job categories (see electronic supplementary material, S2.1 for further discussion). The values for normalized Shannon entropy lie between 0 (specialization) and 1 (diversity). Normalized Shannon entropy has been used in a variety of fields, including virology [33], climatology [34] and city science [35].

For a given occupation, we normalize each raw skill value by the sum of the values to obtain the relative importance of each skill to that occupation (denoted *p*_*j*_(*s*)). Similarly to above, we measure the normalized Shannon entropy of the relative skill distribution of job *j* according to2.3
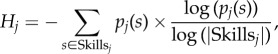
where Skills_*j*_ denotes the set of O*NET skills with non-zero importance to job *j*. We employ normalized Shannon entropy here to facilitate a fair comparison of relative skill distributions between jobs which may have received the same raw O*NET value for a given skill, but have different numbers of non-zero raw O*NET skills.

We obtain a distribution of relative skill importance for a city according to2.4
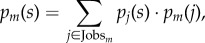
where *p*_*m*_(*s*) is the relative importance of skill *s* in city *m*. Again, we use normalized Shannon entropy to assess the skill specialization in a city according to2.5
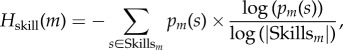
where Skills_*m*_ represents the set of O*NET skills with non-zero importance in city *m*.

These aggregate skill distributions for a city may obfuscate the specialization of skills through the relative abundance of jobs in that city. For example, the city-level aggregation of skills may appear diverse, while the jobs within the city are actually specialized. The Theil entropy [36] of a city is a multi level information-theoretic measure defined by2.6
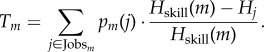
*T*(*m*) = 1 indicates that each job specializes in exactly one skill, and *T*(*m*) = 0 indicates that the specialization of skills among jobs is equal to the specialization of skills on the city-level aggregation. We do not observe any jobs relying on exactly one skill, and so we expect the Theil entropy of any given city to be well below 1. We present 1 − *T*_*m*_ throughout the study for easy comparison to Shannon entropy. Note that both normalized Shannon entropy and Theil entropy are unit-less measures due to the normalizations employed; we therefore do not focus on their range of values across cities, but instead we focus on the relationship between labour specialization/diversity and other urban indicators.

## 3. Results

### 3.1. The expected job impact of automation in cities

**Figure 1.**
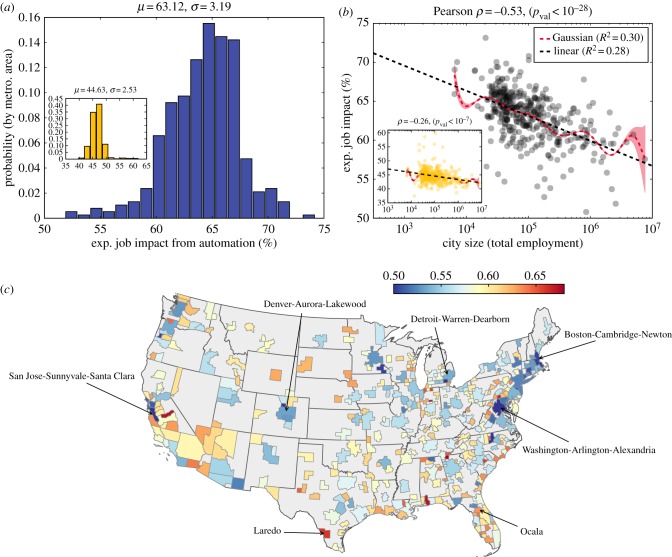
The impact of automation in US cities. (*a*) The distribution of expected job impact (*E*_*m*_) from automation across US cities using estimates from Frey & Osborne [12]. (Inset) The distribution using alternative estimates [13]. (*b*) Expected job impact decreases logarithmically with city size using estimates from Frey & Osborne [12]. We provide the line of best fit (slope = − 3.215) with Pearson correlation to demonstrate significance (title). We also provide a Gaussian kernel regression model with its associated 95% confidence interval. (Inset) Decreased expected job impact with increased city size is again observed using alternative estimates [13] (best fit line has slope −1.24, Pearson *ρ* = − 0.26, *p*_val_ < 10^−7^). (*c*) A map of US metropolitan statistical areas coloured according to expected job impact from automation.

We estimate automation's expected impact on jobs in cities according to3.1
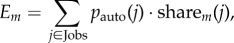
where Jobs denotes the set of occupations, share_*m*_(*j*) denotes the employment share (as a percentage) in city *m* with occupation *j* according to the US BLS and *p*_auto_(*j*) denotes the probability of computerization for occupation *j* as estimated by [12] (see electronic supplementary material, S3 for more details). We can interpret *E*_*m*_ as the expected percentage of total employment in city *m* subject to computerization. Each city should expect between one-half and three-quarters of their current employment to be affected in the foreseeable future due to improvements in automation (see figure 1*a*; also note that this estimate differs from that in [12], which focused on national statistics). While this calculation omits potential job creation or job redefinition which typically accompany innovation [37,38], it highlights the differential impact of automation across cities and smooths potential noise in the predicted automation of individual jobs. Expected job impact may represent employment loss or changes in the type of work performed by those workers (e.g. see [11,17,18]), which, in turn, may not produce changes in net employment.

What differentiates cities' resilience to automation? Figure 1*b* demonstrates that expected job impact decreases according to *E*_*m*_∝ − 3.2 × log_10_(city size), which suggests that larger cities are more resilient to the negative effects of automation. This relationship is significant with a Pearson correlation *ρ* = − 0.53 (*p*_val_ < 10^−28^), and shows that labourers in smaller cities are susceptible to the impact of automated methods (*R*^2^ = 0.28). We confirm our finding using separate conservative skill-based estimates of the automatability of jobs [13] (Pearson *ρ* = − 0.26 (*p*_val_ < 10^−7^) and *E*_*m*_∝ − 1.24 × log_10_(city size). (See figure 1*b* inset; electronic supplementary material, S3.2.) Despite the conservative nature of these alternative probabilities, we again observe increased resilience with city size. Furthermore, we demonstrate in electronic supplementary material, S3.1 that the observed negative trend relating city size to expected job impact from automation is robust to errors in the probabilities of computerization (i.e. *p*_auto_) produced by Frey & Osborne [12] and robust to random removal of occupations from the analysis.

### 3.2. Labour specialization in large cities

**Figure 2.**
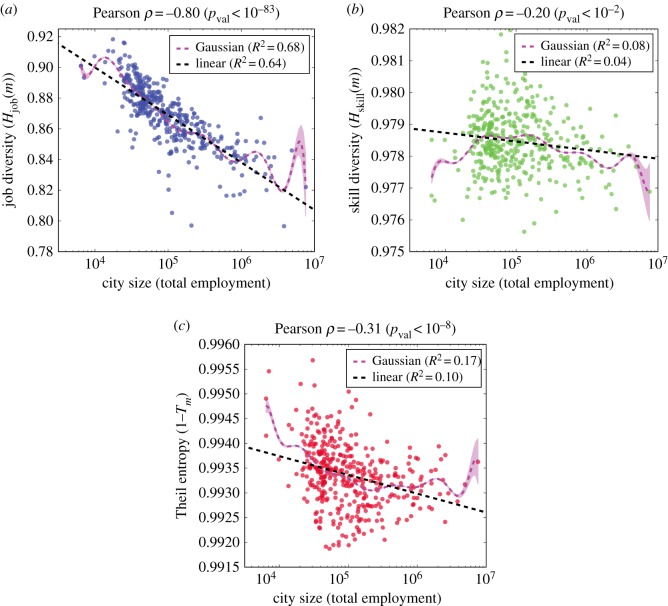
Large cities reveal increased occupational specialization through both job and skill distributions. (*a*) Shannon entropy of job distributions, *H*_job_(*m*), decreases with city size. (*b*) Shannon entropy of the O*NET skill distributions, *H*_skill_(*m*), decreases with city size. (*c*) Theil entropy, *T*_*m*_, reveals the proportion of specialized jobs increases with city size. For plots (*a*), (*b*) and (*c*), we provide the line of best fit for reference, and we provide a Gaussian kernel regression model with its associated 95% confidence interval.

We explore the mechanisms underpinning resilience to automation by examining the most distinctive characteristics of urban economies: diversification and specialization. In particular, how does labour diversity, or specialization, mediate the relationship between city size and the expected job impact from automation? As automation typically targets workplace skills [13], we consider the O*NET skill dataset, which relates occupations to their constituent workplace tasks and skills, in addition to employment data. For large cities, specialization (i.e. decreased Shannon entropy) appears in the employment distributions across occupations (figure 2*a*) and, separately, in the aggregate distributions of skills (figure 2*b*). Additionally, we use Theil entropy to measure the proportion of specialized jobs (in terms of skills) in comparison to the skill specialization of the city on the whole. Figure 2*c* demonstrates an increasing proportion of specialized jobs in large cities (i.e. 1 − *T*_*m*_ decreases). See Material and methods for calculations of entropy measures.

**Figure 3.**
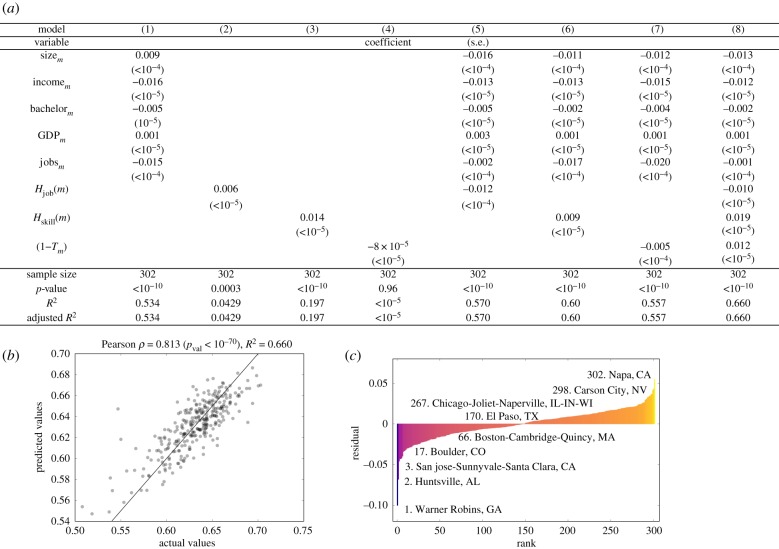
Labour specialization can model expected job impact (*E*_*m*_) in cities. (*a*) A multiple linear regression analysis for predicting *E*_*m*_ that considers generic urban indicators including log_10_ city total employment (size_*m*_), median annual household income (income_*m*_), percentage of population with a bachelor's degree (bachelor_*m*_), log_10_ GDP *per capita* (GDP_*m*_) and the number of unique occupations (jobs_*m*_). All variables have been standardized. (*b*) The actual *E*_*m*_ values for each city plotted against the predicted values using model 8 from (*a*), which captures 66% of the variance in expected job impact from automation across US cities (see electronic supplementary material, S4 for additional analysis). (*c*) The distribution of residuals between the actual and predicted values from model 8, and the rank of some example cities.

In figure 3, we examine eight regression models attempting to model the differential impact of automation across cities. In model 1, we first examine a baseline model using only generic urban variables, including city size (denoted by size_*m*_), median household income (income_*m*_), the per cent of population with a bachelor's degree (bachelor_*m*_), *per capita* GDP (GDP_*m*_) and the number of unique job titles (jobs_*m*_). This generic model captures 53% of the variance in expected impact from automation across US cities. Models 2, 3 and 4 use the information-theoretic measures in three separate linear regression models to reveal that skill specialization (i.e. *H*_skill_(*m*)) is the most predictive of expected job impact in cities (*R*^2^ = 0.20) in the absence of other urban variables.

In models 5, 6 and 7, we demonstrate that the inclusion of each of the specialization measures produces models accounting for additional variance in expected impact over the use of generic variables alone. In particular, the inclusion of skill specialization (i.e. *H*_skill_(*m*)) yields a model accounting for 60% of the variance in job impact (see model 6). Finally, we include all variables in a single model (see model 8) which produces the most predictive model accounting for 66% of the variance across cities (figure 3*a*,*b*). We confirm the stability of our regression results by alternatively training the regression model on half of the cities and measuring the performance of the regression on the remaining cities as validation (see electronic supplementary material, S4).

Each model that we tested yielded statistically significant coefficient estimates (note that variables were standardized before regression) and the inclusion of our labour specialization metrics yielded models with improved predictive power. Furthermore, we performed a formal mediation analysis that is presented in electronic supplementary material, §3.5. However, these observations should not be taken as conclusive evidence that labour specialization, or diversity, is causally related to the expected impact from automation in a city. This is due, in part, to the colinearity between variables used in model 8. For example, the city size coefficient (size_*m*_) changes sign across the models in our analysis because of the strong relationship between city size and labour specialization, which we demonstrate in figure 2.

The residuals between the actual and modelled values according to model 8 highlight notably resilient cities (given the model), such as Boulder, CO, and Warner Robins, GA, and notably susceptible cities, such as Napa, CA, and Carson City, NV (figure 3*c*). Examining these cities more closely may allow urban policy experts with a nuanced understanding of the policies in these cities to more easily identify causal mechanisms. The predictive power of this model and its reliance on workplace skills justifies our inclusion of skills data in addition to occupation data, and motivates us to characterize urban resilience to automation from the skills in cities.

### 3.3. How occupations and workplace skills change with city size

How do different types of occupations change with city size [39], and how do these changes contribute to the differential impact of automation across cities? While it is tempting to look only for the largest changes in employment share, more subtle differences for very automatable, or very not automatable, occupations can also produce big changes in expected job impact. We capture this confounding effect by decomposing the difference in expected job impact of cities *m* and *n* according to3.2
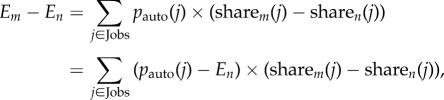
where we have profited from 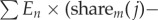 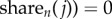. We consider the percentage of the difference explained by occupation *j* according to3.3
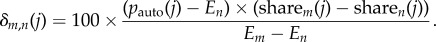


Occupation *j* can increase or decrease the overall difference in expected job impact depending on the sign of the corresponding term in equation (3.2), or, equivalently, the sign of *δ*_*m*,*n*_(*j*). In turn, this sign depends on the relative automatability of the occupation and the relative employment share. More details for this calculation and an example analysis comparing individual cities are provided in electronic supplementary material, S3.4.

**Figure 4.**
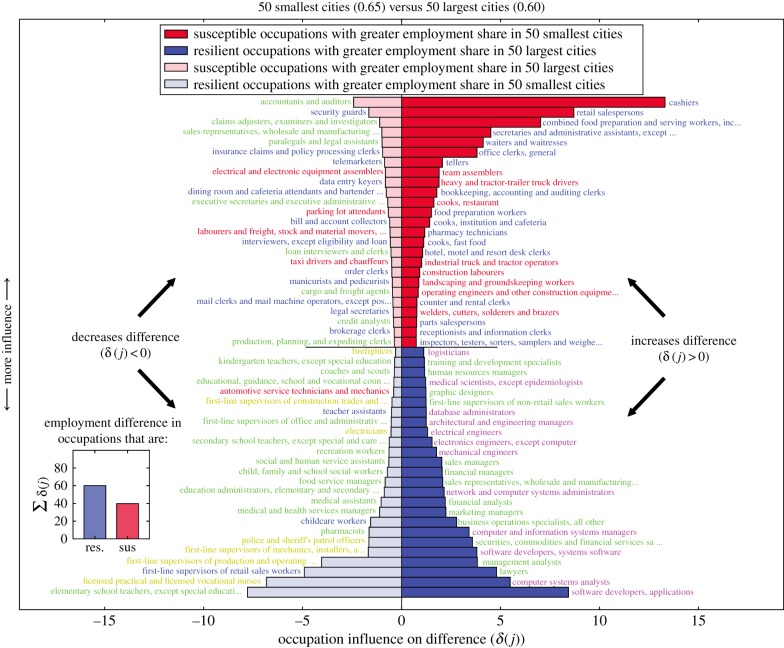
An occupation shift explaining the difference in expected job impact for the 50 largest cities (impact: 0.60) compared to the 50 smallest cities (impact: 0.65) using equation (3.2). Each horizontal bar represents *δ*_(small cities, large cities)_(*j*). The occupation title is provided next to the corresponding bar and coloured according to its job cluster. Red bars represent occupations with higher risk of computerization compared to the expected job impact in large cities. Blue bars represent occupations with lower risk of computerization compared to the expected job impact in large cities. Dark colours represent occupations that increase the difference, while pale colours represent occupations that decrease the difference in expected job impact. Bars in each of the quadrants are vertically ordered according to |*δ*_(small cities, large cities)_(*j*)|. The inset in the bottom left of the plot summarizes the overall influence of resilient occupations compared to occupations that are at risk of computerization.

In figure 4, we employ an ‘occupation shift’ to visualize the contributions of each occupation to the difference in expected job impact in large and small cities. After adding the employment distributions for the 50 largest cities and 50 smallest cities together, respectively, we calculate *δ*(*j*) for each occupation. Each occupation is assigned a quadrant and colour based on the sign of *δ*(*j*) and the relative automatability of occupation *j*. This visualization identifies both occupations that increase the differential impact (i.e. occupations on the right) and occupations that decrease the differential impact (i.e. occupations on the left). For example, increased employment for Cashiers, which is relatively susceptible to automation, in small cities contributes the most to the overall difference in expected job impact. Likewise, differences in employment for Software Developers, a relatively resilient occupation, also increases the overall difference. On the other hand, increased employment for Elementary School Teachers, which is another relatively resilient occupation, in small cities decreases the difference. On aggregate, differences in employment for occupations that are relatively resilient to automation contribute the most to the differential impact of automation in large and small cities (see figure 4 inset).

**Figure 5.**
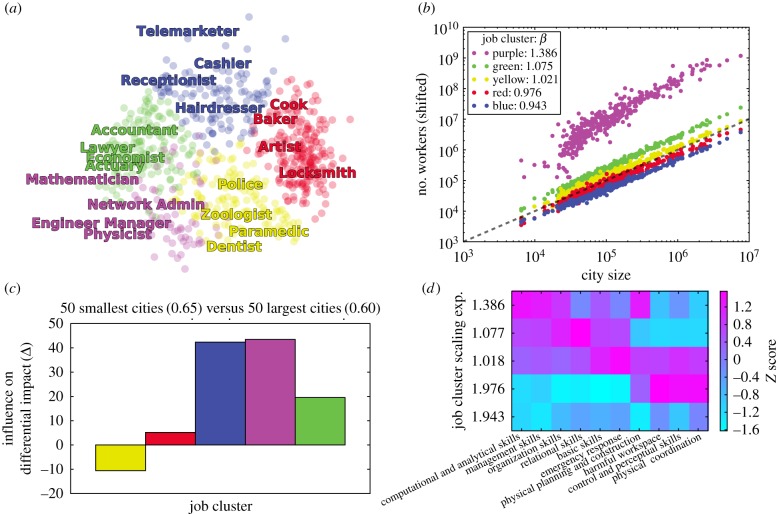
Technical occupations grow superlinearly with city size. (*a*) We project jobs onto a two-dimensional plane using principal component analysis. A few representative jobs from each cluster are highlighted (colour). (*b*) We plot the employment (*y*-axis) in a given job cluster (colour) versus the total employment in a city (*x*-axis), and vertically shift points according to the linear fit in log scale. The black dashed line has a slope of 1 for reference. (*c*) The influence of each job cluster on the difference in expected job impact of the 50 largest cities (*E*_large cities_ = 0.60) compared to the 50 smallest cities (*E*_small cities_ = 0.65) according to equation (3.4). (*d*) After summing the importance of each skill type to each job cluster, we calculate *z* scores for a skill type according to the distribution of importance across job clusters.

To explore the role of resilient occupations further, we focus on how employment for different occupation types changes with city size. We use the K-means clustering algorithm (i.e. occupations are instances and raw O*NET skill importance are features) to identify five clusters of jobs according to skill similarity (see figure 4 occupation labels and figure 5*a*; the complete list of occupations is provided in electronic supplementary material, S6.3) and examine the scaling relationship between job clusters and city size according to (number of workers)∝(city size)^*β*^ in figure 5*b*. Note that the exponent, *β*, entirely describes the growth rate of these job clusters relative to city size. The job cluster comprising highly specialized jobs, such as Mathematician and Chemist, exhibits a notably superlinear scaling relationship with city size (*β* = 1.39). This scaling exponent is similar to the scaling relationship observed for *Private R&D employment* (*β* = 1.34) found in [6] and is in good agreement with similar studies on job growth [17]. Furthermore, our finding of one job cluster exhibiting notably larger scaling than the other job clusters is stable to sub-sampling occupations at various rates (see electronic supplementary material, S6.3.2). Managerial jobs also grow superlinearly, but to a weaker extent (*β* = 1.08). The job cluster exhibiting the slowest growth (*β* = 0.94) comprises entertainment and service jobs. We check the robustness of these scaling relationships using methods from [40] (see electronic supplementary material, S6.3.3).

In figure 5*c*, we quantify each job cluster's contribution to the differential impact of automation across large and small cities according to3.4
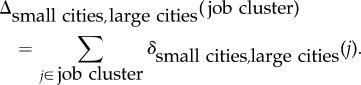
The low automatability and high difference in employment of highly specialized job cluster (represented by purple) in large and small cities indeed explains a significant amount of the difference in expected job impact. However, we also find that the more susceptible occupations represented by the blue job cluster in figure 5 accounts for a similar proportion of the difference. Interestingly, the differences in occupations from the yellow job cluster serve to decrease the differential impact of automation between large and small cities. This conclusion is supported by the analysis of individual occupations presented in figure 4.

We confirm that the fastest growing job cluster is indeed comprised of ‘technical’ jobs based on their constituent workplace skills. We employ K-means clustering (i.e. O*NET skills are instances and the correlation of raw O*NET importance of skills across occupations are features) to simplify the complete space of O*NET skills to 10 skill types based on the co-occurrence of skills across jobs (see electronic supplementary material, S6.5 for complete description of skill clusters). These simplified skill types allow us to intuitively explore which skills indicate specialization or indicate resilience in cities. Computational/Analytical skills and Management skills are more likely in faster growing (i.e. superlinear) jobs, while physical skills, such as Physical Coordination and Control/Perceptual skills, indicate notably slower job growth with city size (figure 5*d*). We confirm our findings using alternative definitions for aggregate workplace tasks and skills (see electronic supplementary material, S5).

**Figure 6.**
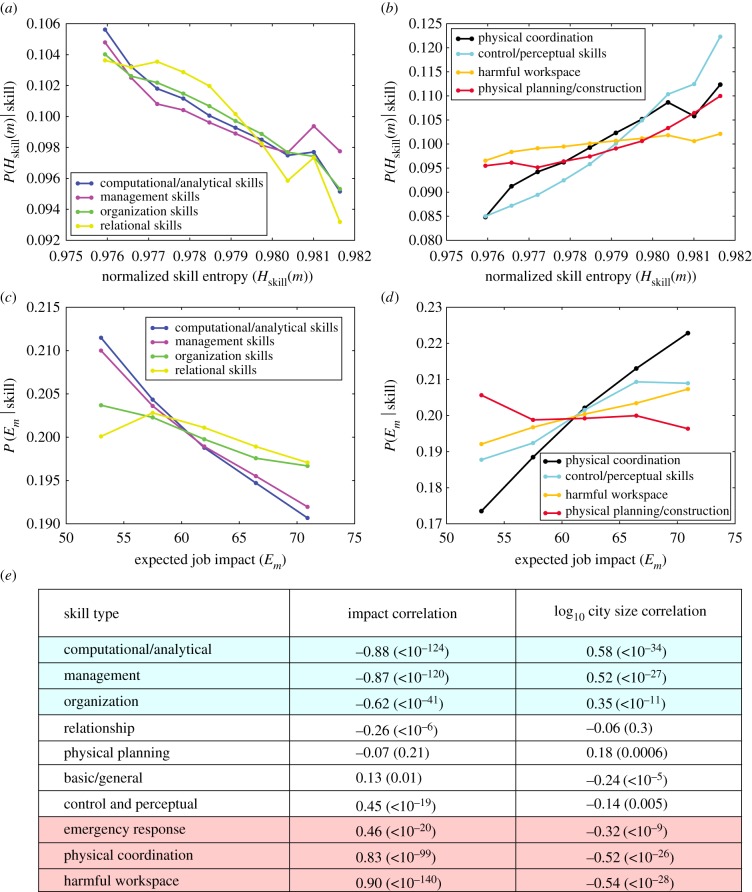
Workplace skills explain occupational specialization and job impact in cities. (*a*,*b*) Skill types in (*a*) indicate specialized cities, while skill types in (*b*) indicate occupational diversity. (*c*,*d*) Skill types in (*c*) indicate resilient cities, while skill types in (*d*) indicate increased job impact from automation. (*e*) The Pearson correlation of skill type abundance to the expected job impact and to log_10_ city size with *p*-values in parentheses. See electronic supplementary material, S6.4 for a similar table for raw O*NET skills.

The skills that are relied on by fast-growing technical jobs suggest mechanisms for resilience and growth in cities. Existing work [41] identifies that individual workers can gain skills to compete with automation, gain skills to complement automation, or seek industries removed from the impacts of automation. Similar to individual workers, the division of labour in large cities allows them to specialize in skills removed from the threat of automation. Computational/Analytical, Managerial, Organization, and Relational skills are more likely to be present in specialized and resilient cities (figure 6*a*,*c*), while Physical Coordination and Control/Perceptual skills indicate both decreased specialization and decreased resilience in cities (figure 6*b*,*d*). We confirm our results using alternative groups of workplace tasks [42] provided by O*NET (see electronic supplementary material, S5.1) and again by examining the routineness of workplace tasks [14] (see electronic supplementary material, S5.2). Figure 6*e* reflects the same conclusion by comparing the relationship of each skill type to city size (right column) and expected job impact (middle column; see electronic supplementary material, S6.4 for comparison with raw O*NET skills). Effectively, large cities employ workers whose skills better prepare them to interface with automation technology, while small cities rely more prominently on physical workers, who are more susceptible to automation.

### 3.4. Limitations

Many of the limitations inherent to occupation-level predictions [12,13] apply to our study as well. Specifically, our measure for the expected impact of automation in cities may represent technological unemployment, but also represents the skill recomposition of occupations in response to new technology. This means the expected impact of automation in cities may not relate to changes in net employment in cities. The actual effects of automation on net employment levels depend on several systemic variables including the availability of cheap labour [43,44], future regulations around technology (e.g. taxing the use of robotics) and market demand with increased worker efficiency [17,18].

Nevertheless, we expect the impact we are measuring to correspond to costly real-world changes in labour that high-impact cities must overcome. For example, cities with high expected impact from automation will need to invest in worker retraining programmes. These programmes minimize technological unemployment by adapting the existing skills of workers to match the evolving skill demands with changing technology, but these programmes are costly. Urban policymakers may also mitigate net employment loss by investing in new industries, but successful investment of this kind requires costly research and capital to attract those companies to a city.

## 4. Discussion

Cities are modern society's hubs for economic productivity and innovation. However, the impact of automation on employment in cities threatens to alter urbanization, which is largely driven by employment opportunity. Fortunately, urbanization itself appears to contain a mitigating solution. It is difficult to concretely identify causal mechanisms at the scale of this investigation, but, despite this difficulty, we highlight evidence for the division of labour in large cities and show its importance as a piece of the automation and urbanization puzzle.

In particular, large cities have more unique occupations and industries [7], but distribute employment less uniformly across those occupations. This juxtaposition of both diversity and specialization in large cities is reconcilable through the division of labour theory [24]. Under the division of labour argument, large firms have better ability to support specialized workers along with the management required to coordinate them [45]. To this end, we find that the average number of workers per firm increases logarithmically with city size (see electronic supplementary material, figure S1A). At the same time, workers possessing specialized skills seek specific employment opportunities which maximize their financial return [46,47]. The demand for specific specialized jobs increases occupational specialization while also increasing the number of unique job types and industries in a city [8].

What do large cities specialize in and why? The division of labour encourages worker modularity, which has the potential to impact whole groups of workers who are competing with automation technology. Therefore, specialization alone is not enough to explain the resilience to automation impact that we observe across cities. For example, Detroit, which is famous for its specialization in automotive manufacturing, has experienced economic downturn [48], while the San Francisco Bay area, the epicentre of the information technology industry, continues to flourish despite the dot-com bubble (perhaps due to its support of a ‘creative class’ of workers [49]). Our analysis highlights specific occupations, such as Mathematician and Chemist, as well as specific types of skills, such as Computational/Analytical skill, that explain the increased resilience of large cities. These occupations and skills may inform policymakers in small cities as they identify new industries and design worker retraining programmes to mitigate the negative effects of automation on employment.

By quantifying relative *impact*, we provide an upper bound for *technological unemployment* in cities. Changing labour demands produce systemic effects, which make it difficult to precisely predict employment loss [15]. For example, the introduction of automated teller machines (ATMs) suggested a likely decrease in human bank teller employment. However, contrary to this prediction, ATM technology cut the cost to banks for opening and operating new branches, and, as a result, national bank teller employment *increased* [17,18]. However, these bank tellers performed different tasks, such as relationship management and investment advice, which required very different skills. Hence, by *impact*, we refer to the *magnitude* of the skill substitution shocks that cities must respond to.

The actual technological unemployment in a city will be shaped both by free market dynamics (e.g. shifts in supply and demand curves) and by economic and educational policy (e.g. worker retraining, or skilled migration). Nevertheless, we observe a strong trend relating city size to automation impact that is robust to errors in the automatability of individual occupations and occupational subsampling. For example, the estimates of occupational automation, which we employ in our analysis, would need to be severely flawed (errors over 50%) for the negative dependency on city size to disappear. Recognizing that small cities will experience larger adjustments to automation calls on policymakers to pay special attention to the pronounced risks we have identified.

Despite being seemingly unrelated societal forces, we uncover a positive interplay between urbanization and automation. Larger cities not only tend to be more innovative [5,6], but also harbour the workers who are prepared to both use and improve cutting-edge technology. In turn, these workers are more specialized in their workplace skills and less likely to be replaced by automated methods in the foreseeable future. These findings open the door for more controlled investigations with input from policymakers.

## Supplementary Material

Small cities face greater impact from automation: Supplementary Material

## Data accessibility

All data needed to evaluate the conclusions in this paper are present in the paper and/or the electronic supplementary materials. Additional data related to this paper may be requested from the authors.
