# Supplementary material for: Small cities face greater impact from automation
Source: J R Soc Interface. 2018 Feb 7;15(139):20170946. doi: 10.1098/rsif.2017.0946 (PMC5832739; doi:10.1098/rsif.2017.0946)
Supplement: Small cities face greater impact from automation: Supplementary Material [file rsif20170946supp1.pdf]

Small cities face greater impact from automation: Supplementary Material

Morgan R. Frank[1], Lijun Sun[2], Manuel Cebrian[3], HyeJin Youn[4], Iyad Rahwan[5]

[1]mrfrank@mit.edu, [2]sunlijun@mit.edu, [3]manuel.cebrian@nicta.com.au, [4]youn@maths.ox.ac.uk, [5]irahwan@mit.edu

January 10, 2018

Contents

1 Firm Size Increases with City Size **1**

2 Measuring Labor Specialization **2**
- 2.1 Normalized Shannon Entropy . . . 2
- 2.2 The Labor Specialization of Individual Jobs . . . 3
- 2.3 Characterizing Specialization through O*NET Skills . . . 3

3 Estimating the Affects of Automation **4**
- 3.1 Estimating Automation Impact using Frey/Osborne Data . . . 4
- 3.2 Estimating Automation Impact using OECD Data . . . 6
- 3.3 Expected Job Impact & Labor Specialization in Cities . . . 6
- 3.4 Explaining Differences in Expected Job Impact . . . 8
- 3.5 Labor Specialization as a Mediator for City Size and Automation Impact . . . 11

4 Robustness Check of the Linear Regression Model for E_m **12**

5 Simplifying Jobs & Skills **13**
- 5.1 O*NET Task Groups . . . 14
- 5.2 The Routineness of Tasks . . . 14

6 Data Tables **17**
- 6.1 Cities Ordered by Expected Job Impact from Automation . . . 17
- 6.2 Relating City Trends to BLS Jobs . . . 24
- 6.3 Job Groups . . . 35
  - 6.3.1 Alternative Job Groups using K-means . . . 38
  - 6.3.2 Stability Testing for Job Groups . . . 40
  - 6.3.3 Checking the Statistical Robustness of Job Group Scaling . . . 42
- 6.4 Relating City Trends to O*NET Skill . . . 44
- 6.5 Skill Types . . . 49

1 Firm Size Increases with City Size

The U.S. Bureau of Labor Statistics (BLS) uses the annual tax filings of companies to produce a yearly census of those companies. Unfortunately, the data available to the public doesn't include the specific distribution of BLS jobs

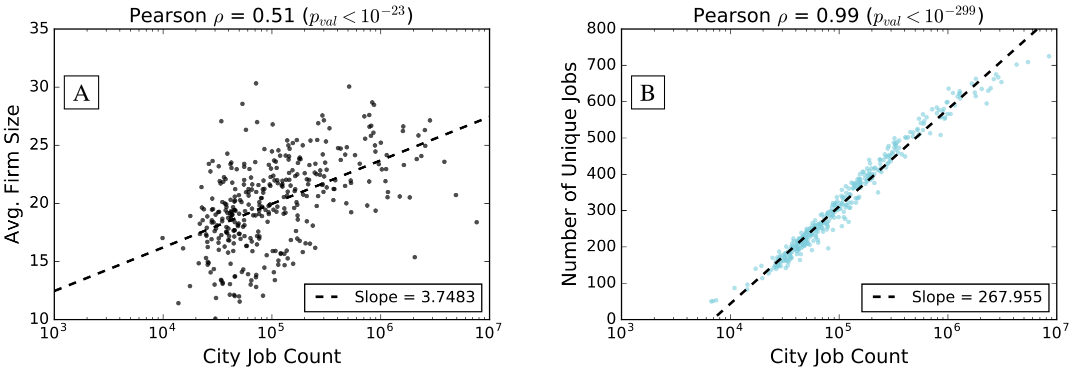

Figure 1: **(A)** The average number of workers per firm grows logarithmically with city size. **(B)** Consistent with [1], we find the number of unique jobs grows logarithmically with city size.

comprising each firm. Previous work has shown that firm sizes nation wide follow a Zipf distribution [2] indicating that a majority of firms are small, but surprisingly large ones also exist infrequently. Figure 1 shows that the average number of workers per firm increases logarithmically with city size. Larger firms have more capital with which to hire specialized workers along with organization/managerial staff to coordinate those workers. According to our theory, there exists a positive feedback loop where large firms provide demand for specialized workers and cities provide a richer market of skilled workers to meet that demand.

2 Measuring Labor Specialization

2.1 Normalized Shannon Entropy

We employ normalized Shannon entropy, as opposed to the standard Shannon entropy definition, to control for size effects on the distributions in cities. For example, it has been shown that the number of different occupations grows with city size (see [1], and SM Fig. 1B), but this result may be due to randomness as more people (e.g. sampled from a long-tailed distribution) are added to a city, and does not account for how workers are distributed amongst these occupations. Large cities may have only a few workers of otherwise absent occupations in small cities, but, perhaps, this distinction does not mean much qualitatively. This motivates us to consider the distribution of workers amongst different occupations, rather than only considering the number of occupations, and to apply relevant information theoretic methods to measure the diversity/specialization of these distributions. We normalize the standard Shannon entropy calculation to control for the number of different occupations in a city, or, equivalently, we normalize Shannon entropy by the maximum possible Shannon entropy given the number of different occupations in the city (i.e. given a number of occupations, Shannon entropy is maximized for the uniform distribution).

This normalization is a standard practice for comparing the diversity or information of systems of different sizes. For a summary of normalized entropy, see [3]. In particular, normalized Shannon entropy has been used in a variety of fields, including virology [4], climatology [5], and city science [6]. To understand this normalization, consider that a sufficient number of roles of a fair 6-sided dice and, separately, of a fair 20-sided dice should each produce uniform distributions with maximized Shannon entropy. However, the Shannon entropy of the distribution for the 6-sided dice is $-\sum \frac{1}{6} \cdot \log(\frac{1}{6}) = 1.79$ and the entropy of the distribution for the 20-sided dice is $-\sum \frac{1}{20} \cdot \log(\frac{1}{20}) = 3.00$; specifically, they are not equivalent despite both being discrete uniform distributions because the distributions have a different number of bins. This is analogous to cities having a different number of unique occupations due, potentially, to randomness that occurs with increased city size. We control for this effect by normalizing Shannon entropy according to the maximum possible Shannon entropy given the number of bins in the discrete distribution. Specifically, given a discrete system with N bins (i.e. N-sided dice, or a city with N unique occupations), Shannon entropy is maximized

when the distribution is uniform, and the maximum value is given by

$$-\sum_{i=1}^{N}\frac{1}{N}\cdot\log(\frac{1}{N}) = -N\cdot\frac{1}{N}\cdot\log(1/N) = -\log(1/N) = \log(N). \quad (1)$$

Therefore, to normalize Shannon entropy according to the maximum possible Shannon entropy, we divide the standard Shannon entropy calculation by $\log(N)$ to obtain

$$-\sum_{i=1}^{N} p_i \cdot \frac{\log(p_i)}{\log(N)},$$

where p_i is the probability of bin i. This normalized Shannon entropy produces a value of 1 for discrete uniform distributions regardless of the number of bins (i.e. regardless of N). In particular, this normalization allows us to control for the number of unique occupations across cities of different sizes to determine the uniformity of job and skill distributions in cities.

2.2 The Labor Specialization of Individual Jobs

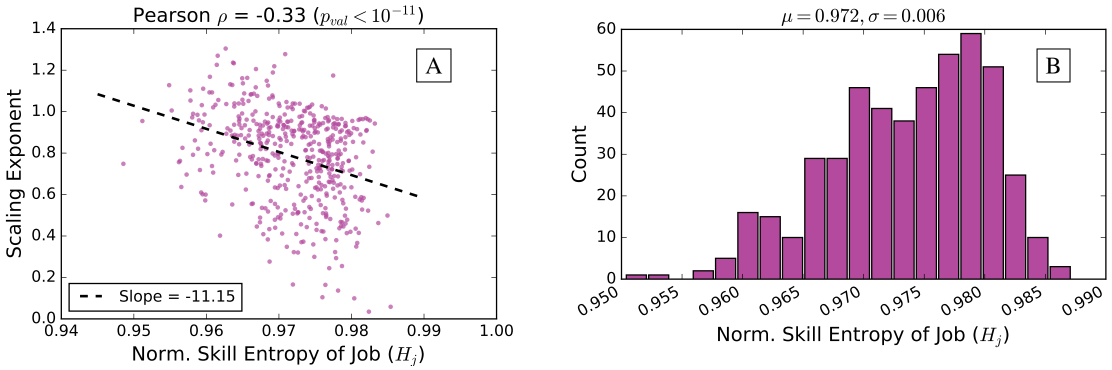

Figure 2: Characterizing the skill specialization of individual jobs. **(A)** Skill specialization indicates larger scaling exponents with city size for individual jobs. **(B)** The distribution of skill specialization across BLS jobs.

We present BLS jobs ordered by decreasing skill specialization in Table 6.2. We also provide the scaling exponent of each BLS job, along with the Pearson correlation of the relative abundance of each job to the expected job impact from automation (discussed below) across cities. Figure 2A shows that specialized jobs tend to have larger scaling exponents. Figure 2B shows the distribution of job specialization.

2.3 Characterizing Specialization through O*NET Skills

We want to understand how each O*NET skill contributes to the relationships we observe. We present our findings in Table 6.4. First, we compare the raw importance of a skill in each city by summing the raw importance of the skill across each job. We then measure the Pearson correlation of the sum of a given skill compared to the expected job impact of each city (denoted ρ_E, second column of table), the skill entropy each city (denoted ρ_H, third column of table), and the size of each city (denoted $\rho_{\log(N)}$, right-most column of table). The skills in the Table 6.4 are ordered according to their correlation with expected job impact in cities. For each column, the p-value for the correlation is presented in parentheses.

Figure 3 allows us to understand how related each correlation is by taking the Pearson correlation of each ρ we described above. Figure 3A demonstrates that skills which indicate lower expected job impact in cities also indicate greater skills specializations in cities. Figure 3B demonstrates that skills which indicate lower expected job impact in cities also indicate larger cities. Interestingly, Figure 3C demonstrates that skills which indicate skill specialization in

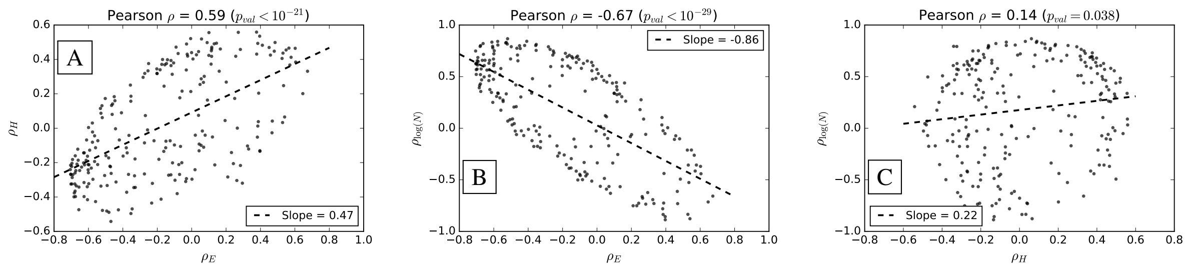

Figure 3: Comparing the relationships of O*NET skills to city size, expected job impact, and labor specialization. **(A)** We plot Pearson correlation of raw skill importance to expected job impact (ρ_E) on the x-axis versus the Pearson correlation of raw skill importance to city skill entropy (ρ_H) on the y-axis. We see that which indicate job impact from automation also indicate decreased specialization in cities. **(B)** We plot Pearson correlation of raw skill importance to expected job impact (ρ_E) on the x-axis versus the Pearson correlation of raw skill importance to city size ($\rho_{\log(N)}$) on the y-axis. We see that which indicate job impact from automation also indicate smaller city sizes. **(C)** We plot Pearson correlation of raw skill importance to city skill entropy (ρ_H) on the x-axis versus the Pearson correlation of raw skill importance to city size ($\rho_{\log(N)}$) on the y-axis. The correlation between these two variables is not significant

cities are not significantly related to the skills which indicate city size. This finding is surprising given the other panels of the figure, and motivates us to consider the relationship between occupational specialization and city size through the jobs in each city (see main text).

3 Estimating the Affects of Automation

Automation and its impact on labor are increasingly important topics to researchers [7–9]. Examples throughout history, such as the industrial revolution and the advent of computers, demonstrate how technological advancement can lead to both job loss and job creation [10,11]. However, it is extremely difficult to predict how quickly a seemingly imminent technology will reach maturity and what the impact of that technology will be. For example, it's currently topical to discuss self-driving cars, but, while autonomous-capable cars are available for purchase, no self-driving cars are currently operated on the mass market. On the other hand, early leaders in computer hardware famously offered pessimistic predictions on the impact of computing:

- *"There is no reason anyone would want a computer in their home"* - Ken Olsen, founder of Digital Equipment Corporation (1977)
- *"I think there is a world market for maybe five computers"* - Thomas Watson, former president of IBM (1943)

3.1 Estimating Automation Impact using Frey/Osborne Data

Frey and Osborne [12] produced probabilities of computerization for each BLS job. They convened a workshop of leaders in automation to identify which of the BLS jobs were certainly automatable and certainly not-automatable. They used the O*NET skills dataset to identify the raw importance of nine workplace skills to each BLS job: Manual Dexterity, Finger Dexterity, Cramped Workspace/Awkward Positions, Originality, Fine Arts, Social Perceptiveness, Negotiation, Persuasion, and Assisting & Caring for Others. These O*NET skills represent "known bottlenecks to computerization." Using the importance of these skills to the jobs whose automatability was clear, they used a Gaussian process classifier to produce a probability of computerization for each BLS job.

Frey and Osborne used these probabilities to conclude that 47% of the current U.S. jobs are at "high risk" of computerization. n Several studies [13–15] utilize these same probabilities to investigate the impacts of automation, which highlights the utility of the probabilities despite the difficulty of the prediction undertaken in [12]. We use the same probabilities in combination with the distribution of BLS jobs across U.S. cities to add spatial resolution to their

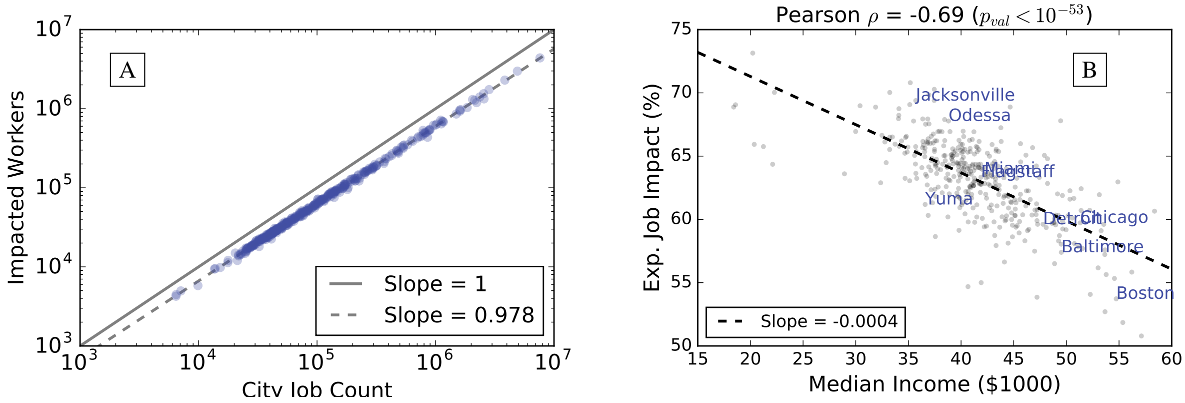

Figure 4: **(A)** The expected number of displaced workers grows slightly sublinearly ($\beta = 0.978$) with city size. **(B)** Expected job impact is anti-correlated with median income of cities according to U.S. Census.

findings. For a city, m, the expected job impact from automation is calculated according to

$$E_m = \sum_{j \in Jobs_m} p_{auto}(j) \cdot p_m(j), \tag{2}$$

where $p_{auto}(j)$ is the probability of computerization according to [12] and $p_m(j)$ is the proportion of workers in city m with job j. Table 6.1 demonstrates the ordered list of cities according to expected job impact.

As mentioned above, it's difficult to validate automation predictions. Nonetheless, our calculations for expected job impact represent an aggregate signal for the types of jobs in a city in relation to imminent automation technology. In the Table 6.1, we present the U.S. cities ordered by their expected job impact from automation. The list produces an ordering that appears to make sense; cities with technology companies and research institutes, such as Boston, M.A., and Boulder, C.O., have the lowest expected job impact, while cities relying on the tourist industry and agriculture, such as Myrtle Beach, S.C., and Napa, C.A., have the highest expected job impact. While the absolute proportions can only be validated with time, we believe the overall trend embodied in expected job impact in cities represents an underlying true signal.

To demonstrate the robustness of our results further, we perform two robustness checks to verify the negative trend between city size and expected job impact from automation (see Fig. 1B from the main text). The probability of computerization (i.e. $p_{auto}(j)$) from [12] are produced through a machine learning process applied to predictions of the automatability of jobs from experts. Therefore, we expect some errors in the predictions of these experts, and our task is to demonstrate that the error in the resulting $p_{auto}(j)$ would need to be substantial to invalidate our finding. We perform this analysis by artificially adding random noise to each $p_{auto}(j)$ according to

$$p^*_{auto}(j) = p_{auto}(j) + e_j, \tag{3}$$

where e_j is chosen uniformly at random from the interval $[-error, +error]$ for each occupation. For each choice of $error$, we perform 500 trials calculating a new $p^*_{auto}(j)$ for each occupation and recalculating the expected job impact from automation in each city according to

$$E^*_m = \sum_{j \in Jobs_m} p^*_{auto}(j) \cdot p_m(j) \tag{4}$$

similar to equation 2. Finally, we measure the Pearson correlation between $\log_{10}$ the total employment in each city and E^*_m so that we can compare to the empirical relationship we observe in Figure 1B of the main text (Pearson $\rho = -0.53$, $p_{val} < 10^{-28}$). Figure 5A demonstrates the results of this exercise. We find that substantial error ($error \approx 0.15$) needs to be added to the empirical probabilities of computerization for each occupation before our result from the main text no longer represents the observed trend. Even if we make the extremely strong assumption of $error = .5$, we would still observe a strong negative trend, and we would still conclude that small cities face greater impact from automation.

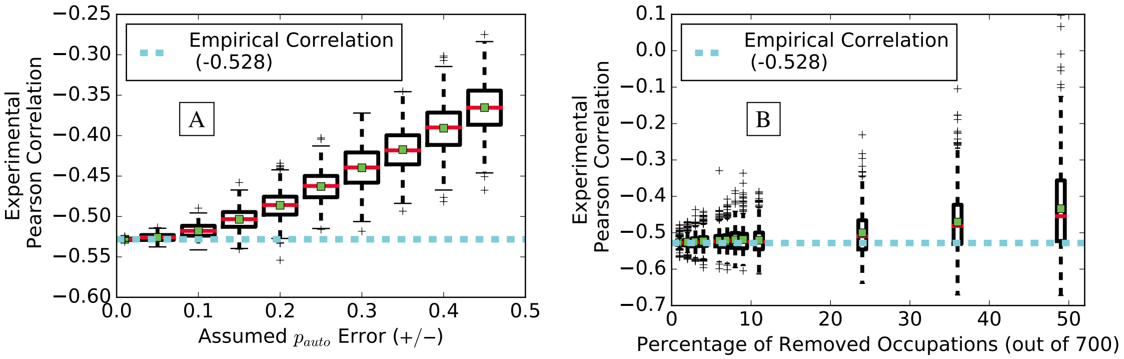

Figure 5: The relationship between city size and expected job impact from automation is robust. **(A)** For choice of assumed error in the predictions from [12], we perform 500 trials measuring the resulting Pearson correlation between $\log_{10}$ city size and expected job impact from automation after the error has been added to each occupation's probability of computerization (y-axis). **(B)** After selecting a proportion of occupations (x-axis), we perform 500 trials of randomly selecting that many occupations to remove while measuring the resulting Pearson correlation between $\log_{10}$ city size and the expected job impact from automation in cities (y-axis).

In the second robustness check, we test the robustness of our observed relationship between city size and expected job impact if a randomly selected subset of occupations are removed from the analysis. For each proportion of occupations to be removed, we perform 500 trials of randomly selecting occupations to be ignored and recalculate E_m using the $p_{auto}(j)$ presenting in [12]. We then measure the resulting Pearson correlation between these new E_m and $\log_{10}$ the total employment in each city. Figure 5B demonstrates that our empirical observation from Figure 1B in the main text holds even if very large proportions of occupations are ignored. In fact, only when we ignored half of all occupations did we observe any trials demonstrating a trend contrary to the one presented in the main text. Therefore, we conclude that small cities face greater impact from automation.

3.2 Estimating Automation Impact using OECD Data

The Organization for Economic Co-operation Development (OECD) released alternative estimates for the probability of job automation with a focus on job categories used by OECD countries [16]. Rather than the job-based approach used in [12], assessments on the automatability of workplace skills were derived. These skill assessments can be used in combination with government data relating the importance of skills to jobs to assess the likelihood of computerization for jobs. Contrary to the alarming 47% of jobs at "high risk of computerization" found by Frey and Osborne, these new probabilities produce a more mild conclusion of only 9%. These job probabilities were derived with OECD job definitions in mind, but collaborations between OECD and U.S. BLS have lead to an official mapping between the two job definitions. We utilize this mapping to assess the resilience of labor markets in cities as a function of city size in Figure 6. Despite the more conservative estimates in [16], our results remain; we again observe significantly decreased expected job impact in large cities (Fig. 6A).

3.3 Expected Job Impact & Labor Specialization in Cities

In Figure 7, we further characterize the relationship between a city's resilience to job impact from automation and labor specialization. We provide additional figures in the main text detailing how workplace skills explain the positive correlation we observe between labor specialization and resilience to job impact in cities. Here, we demonstrate that resilience to job impact is significantly correlated to the number of unique jobs in a city, and more weakly correlated to the Shannon entropy of job distributions. This weaker correlation motivates our investigation into workplace skills, in addition to the distribution of jobs, presented in the main text.

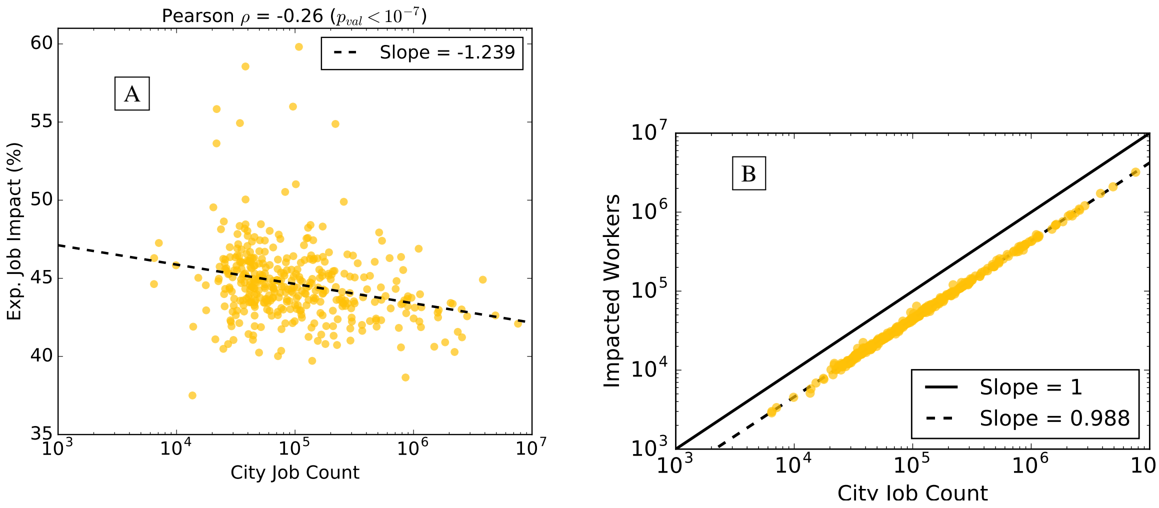

Figure 6: Expected job impact from automation decreases with city size using conservative estimates of job loss. **(A)** The expected job impact of cities decreases with city size. **(B)** The number of displaced workers per city grows slightly sublinearly with city size ($\beta = 0.988$).

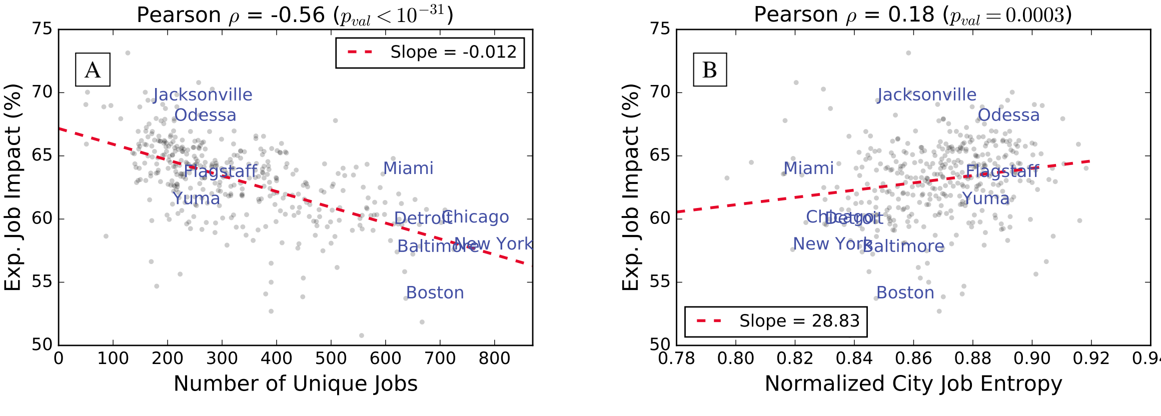

Figure 7: Characterizing the relationship between labor specialization and job impact from automation in cities. **(A)** Resilience to job impact is correlated with the number of unique jobs in cities. **(B)** Increased labor specialization according to job distributions in cities indicates increased resilience to job impact from automation.

3.4 Explaining Differences in Expected Job Impact

From equation 2, we may observe that both the automatability and the employment share of individual occupations contribute to the expected job impact from automation in a city. Correspondingly, we measure the difference in expected job impact for cities m and n according to

$$\begin{aligned} E_m - E_n &= \sum_{j \in Jobs} p_{auto}(j) \cdot \big(share_m(j) - share_n(j)\big) \\ &= \sum_{j \in Jobs} p_{auto}(j) \cdot \big(share_m(j) - share_n(j)\big) - \sum_{j \in Jobs} E_n \cdot \big(share_m(j) - share_n(j)\big) \\ &= \sum_{j \in Jobs} \big(p_{auto}(j) - E_n\big) \cdot \big(share_m(j) - share_n(j)\big), \end{aligned} \tag{5}$$

where we have utilized $\sum E_n \cdot (share_m(j) - share_n(j)) = 0$. Here, we let $Jobs$ denote the set of all occupation types across all cities, $p_{auto}(j)$ denotes the probability of computerization of occupation j according to [12], and $share_m(j)$ denotes the employment share of occupation j in city m. Equation 5 highlights that occupation j's influence on the difference in expected job impact in cities m and n falls into one of four categories:

1. occupation j is relatively resilient to automation (i.e. $(p_{auto}(j) - E_n) > 0$) and relatively more abundant in city m (i.e. $(share_m(j) - share_n(j)) > 0$),
2. occupation j is relatively susceptible to automation (i.e. $(p_{auto}(j) - E_n) < 0$) and relatively less abundant in city m (i.e. $(share_m(j) - share_n(j)) < 0$),
3. occupation j is relatively resilient to automation (i.e. $(p_{auto}(j) - E_n) > 0$) and relatively less abundant in city m (i.e. $(share_m(j) - share_n(j)) < 0$), or
4. occupation j is relatively susceptible to automation (i.e. $(p_{auto}(j) - E_n) < 0$) and relatively more abundant in city m (i.e. $(share_m(j) - share_n(j)) > 0$).

Occupations in categories 1 and 2 effectively increase $E_m - E_n$, while occupations in categories 3 and 4 effectively decrease the difference.

Let

$$\delta_{(m,n)}(j) = 100 \cdot \frac{\big(p_{auto}(j) - E_n\big) \cdot \big(share_m(j) - share_n(j)\big)}{E_m - E_n} \tag{6}$$

denote the percent influence of occupation j on the difference in expected job impact for cities m and n. Figure 8 demonstrates a visualization of equation 5 that we call an "occupation shift." Correspondingly, if we add the employment distributions in the 50 largest cities and 50 smallest cities together (respectively), then we can quantify how each occupation contributes to the differential impact of automation on employment in large and small cities. We present this occupation shift in Figure 9 (also Figure 5 of the main text).

Referring to the job clusters from Figure 5 in the main text, we see that purple occupations and blue occupations contribute the most to the difference in expected job impact, while green and yellow occupation types effectively diminish the difference in both occupation shifts. However, certain occupations, such occupations of the green job cluster, can both increase and decrease the difference between resilient and susceptible cities. The occupation shift allows us to understand which occupations explain the overall trend and which occupations go against the overall trend. If we had only considered occupations that add to the difference (i.e. occupations corresponding to dark colored bars on the right side of the plot), then we may have incorrectly concluded that the differences in relatively susceptible occupations explain the difference we observe in these two examples. This transparency can help urban policy makers determine how labor shifts in different industries may effect their preparedness for the impact of new technology.

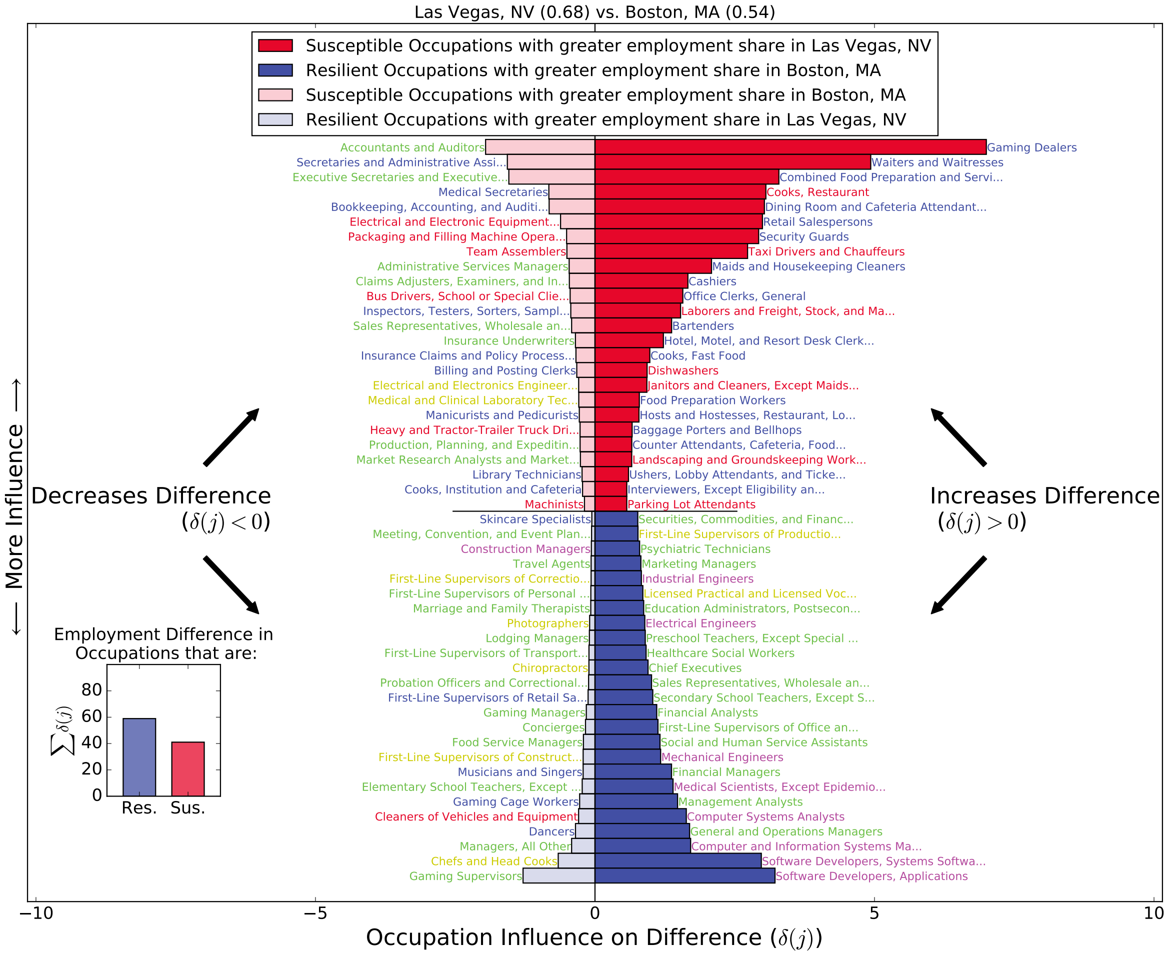

Figure 8: An occupation shift explaining the difference in expected job impact for Boston, MA ($E_m = 0.54$) compared to Las Vegas, NV ($E_m = 0.68$) using equation 5. Each horizontal bar represents $\delta_{(\text{Las Vegas,Boston})}(j)$ of occupation j. The occupation title is provided next to the corresponding bar and colored according to its job cluster as identified in Figure 4 of the main text. Red bars represent occupations with higher risk of computerization compared to Boston's expected job impact. Blue bars represent occupations with lower risk of computerization compared to Boston's expected job impact. Dark colors represent occupations that effectively increase the difference, while pale colors represent occupations that effectively decrease the difference in expect job impact. Bars in each of the quadrants are vertically ordered according to $|\delta_{(\text{Las Vegas,Boston})}(j)|$. The inset in the bottom left of the plot summarizes the overall influence of resilient occupations compared to occupations that are at risk of computerization.

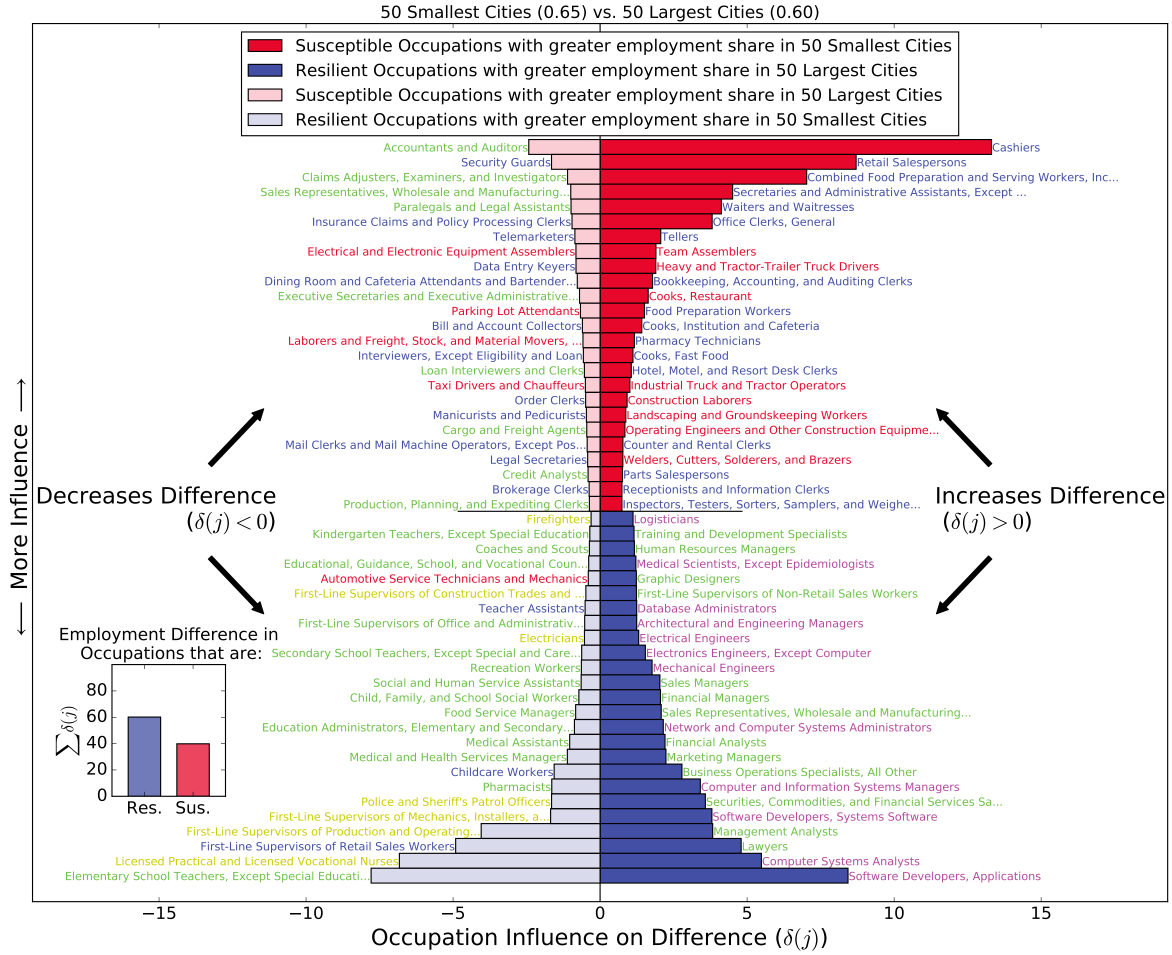

Figure 9: An occupation shift explaining the difference in expected job impact for the 50 largest cities ($E_m = 0.60$) compared to the 50 smallest cities ($E_m = 0.65$) using equation 5. Each horizontal bar represents $\delta_{(\text{Small Cities,Large Cities})}(j)$ of occupation j. The occupation title is provided next to the corresponding bar and colored according to its job cluster as identified in Figure 5 of the main text. Red bars represent occupations with higher risk of computerization compared to the expected job impact in large cities. Blue bars represent occupations with lower risk of computerization compared to the expected job impact in large cities. Dark colors represent occupations that effectively increase the difference, while pale colors represent occupations that effectively decrease the difference in expect job impact. Bars in each of the quadrants are vertically ordered according to $|\delta_{(\text{Small Cities,Large Cities})}(j)|$. The inset in the bottom left of the plot summarizes the overall influence of resilient occupations compared to occupations that are at risk of computerization.

3.5 Labor Specialization as a Mediator for City Size and Automation Impact

Our analysis in the main text predominantly relies on linear regression to explore the relationship between city size, labor specialization, and the expected impact of automation in cities (see Figures 1, 2, & 3). In particular, we find evidence that the relationship between city size and expected impact may be mediated by the labor specialization in cities. This conceptualization leads us to perform a formal mediation analysis [17] with city size as a treatment variable (i.e. $\log_{10}$ total employment in cities, denoted $size_m$), our various measures for labor specialization (i.e. $H_{job}(m)$, $H_{skill}(m)$, and $1 - T_m$) as independent mediators, and the expected impact from automation (i.e. E_m) as the outcome variable.

We take the generic urban variables from Figure 3 in the main text as additional control variables; these variables include the median household income ($income_m$), the per capita GDP (GDP_m), the percent of population with a bachelor's degree ($bachelor_m$), and the number of unique occupations in each city ($jobs_m$). The purpose of these control variables are to mitigate omitted variable bias, but the U.S. labor system is a sufficiently complicated system that omitted variable bias can never fully be controlled for. In our opinion, this observation limits the strength of any conclusion about causality [18].

This analysis is subject to further assumptions as well. Firstly, we are assuming that the effect of city size on job impact is constant across cities. Again, this assumption is extremely difficult to prove given the complexity of the U.S. labor system, regional geographies, regional politics, and economic trade. Secondly, the effects of unobserved causes for the mediator and outcome variables (denoted e_1 and e_2, respectively) are uncorrelated. In the analysis below, we measure the Pearson correlation between e_1 and e_2 for each choice of labor specialization measure.

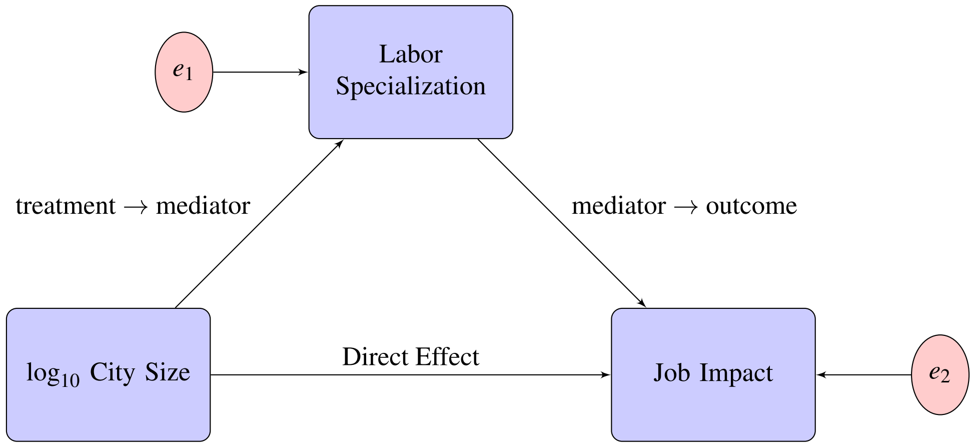

Figure 10: Schematic for mediation analysis. We consider the city size (i.e. $size_m$) as the treatment variables, as measure for labor specialization (i.e. $H_{job}(m)$, $H_{skill}(m)$, or $1 - T_m$) as the mediator variable, and the expected impact of automation on cities (E_m) as the outcome variable. e_1 represents the unobserved causes of labor specialization, while e_2 represents the unobserved causes of the impact of automation in cities.

Labor Specialization Variable	Avg. Causal Mediated Effect (ACME)	Avg. Direct Effect (ADE)	Total Effect	Pearson(e_1,e_2)
Job Specialization ($H_{job}(m)$	0.793^{***}	-0.516^{*}	0.277	7.38×10^{-16}
Skill Specialization ($H_{skill}(m)$)	-0.078	0.357	0.278	3.85×10^{-16}
Theil Entropy ($1 - T_m$)	-0.107^{*}	0.389	0.282	6.01×10^{-17}
* p-value $< .1$, ** p-value $< .01$, *** p-value $< .001$				

Table 1: The results of mediation analysis. All variables were standardized prior to analysis. Ignoring whether the necessary assumptions for mediation analysis are met, we find some evidence that job specialization ($H_{job}(m)$) may act as a mediator for the effect of city size on the expected impact of automation.

4 Robustness Check of the Linear Regression Model for E_m

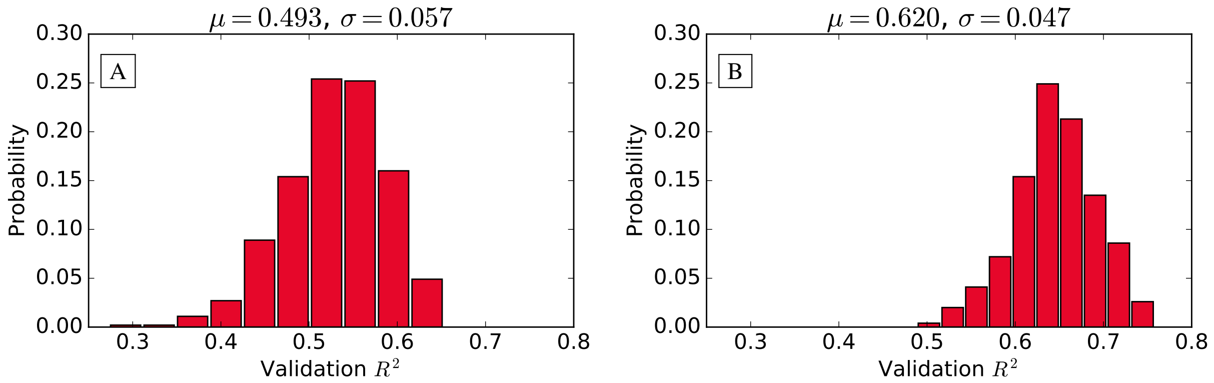

Figure 11: To confirm the validity of the regression models, we perform 1,000 trials where half of the cities are randomly selected without replacement as training data and the remaining cites are used for validation. We under go this process for the regression model using only generic urban indicators **(A)** and the regression model using all variables **(B)**. The resulting distributions of variance explained (R^2) when the trained models are applied to separate validation data confirms that the full regression model accounts for an additional 10% of variance on average.

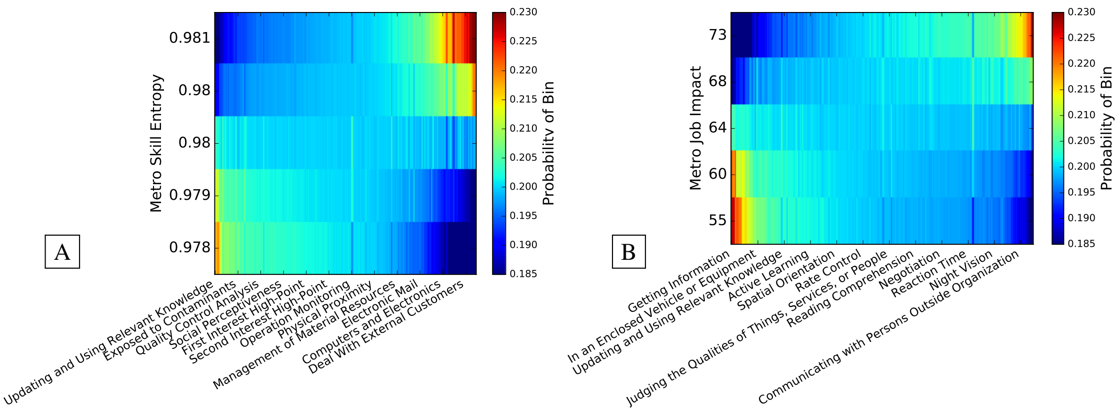

Figure 12: The shape of skills indicating specialization (left) and resilience to expected job displacement (right) in cities is maintained when observing raw O*NET skills in place of the aggregate skills used in the main text. The colors in each column indicate the probability of a city having the quality on the y-axis given an observation of a labor skill on the x-axis. We have labelled a few of the raw O*NET skills on the x-axis for reference.

5 Simplifying Jobs & Skills

In an effort to clearly identify how jobs contribute to labor specialization in larger cities, we identify aggregate job types based on common workplace skills. Previous studies, such as [1], examined the relationship between industry size and city size for various abstractions for industry according to NAICS. Here, we are seeking an organic representation of the forces in effect, and so we use K-means clustering based on the raw skill values for each job to identify five clusters of similar jobs (i.e. occupations are instances and the raw O*NET importance of each skill are features). These job groups represent collections of jobs which rely on similar skills for completion. The BLS jobs comprising each job type are shown in Section 6.3. Note that our results and interpretations are consistent for anywhere from three to seven clusters (see 6.3.1). This simplification of the space of jobs allows us to clearly understand which job types are disproportionately emphasized in large cities through the scaling behaviors of these job types.

We also seek to explain our results on the basis of workplace skills. To this end, we measure the correlation of raw skill values across all BLS jobs for each pair of O*NET skills and employ K-means clustering to identify ten groups of co-occurring skills (i.e. workplace skills are instances and the Pearson correlation of the raw O*NET importance of that skill to the importance of each other skill are the features). The complete lists of raw O*NET skills comprising each skill type are presented in Section 6.5. We summarize the skills comprising each skill type with the groups' titles. This simplification of the space of skills clarifies how different types of skills explain our results, and trends that we present using these aggregated skill groups are apparent when reproduced using the raw O*NET skills instead.

5.1 O*NET Task Groups

An alternative simplification of the raw O*NET skills is the O*NET Task Groups, which represent collections of similar work activities. We provide the definitions for these task groups in Table 3. These task groups have been used to investigate the task connectivity of urban labor markets in relation to employment growth [19]. In Figure 13, we use these groups as alternative skill aggregations and assess which tasks indicate resilience to job displacement from automation in cities (Fig. 13A), which tasks indicate occupational specialization in cities (Fig. 13B), and which tasks indicate superlinear scaling of job types (Fig. 13C). We find that Mental Process tasks are indicative of increased specialization in cities, increased resilience to job displacement in cities, and superlinear scaling of job types. On the other hand, Work Output tasks, which focuses on physical skills, indicate less specialization in cities, less resilience to job displacement in cities, and linear or sublinear scaling of job types. These findings are in agreement with the results in the main text.

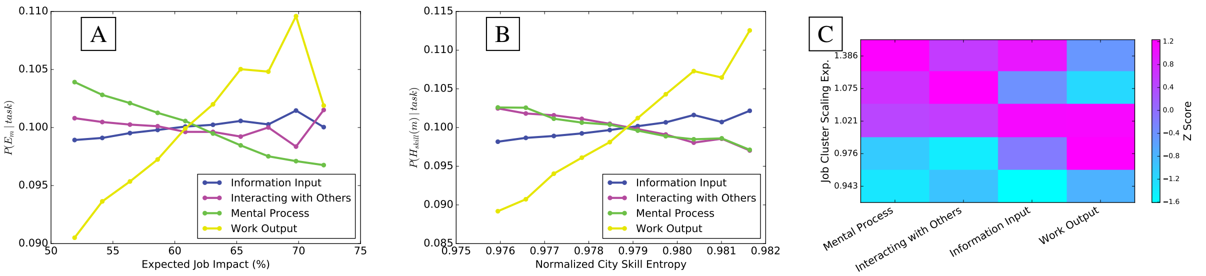

Figure 13: The relationships between O*NET tasks expected job impact from automation, labor specialization, and the scaling of job types. **(A)** We bin cities according to their expected job impact from automation (x-axis). For each task (legend), we normalize the importance of that task across bins to a probability $P(E_m \mid task)$ representing how strongly that task indicates each level of job displacement (y-axis). **(B)** We bin cities according to their skill specialization (x- axis) and sum the importance of each task for each bin. For each task (leg- end), we normalize the importance of that task across bins to a probability $P(H_{skill}(m) \mid task)$ representing how strongly that task indicates each level of specialization (y-axis). **(C)** By summing the importance of each task to each job type, we assess how strongly a task indicates a scaling relationship according to its z score. For a given task, z scores are calculated according to the distribution of importance across job clusters.

O*NET Task Group	Job Impact Corr.	Log_{10} City Size Corr.
Mental Process	-0.86 ($< 10^{-113}$)	0.67 ($< 10^{-49}$)
Interacting with Others	-0.46 ($< 10^{-20}$)	0.13 (0.01)
Information Input	-0.082 (0.11)	0.34 ($< 10^{-11}$)
Work Output	0.69 ($< 10^{-53}$)	-0.37 ($< 10^{-12}$)

Table 2: Summarizing the relationship between tasks, job impact from automation, and city size. In the middle (right) column, we present the Pearson correlation of the proportion of each task to the expected job impact (log 10 city size). We provide the associated p-values in parentheses

5.2 The Routineness of Tasks

Autor et al. [20, 21] identify workplace tasks according to their type and how routine the task is. They find that non-routine tasks are becoming increasingly important to workers relative to routine tasks. We provide the definitions for these task groups in Table 5. In Figure 14, we use these groups as alternative skill aggregations and assess which tasks indicate resilience to job impact from automation in cities (Fig. 14A), which tasks indicate occupational specialization

**Task Group**	**O*NET Skills**
Information Input	Getting Information, Monitor Processes, Materials, or Surroundings, Identifying Objects, Actions, and Events, Inspecting Equipment, Structures, or Material, Estimating the Quantifiable Characteristics of Products, Events, or Information
Mental Process	Judging the Qualities of Things, Services, or People, Processing Information, Evaluating Information to Determine Compliance with Standards, Analyzing Data or Information, Making Decisions and Solving Problems, Thinking Creatively, Updating and Using Relevant Knowledge, Developing Objectives and Strategies, Scheduling Work and Activities, Organizing, Planning, and Prioritizing Work
Work Output	Performing General Physical Activities, Handling and Moving Objects, Controlling Machines and Processes, Operating Vehicles, Mechanized Devices, or Equipment, Interacting With Computers, Drafting, Laying Out, and Specifying Technical Devices. Parts. and Equipment, Repairing and Maintaining Mechanical Equipment, Repairing and Maintaining Electronic Equipment, Documenting or Recording Information
Interacting with Others	Interpreting the Meaning of Information for Others, Communicating with Supervisors, Peers, or Subordinates, Communicating with Persons Outside Organization, Establishing and Maintaining Interpersonal Relationships, Assisting and Caring for Others, Selling or Influencing Others, Resolving Conflicts and Negotiating with Others, Performing for or Working Directly with the Public, Coordinating the Work and Activities of Others, Developing and Building Teams, Training and Teaching Others, Guiding, Directing, and Motivating Subordinates, Coaching and Developing Others, Provide Consultation and Advice to Others, Performing Administrative Activities, Staffing Organizational Units, Monitoring and Controlling Resources

Table 3: The O*NET skills comprising each Task Group.

in cities (Fig. 14B), and which tasks indicate superlinear scaling of job types (Fig. 14C). We find that all non-routine tasks are indicative of increased specialization in cities and increased resilience to job impact in cities. Non-routine analytic tasks and non-routine interactive tasks are indicative of superlinear scaling of job types, while non-routine manual tasks indicate linear or sublinear scaling of job types. Routine tasks indicate less specialization in cities, less resilience to job impact in cities, and linear or sublinear scaling of job types. These findings are in agreement with the results in the main text.

O*NET Task Type	Job Impact Corr.	Log_{10} City Size Corr.
Non-routine Analytic	-0.79 ($< 10^{-80}$)	0.50 ($< 10^{-24}$)
Non-routine Interactive	-0.73 ($< 10^{-62}$)	0.52 ($< 10^{26}$)
Routine Cognitive	0.47 ($< 10^{-21}$)	-0.14 (0.005)
Non-routine Manual	0.64 ($< 10^{-43}$)	-0.30 ($< 10^{-8}$)
Routine Manual	0.83 ($< 10^{-97}$)	-0.49 ($< 10^{-23}$)

Table 4: Summarizing the relationship between tasks, job impact, and city size. In the middle (right) column, we present the Pearson correlation of the proportion of each task to the expected job impact (log 10 city size). We provide the associated p-values in parentheses

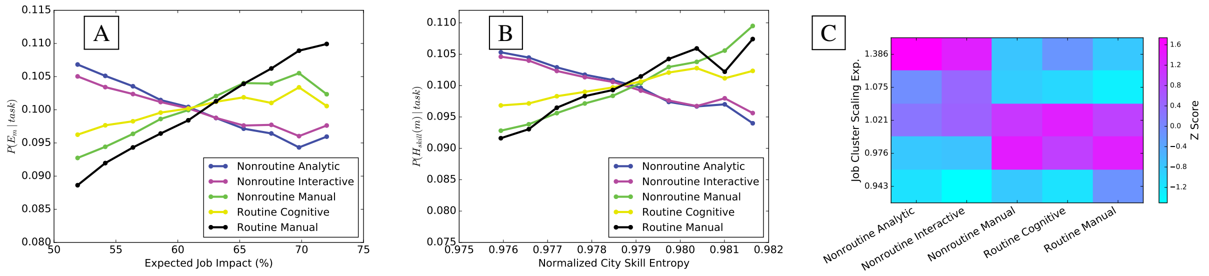

Figure 14: The relationships between O*NET tasks expected job impact from automation, labor specialization, and the scaling of job types. **(A)** We bin cities according to their expected job impact from automation (x-axis). For each task (legend), we normalize the importance of that task across bins to a probability $P(E_m \mid task)$ representing how strongly that task indicates each level of job displacement (y-axis). **(B)** We bin cities according to their skill specialization (x- axis) and sum the importance of each task for each bin. For each task (leg- end), we normalize the importance of that task across bins to a probability $P(H_{skill}(m) \mid task)$ representing how strongly that task indicates each level of specialization (y-axis). **(C)** By summing the importance of each task to each job type, we assess how strongly a task indicates a scaling relationship according to its z score. For a given task, z scores are calculated according to the distribution of importance across job clusters.

**Task Type**	**O*NET Skills**
Non-routine Analytic	Mathematical Reasoning, Mathematics, Deductive Reasoning, Number Facility, Physics, Programming
Non-routine Interactive	Design, Administration and Management, Economics and Accounting, Equipment Selection, Estimating the Quantifiable Characteristics of Products, Events, or Information, Importance of Being Exact or Accurate, Management of Financial Resources, Management of Material Resources, Management of Personnel Resources, Organizing, Planning, and Prioritizing Work, Personnel and Human Resources, Quality Control Analysis, Sales and Marketing, Scheduling Work and Activities, Technology Design, Visualization
Routine Cognitive	Consequence of Error, Control Precision, Controlling Machines and Processes, Documenting/Recording Information, Evaluating Information to Determine Compliance with Standards, Inspecting Equipment, Structures, or Material, Operation and Control, Quality Control Analysis
Routine Manual	Finger Dexterity, Manual Dexterity, Arm-Hand Steadiness, Wrist-Finger Speed
Non-routine Manual	Reaction Time, Response Orientation, Cramped Work Space, Awkward Positions, Dynamic Flexibility, Spatial Orientation, Transportation, Coordination

Table 5: The O*NET skills comprising each Task Type.

6 Data Tables

6.1 Cities Ordered by Expected Job Impact from Automation

Rank	Metro. Area	Exp. Job Impact (%)
1	San Jose-Sunnyvale-Santa Clara, CA	50.79
2	Washington-Arlington-Alexandria, DC-VA-MD-WV	51.85
3	Trenton-Ewing, NJ	52.71
4	Boston-Cambridge-Quincy, MA-NH	53.72
5	Durham-Chapel Hill, NC	53.85
6	Boulder, CO	54.07
7	Warner Robins, GA	54.69
8	Huntsville, AL	55.00
9	Bridgeport-Stamford-Norwalk, CT	55.33
10	Ithaca, NY	55.64
11	San Francisco-Oakland-Fremont, CA	55.84
12	Hartford-West Hartford-East Hartford, CT	56.17
13	Ann Arbor, MI	56.50
14	Corvallis, OR	56.63
15	Seattle-Tacoma-Bellevue, WA	57.23
16	Baltimore-Towson, MD	57.40
17	Madison, WI	57.49
18	New York-Northern New Jersey-Long Island, NY-NJ-PA	57.59
19	New Haven, CT	57.70
20	Minneapolis-St. Paul-Bloomington, MN-WI	57.85
21	Charlottesville, VA	57.93
22	Worcester, MA-CT	58.04
23	Albany-Schenectady-Troy, NY	58.09
24	Colorado Springs, CO	58.12
25	Denver-Aurora-Broomfield, CO	58.30
26	Burlington-South Burlington, VT	58.31
27	Raleigh-Cary, NC	58.62
28	Hinesville-Fort Stewart, GA	58.64
29	Springfield, MA-CT	58.71
30	Cedar Rapids, IA	58.73
31	Rochester, MN	58.93
32	Sacramento–Arden-Arcade–Roseville, CA	59.11
33	Richmond, VA	59.11
34	Tallahassee, FL	59.14
35	Bremerton-Silverdale, WA	59.18
36	Austin-Round Rock-San Marcos, TX	59.22
37	Portsmouth, NH-ME	59.24
38	Manchester, NH	59.36
39	Peoria, IL	59.38
40	Dayton, OH	59.39
41	Provo-Orem, UT	59.41
42	Tucson, AZ	59.47
43	Columbus, OH	59.51
44	Atlanta-Sandy Springs-Marietta, GA	59.54

45	Philadelphia-Camden-Wilmington, PA-NJ-DE-MD	59.57
46	San Diego-Carlsbad-San Marcos, CA	59.61
47	Waterbury, CT	59.62
48	Detroit-Warren-Livonia, MI	59.62
49	Springfield, IL	59.69
50	Chicago-Joliet-Naperville, IL-IN-WI	59.73
51	Olympia, WA	59.73
52	Danbury, CT	59.77
53	Little Rock-North Little Rock-Conway, AR	59.77
54	Palm Bay-Melbourne-Titusville, FL	59.80
55	Albuquerque, NM	59.80
56	Des Moines-West Des Moines, IA	59.83
57	Portland-Vancouver-Hillsboro, OR-WA	59.86
58	Pittsfield, MA	59.88
59	Rochester, NY	59.90
60	Leominster-Fitchburg-Gardner, MA	59.97
61	Phoenix-Mesa-Glendale, AZ	59.99
62	Providence-Fall River-Warwick, RI-MA	60.05
63	Topeka, KS	60.08
64	Norwich-New London, CT-RI	60.10
65	Columbia, SC	60.19
66	Jefferson City, MO	60.19
67	Baton Rouge, LA	60.20
68	Charlotte-Gastonia-Rock Hill, NC-SC	60.28
69	Bakersfield-Delano, CA	60.29
70	Salt Lake City, UT	60.29
71	Pascagoula, MS	60.31
72	Virginia Beach-Norfolk-Newport News, VA-NC	60.32
73	Kennewick-Pasco-Richland, WA	60.32
74	Milwaukee-Waukesha-West Allis, WI	60.34
75	Fort Collins-Loveland, CO	60.35
76	Cincinnati-Middletown, OH-KY-IN	60.36
77	Lansing-East Lansing, MI	60.38
78	Bloomington-Normal, IL	60.44
79	Portland-South Portland-Biddeford, ME	60.51
80	Anchorage, AK	60.65
81	Rochester-Dover, NH-ME	60.68
82	Honolulu, HI	60.70
83	Los Angeles-Long Beach-Santa Ana, CA	60.72
84	Syracuse, NY	60.74
85	Houston-Sugar Land-Baytown, TX	60.76
86	Cleveland-Elyria-Mentor, OH	60.82
87	Omaha-Council Bluffs, NE-IA	60.90
88	Oklahoma City, OK	60.91
89	Charleston-North Charleston-Summerville, SC	60.92
90	Jackson, MS	60.93
91	Champaign-Urbana, IL	61.06
92	Iowa City, IA	61.06
93	Akron, OH	61.14

94	Santa Fe, NM	61.15
95	Ogden-Clearfield, UT	61.15
96	Yuma, AZ	61.18
97	St. Louis, MO-IL	61.23
98	Bismarck, ND	61.27
99	Las Cruces, NM	61.31
100	Kansas City, MO-KS	61.31
101	Boise City-Nampa, ID	61.32
102	Indianapolis-Carmel, IN	61.33
103	Salem, OR	61.39
104	Cumberland, MD-WV	61.52
105	Dallas-Fort Worth-Arlington, TX	61.55
106	Duluth, MN-WI	61.57
107	Harrisburg-Carlisle, PA	61.58
108	Santa Barbara-Santa Maria-Goleta, CA	61.60
109	Poughkeepsie-Newburgh-Middletown, NY	61.66
110	Athens-Clarke County, GA	61.66
111	Nashville-Davidson–Murfreesboro–Franklin, TN	61.68
112	Battle Creek, MI	61.69
113	New Bedford, MA	61.75
114	Bangor, ME	61.76
115	Lincoln, NE	61.82
116	Columbus, GA-AL	61.82
117	Gainesville, FL	61.83
118	Binghamton, NY	61.87
119	Oxnard-Thousand Oaks-Ventura, CA	61.89
120	Utica-Rome, NY	61.90
121	Vallejo-Fairfield, CA	61.95
122	Chattanooga, TN-GA	61.96
123	Kalamazoo-Portage, MI	61.96
124	Lexington-Fayette, KY	61.97
125	Pittsburgh, PA	61.99
126	Killeen-Temple-Fort Hood, TX	61.99
127	Barnstable Town, MA	62.01
128	Fayetteville-Springdale-Rogers, AR-MO	62.02
129	Augusta-Richmond County, GA-SC	62.08
130	Johnstown, PA	62.10
131	Knoxville, TN	62.13
132	Pueblo, CO	62.20
133	Fayetteville, NC	62.20
134	Allentown-Bethlehem-Easton, PA-NJ	62.25
135	Buffalo-Niagara Falls, NY	62.26
136	Santa Cruz-Watsonville, CA	62.26
137	Cheyenne, WY	62.34
138	Florence, SC	62.34
139	Huntington-Ashland, WV-KY-OH	62.39
140	Santa Rosa-Petaluma, CA	62.42
141	Saginaw-Saginaw Township North, MI	62.45
142	Tampa-St. Petersburg-Clearwater, FL	62.49

143	San Antonio-New Braunfels, TX	62.52
144	Fresno, CA	62.56
145	Alexandria, LA	62.56
146	Tulsa, OK	62.58
147	Lynchburg, VA	62.59
148	Eugene-Springfield, OR	62.60
149	Morgantown, WV	62.60
150	Hagerstown-Martinsburg, MD-WV	62.64
151	Wichita, KS	62.67
152	Merced, CA	62.68
153	Vineland-Millville-Bridgeton, NJ	62.68
154	Rockford, IL	62.69
155	Albany, GA	62.73
156	Jackson, MI	62.75
157	Birmingham-Hoover, AL	62.77
158	Mankato-North Mankato, MN	62.78
159	Grand Rapids-Wyoming, MI	62.79
160	Crestview-Fort Walton Beach-Destin, FL	62.81
161	Salinas, CA	62.84
162	Oshkosh-Neenah, WI	62.84
163	Pine Bluff, AR	62.89
164	Toledo, OH	62.90
165	Green Bay, WI	62.91
166	College Station-Bryan, TX	62.92
167	Fairbanks, AK	62.96
168	Lewiston-Auburn, ME	63.03
169	Winston-Salem, NC	63.04
170	Pocatello, ID	63.05
171	Madera-Chowchilla, CA	63.05
172	Rome, GA	63.13
173	State College, PA	63.18
174	Evansville, IN-KY	63.21
175	Johnson City, TN	63.24
176	McAllen-Edinburg-Mission, TX	63.25
177	Beaumont-Port Arthur, TX	63.25
178	Clarksville, TN-KY	63.26
179	Yakima, WA	63.27
180	Davenport-Moline-Rock Island, IA-IL	63.28
181	Fargo, ND-MN	63.29
182	San Luis Obispo-Paso Robles, CA	63.32
183	Flagstaff, AZ	63.33
184	Visalia-Porterville, CA	63.34
185	St. Cloud, MN	63.36
186	Reading, PA	63.37
187	Salisbury, MD	63.39
188	Springfield, OH	63.41
189	New Orleans-Metairie-Kenner, LA	63.44
190	Memphis, TN-MS-AR	63.48
191	Kingston, NY	63.52

192	Canton-Massillon, OH	63.54
193	Spokane, WA	63.55
194	Miami-Fort Lauderdale-Pompano Beach, FL	63.56
195	Monroe, LA	63.57
196	Niles-Benton Harbor, MI	63.57
197	Jackson, TN	63.59
198	San Juan-Caguas-Guaynabo, PR	63.60
199	Lawton, OK	63.63
200	Flint, MI	63.63
201	Charleston, WV	63.63
202	Sherman-Denison, TX	63.64
203	Blacksburg-Christiansburg-Radford, VA	63.66
204	Yuba City, CA	63.67
205	Roanoke, VA	63.69
206	Manhattan, KS	63.76
207	Amarillo, TX	63.81
208	Steubenville-Weirton, OH-WV	63.86
209	Wilmington, NC	63.87
210	Greenville-Mauldin-Easley, SC	63.87
211	Pensacola-Ferry Pass-Brent, FL	63.92
212	Corpus Christi, TX	63.96
213	Tyler, TX	64.00
214	Kingsport-Bristol-Bristol, TN-VA	64.00
215	Redding, CA	64.00
216	Ames, IA	64.01
217	Carson City, NV	64.02
218	Jacksonville, FL	64.02
219	Appleton, WI	64.05
220	Decatur, IL	64.09
221	Wheeling, WV-OH	64.09
222	Scranton–Wilkes-Barre, PA	64.09
223	Chico, CA	64.10
224	Louisville-Jefferson County, KY-IN	64.11
225	Macon, GA	64.11
226	Eau Claire, WI	64.13
227	Decatur, AL	64.15
228	Idaho Falls, ID	64.15
229	Shreveport-Bossier City, LA	64.18
230	Springfield, MO	64.20
231	Medford, OR	64.20
232	Bloomington, IN	64.22
233	La Crosse, WI-MN	64.22
234	Billings, MT	64.22
235	Bellingham, WA	64.23
236	Glens Falls, NY	64.25
237	Missoula, MT	64.30
238	Coeur d'Alene, ID	64.32
239	Orlando-Kissimmee-Sanford, FL	64.33
240	South Bend-Mishawaka, IN-MI	64.34

241	Ponce, PR	64.36
242	Holland-Grand Haven, MI	64.37
243	Greensboro-High Point, NC	64.37
244	Lewiston, ID-WA	64.37
245	Mansfield, OH	64.38
246	Racine, WI	64.38
247	El Paso, TX	64.39
248	Bowling Green, KY	64.39
249	York-Hanover, PA	64.40
250	Waterloo-Cedar Falls, IA	64.42
251	Cleveland, TN	64.43
252	Gulfport-Biloxi, MS	64.45
253	Port St. Lucie, FL	64.46
254	Elizabethtown, KY	64.48
255	Brownsville-Harlingen, TX	64.48
256	Abilene, TX	64.49
257	Stockton, CA	64.54
258	Greeley, CO	64.57
259	Rocky Mount, NC	64.64
260	Longview, TX	64.64
261	Lima, OH	64.66
262	Spartanburg, SC	64.72
263	Parkersburg-Marietta-Vienna, WV-OH	64.76
264	Riverside-San Bernardino-Ontario, CA	64.79
265	Lubbock, TX	64.82
266	Lawrence, KS	64.84
267	Kankakee-Bradley, IL	64.91
268	Wichita Falls, TX	64.96
269	Terre Haute, IN	64.97
270	El Centro, CA	65.00
271	Greenville, NC	65.02
272	Erie, PA	65.03
273	Victoria, TX	65.05
274	Anderson, SC	65.06
275	Atlantic City-Hammonton, NJ	65.06
276	Mount Vernon-Anacortes, WA	65.08
277	Farmington, NM	65.09
278	Mobile, AL	65.10
279	Prescott, AZ	65.14
280	Hanford-Corcoran, CA	65.16
281	Sumter, SC	65.23
282	Lancaster, PA	65.24
283	Asheville, NC	65.24
284	North Port-Bradenton-Sarasota, FL	65.28
285	Fort Wayne, IN	65.31
286	Fort Smith, AR-OK	65.33
287	Janesville, WI	65.35
288	Montgomery, AL	65.35
289	Anderson, IN	65.35

290	Winchester, VA-WV	65.35
291	Lake Havasu City - Kingman, AZ	65.35
292	Savannah, GA	65.36
293	Altoona, PA	65.37
294	Modesto, CA	65.40
295	Youngstown-Warren-Boardman, OH-PA	65.41
296	Sheboygan, WI	65.47
297	Grand Forks, ND-MN	65.51
298	Joplin, MO	65.52
299	Bay City, MI	65.54
300	Columbia, MO	65.54
301	Sioux Falls, SD	65.55
302	Lakeland-Winter Haven, FL	65.58
303	Bend, OR	65.58
304	Muskegon-Norton Shores, MI	65.61
305	St. George, UT	65.64
306	Panama City-Lynn Haven-Panama City Beach, FL	65.66
307	Houma-Bayou Cane-Thibodaux, LA	65.67
308	Midland, TX	65.67
309	Dover, DE	65.69
310	Texarkana-Texarkana, TX-AR	65.72
311	Logan, UT-ID	65.75
312	Aguadilla-Isabela-San Sebastian, PR	65.75
313	Casper, WY	65.76
314	Goldsboro, NC	65.78
315	Sioux City, IA-NE-SD	65.82
316	Brunswick, GA	65.87
317	Longview, WA	65.92
318	Monroe, MI	65.93
319	Guayama, PR	65.93
320	Waco, TX	65.94
321	Hattiesburg, MS	65.95
322	Reno-Sparks, NV	65.98
323	Muncie, IN	66.01
324	Fond du Lac, WI	66.01
325	Lake Charles, LA	66.03
326	Rapid City, SD	66.10
327	Valdosta, GA	66.12
328	Dubuque, IA	66.13
329	Wenatchee-East Wenatchee, WA	66.16
330	Lafayette, IN	66.16
331	St. Joseph, MO-KS	66.16
332	Morristown, TN	66.20
333	Sandusky, OH	66.21
334	Owensboro, KY	66.21
335	Wausau, WI	66.23
336	Elmira, NY	66.25
337	Grand Junction, CO	66.25
338	Tuscaloosa, AL	66.32

339	Hickory-Lenoir-Morganton, NC	66.37
340	Jonesboro, AR	66.48
341	Danville, VA	66.50
342	Florence-Muscle Shoals, AL	66.51
343	Cape Coral-Fort Myers, FL	66.55
344	Cape Girardeau-Jackson, MO-IL	66.56
345	Deltona-Daytona Beach-Ormond Beach, FL	66.57
346	Dothan, AL	66.64
347	Great Falls, MT	66.75
348	Naples-Marco Island, FL	66.93
349	Columbus, IN	66.93
350	Gainesville, GA	66.93
351	Kokomo, IN	66.94
352	Hot Springs, AR	66.95
353	Lafayette, LA	66.97
354	Williamsport, PA	66.97
355	Ocean City, NJ	67.24
356	Anniston-Oxford, AL	67.53
357	Sebastian-Vero Beach, FL	67.55
358	Auburn-Opelika, AL	67.77
359	Odessa, TX	67.78
360	Las Vegas-Paradise, NV	67.79
361	Lebanon, PA	67.89
362	Burlington, NC	67.94
363	Danville, IL	67.94
364	San Angelo, TX	67.96
365	Ocala, FL	68.23
366	Laredo, TX	68.75
367	Gadsden, AL	68.87
368	San German-Cabo Rojo, PR	68.88
369	Napa, CA	68.88
370	Palm Coast, FL	68.98
371	Yauco, PR	69.06
372	Dalton, GA	69.07
373	Jacksonville, NC	69.39
374	Michigan City-La Porte, IN	69.40
375	Harrisonburg, VA	69.84
376	Punta Gorda, FL	70.03
377	Fajardo, PR	70.04
378	Elkhart-Goshen, IN	70.28
379	Myrtle Beach-North Myrtle Beach-Conway, SC	70.80
380	Mayaguez, PR	73.14

6.2 Relating City Trends to BLS Jobs

We present BLS jobs ordered by decreasing skill specialization in Table 6.2. We also provide the scaling exponent of each BLS job, along with the Pearson correlation of the relative abundance of each job to the expected job impact from automation (discussed below) across cities. p-values for the correlations are presented in parentheses.

**Rank**	**Job Title**	H_j	β	**Corr. to Job Impact**

1	Statisticians	0.949	0.748	-0.434 (0)
2	Telemarketers	0.951	0.955	0.233 (0)
3	Securities, Commodities, and Financial Services Sales Agents	0.955	1.128	-0.265 (0)
4	Loan Interviewers and Clerks	0.955	1.005	0.060 ($< 10^{-54}$)
5	Actuaries	0.956	0.756	-0.193 ($< 10^{-103}$)
6	Court, Municipal, and License Clerks	0.956	0.762	0.176 (0)
7	Court Reporters	0.957	0.637	0.140 ($< 10^{-65}$)
8	Credit Counselors	0.957	0.825	0.220 ($< 10^{-230}$)
9	Medical Transcriptionists	0.957	0.696	0.341 (0)
10	Financial Managers	0.958	1.103	-0.493 (0)
11	Training and Development Specialists	0.958	1.054	-0.316 (0)
12	Billing and Posting Clerks	0.958	0.972	0.145 (0)
13	Credit Authorizers, Checkers, and Clerks	0.958	0.792	0.192 ($< 10^{-239}$)
14	Legal Secretaries	0.958	0.990	0.053 ($< 10^{-41}$)
15	Clinical, Counseling, and School Psychologists	0.958	0.861	-0.087 ($< 10^{-103}$)
16	Operations Research Analysts	0.959	0.985	-0.282 (0)
17	Eligibility Interviewers, Government Programs	0.959	0.821	0.119 ($< 10^{-180}$)
18	Bookkeeping, Accounting, and Auditing Clerks	0.959	0.958	0.098 ($< 10^{-183}$)
19	Demonstrators and Product Promoters	0.959	0.718	0.283 (0)
20	Marriage and Family Therapists	0.959	0.610	0.280 (0)
21	Financial Examiners	0.959	0.910	0.006 ($< 10^{0}$)
22	Insurance Claims and Policy Processing Clerks	0.959	1.031	-0.055 ($< 10^{-33}$)
23	Judges, Magistrate Judges, and Magistrates	0.959	0.601	0.327 (0)
24	Payroll and Timekeeping Clerks	0.960	0.949	0.135 (0)
25	Accountants and Auditors	0.960	1.111	-0.458 (0)
26	Cost Estimators	0.960	0.986	0.059 ($< 10^{-61}$)
27	Administrative Law Judges, Adjudicators, and Hearing Officers	0.960	0.572	0.132 ($< 10^{-63}$)
28	Word Processors and Typists	0.960	0.600	0.226 (0)
29	Budget Analysts	0.960	0.795	-0.123 ($< 10^{-128}$)
30	Paralegals and Legal Assistants	0.960	1.093	-0.213 (0)
31	Office Clerks, General	0.960	0.934	0.196 (0)
32	Computer Programmers	0.960	1.168	-0.501 (0)
33	Procurement Clerks	0.961	0.845	0.142 ($< 10^{-261}$)
34	Crossing Guards	0.961	0.738	0.277 (0)
35	Executive Secretaries and Executive Administrative Assistants	0.961	1.093	-0.341 (0)
36	Real Estate Brokers	0.961	0.706	0.249 ($< 10^{-321}$)
37	Insurance Sales Agents	0.961	1.021	0.051 ($< 10^{-47}$)
38	Mental Health Counselors	0.961	0.814	-0.008 ($< 10^{0}$)
39	Loan Officers	0.961	1.033	-0.012 ($< 10^{-2}$)
40	Lawyers	0.961	1.226	-0.473 (0)
41	Financial Analysts	0.962	1.270	-0.529 (0)
42	Travel Agents	0.962	1.056	0.042 ($< 10^{-13}$)
43	Statistical Assistants	0.962	0.402	0.313 ($< 10^{-274}$)
44	Health Educators	0.962	0.793	-0.084 ($< 10^{-73}$)
45	Title Examiners, Abstractors, and Searchers	0.962	0.806	0.313 (0)
46	Software Developers, Applications	0.963	1.304	-0.663 (0)
47	Compensation, Benefits, and Job Analysis Specialists	0.963	1.037	-0.391 (0)
48	Medical Records and Health Information Technicians	0.963	0.893	0.073 ($< 10^{-92}$)
49	Educational, Guidance, School, and Vocational Counselors	0.963	0.919	-0.097 ($< 10^{-170}$)

50	Personal Financial Advisors	0.963	1.132	-0.356 (0)
51	Public Relations and Fundraising Managers	0.963	0.909	-0.405 (0)
52	Healthcare Social Workers	0.964	0.885	-0.053 ($< 10^{-46}$)
53	Psychologists, All Other	0.964	0.552	0.194 ($< 10^{-102}$)
54	Bill and Account Collectors	0.964	1.129	0.028 ($< 10^{-13}$)
55	Market Research Analysts and Marketing Specialists	0.964	1.237	-0.575 (0)
56	Compensation and Benefits Managers	0.964	0.877	-0.449 (0)
57	Software Developers, Systems Software	0.964	1.233	-0.612 (0)
58	Human Resources Assistants, Except Payroll and Timekeeping	0.964	0.937	0.026 ($< 10^{-11}$)
59	Elementary School Teachers, Except Special Education	0.964	0.855	0.226 (0)
60	Human Resources Managers	0.964	1.025	-0.348 (0)
61	Managers, All Other	0.964	1.040	-0.298 (0)
62	Technical Writers	0.964	0.888	-0.382 (0)
63	Library Technicians	0.964	0.765	-0.003 ($< 10^{0}$)
64	Speech-Language Pathologists	0.964	0.884	0.080 ($< 10^{-105}$)
65	Photographic Process Workers and Processing Machine Operators	0.964	0.695	0.148 ($< 10^{-107}$)
66	Chief Executives	0.964	0.977	-0.072 ($< 10^{-88}$)
67	Credit Analysts	0.964	1.039	-0.064 ($< 10^{-31}$)
68	Receptionists and Information Clerks	0.965	0.955	0.136 (0)
69	Tax Examiners and Collectors, and Revenue Agents	0.965	0.843	-0.054 ($< 10^{-24}$)
70	Education Administrators, Postsecondary	0.965	0.792	-0.000 ($< 10^{0}$)
71	Switchboard Operators, Including Answering Service	0.965	0.938	0.247 (0)
72	Financial Specialists, All Other	0.965	0.966	-0.258 (0)
73	Insurance Underwriters	0.965	0.887	-0.053 ($< 10^{-18}$)
74	Secretaries and Administrative Assistants, Except Legal, Medical, and Executive	0.965	0.921	0.221 (0)
75	Advertising Sales Agents	0.965	0.991	0.066 ($< 10^{-64}$)
76	Security Guards	0.965	1.161	0.053 ($< 10^{-51}$)
77	Producers and Directors	0.965	1.057	-0.171 ($< 10^{-267}$)
78	Claims Adjusters, Examiners, and Investigators	0.965	1.137	-0.104 ($< 10^{-134}$)
79	Brokerage Clerks	0.965	1.038	-0.082 ($< 10^{-42}$)
80	First-Line Supervisors of Non-Retail Sales Workers	0.965	1.088	-0.075 ($< 10^{-99}$)
81	Interviewers, Except Eligibility and Loan	0.965	0.934	0.038 ($< 10^{-17}$)
82	Merchandise Displayers and Window Trimmers	0.965	0.870	0.218 (0)
83	Molding, Coremaking, and Casting Machine Setters, Operators, and Tenders, Metal and Plastic	0.965	0.624	0.288 (0)
84	New Accounts Clerks	0.966	0.701	0.396 (0)
85	Transportation, Storage, and Distribution Managers	0.966	0.961	0.140 ($< 10^{-269}$)
86	Marketing Managers	0.966	1.163	-0.549 (0)
87	Public Relations Specialists	0.966	1.052	-0.398 (0)
88	Education Administrators, Elementary and Secondary School	0.966	0.885	0.086 ($< 10^{-135}$)
89	Kindergarten Teachers, Except Special Education	0.966	0.841	0.217 (0)
90	Writers and Authors	0.966	0.911	-0.384 (0)
91	Police, Fire, and Ambulance Dispatchers	0.966	0.781	0.246 (0)
92	Massage Therapists	0.966	0.852	0.163 ($< 10^{-244}$)
93	Sales Representatives, Wholesale and Manufacturing, Except Technical and Scientific Products	0.966	1.082	0.155 (0)
94	Ushers, Lobby Attendants, and Ticket Takers	0.966	1.084	0.114 ($< 10^{-88}$)
95	Music Directors and Composers	0.966	0.555	0.297 (0)
96	Probation Officers and Correctional Treatment Specialists	0.966	0.769	0.187 ($< 10^{-308}$)

97	Property, Real Estate, and Community Association Managers	0.966	0.986	0.173 (0)
98	Radio and Television Announcers	0.966	0.557	0.417 (0)
99	Health Diagnosing and Treating Practitioners, All Other	0.966	0.786	-0.155 ($< 10^{-105}$)
100	Editors	0.966	0.956	-0.285 (0)
101	Database Administrators	0.967	1.087	-0.229 (0)
102	Order Clerks	0.967	1.001	0.104 ($< 10^{-157}$)
103	Business Operations Specialists, All Other	0.967	1.117	-0.468 (0)
104	Management Analysts	0.967	1.205	-0.460 (0)
105	Mechanical Drafters	0.967	0.751	0.347 (0)
106	Mental Health and Substance Abuse Social Workers	0.967	0.749	0.098 ($< 10^{-118}$)
107	Advertising and Promotions Managers	0.967	0.940	-0.155 ($< 10^{-121}$)
108	Cartographers and Photogrammetrists	0.967	0.446	-0.146 ($< 10^{-68}$)
109	Data Entry Keyers	0.967	1.076	-0.090 ($< 10^{-123}$)
110	Medical Secretaries	0.967	0.906	0.114 ($< 10^{-241}$)
111	Computer Hardware Engineers	0.967	0.689	-0.355 (0)
112	Rehabilitation Counselors	0.967	0.770	0.010 ($< 10^{0}$)
113	Instructional Coordinators	0.967	0.884	-0.119 ($< 10^{-209}$)
114	Cargo and Freight Agents	0.967	0.869	0.279 (0)
115	Stock Clerks and Order Fillers	0.967	0.936	0.307 (0)
116	Physical Scientists, All Other	0.967	0.466	-0.231 ($< 10^{-137}$)
117	Directors, Religious Activities and Education	0.967	0.489	0.243 ($< 10^{-206}$)
118	Secondary School Teachers, Except Special and Career/Technical Education	0.967	0.918	0.103 ($< 10^{-175}$)
119	Special Education Teachers, Secondary School	0.967	0.850	0.064 ($< 10^{-52}$)
120	Education Administrators, Preschool and Childcare Center/Program	0.967	0.819	-0.227 (0)
121	Parts Salespersons	0.968	0.847	0.384 (0)
122	Weighers, Measurers, Checkers, and Samplers, Recordkeeping	0.968	0.749	0.350 (0)
123	Musicians and Singers	0.968	0.738	0.077 ($< 10^{-25}$)
124	Social and Community Service Managers	0.968	0.835	-0.153 (0)
125	Training and Development Managers	0.968	0.894	-0.233 ($< 10^{-313}$)
126	Social Scientists and Related Workers, All Other	0.968	0.536	-0.182 ($< 10^{-144}$)
127	Interpreters and Translators	0.968	0.772	0.139 ($< 10^{-138}$)
128	Meeting, Convention, and Event Planners	0.968	0.933	-0.113 ($< 10^{-131}$)
129	Child, Family, and School Social Workers	0.968	0.883	0.011 ($< 10^{-2}$)
130	Bailiffs	0.968	0.409	0.397 (0)
131	Mail Clerks and Mail Machine Operators, Except Postal Service	0.968	0.952	-0.023 ($< 10^{-5}$)
132	Biological Scientists, All Other	0.968	0.534	-0.082 ($< 10^{-27}$)
133	Special Education Teachers, Middle School	0.968	0.820	0.120 ($< 10^{-148}$)
134	Appraisers and Assessors of Real Estate	0.968	0.765	0.361 (0)
135	Tellers	0.968	0.850	0.458 (0)
136	Teacher Assistants	0.968	0.894	-0.076 ($< 10^{-109}$)
137	Network and Computer Systems Administrators	0.968	1.149	-0.564 (0)
138	First-Line Supervisors of Office and Administrative Support Workers	0.968	0.989	-0.028 ($< 10^{-15}$)
139	Manicurists and Pedicurists	0.968	0.942	-0.213 ($< 10^{-168}$)
140	Concierges	0.968	0.861	0.291 ($< 10^{-292}$)
141	Food and Tobacco Roasting, Baking, and Drying Machine Operators and Tenders	0.968	0.384	0.364 (0)
142	Print Binding and Finishing Workers	0.968	0.667	0.306 (0)
143	Sales Managers	0.968	1.126	-0.267 (0)
144	Curators	0.968	0.518	-0.147 ($< 10^{-72}$)

145	Reporters and Correspondents	0.969	0.762	0.061 ($< 10^{-26}$)
146	Waiters and Waitresses	0.969	0.948	0.306 (0)
147	Pharmacists	0.969	0.921	0.179 (0)
148	Bus Drivers, School or Special Client	0.969	0.887	0.151 (0)
149	Locker Room, Coatroom, and Dressing Room Attendants	0.969	0.689	0.403 (0)
150	Social and Human Service Assistants	0.969	0.901	-0.105 ($< 10^{-192}$)
151	Graphic Designers	0.969	1.063	-0.304 (0)
152	Furnace, Kiln, Oven, Drier, and Kettle Operators and Tenders	0.969	0.433	0.368 (0)
153	Furniture Finishers	0.969	0.276	0.416 (0)
154	Clergy	0.969	0.693	0.072 ($< 10^{-45}$)
155	Purchasing Managers	0.969	0.924	-0.119 ($< 10^{-146}$)
156	Adult Basic and Secondary Education and Literacy Teachers and Instructors	0.969	0.569	0.187 ($< 10^{-241}$)
157	Reservation and Transportation Ticket Agents and Travel Clerks	0.969	1.078	0.187 ($< 10^{-178}$)
158	Skincare Specialists	0.969	0.798	0.304 (0)
159	Art Directors	0.969	0.974	-0.186 ($< 10^{-176}$)
160	Customer Service Representatives	0.969	1.069	-0.115 ($< 10^{-249}$)
161	Sales Representatives, Wholesale and Manufacturing, Technical and Scientific Products	0.969	1.189	-0.409 (0)
162	Team Assemblers	0.969	0.882	0.306 (0)
163	Computer and Information Systems Managers	0.969	1.219	-0.718 (0)
164	Hotel, Motel, and Resort Desk Clerks	0.969	0.788	0.313 (0)
165	Aerospace Engineers	0.969	0.478	-0.236 ($< 10^{-164}$)
166	Health and Safety Engineers, Except Mining Safety Engineers and Inspectors	0.969	0.634	0.133 ($< 10^{-94}$)
167	Tax Preparers	0.969	0.845	0.214 (0)
168	Private Detectives and Investigators	0.969	0.555	0.194 ($< 10^{-107}$)
169	Computer and Information Research Scientists	0.969	0.440	-0.343 ($< 10^{-320}$)
170	Civil Engineering Technicians	0.970	0.721	0.148 ($< 10^{-256}$)
171	Printing Press Operators	0.970	0.930	0.126 ($< 10^{-243}$)
172	Biological Technicians	0.970	0.632	-0.081 ($< 10^{-44}$)
173	Milling and Planing Machine Setters, Operators, and Tenders, Metal and Plastic	0.970	0.538	0.315 (0)
174	Electrical and Electronics Drafters	0.970	0.798	0.001 ($< 10^{0}$)
175	Medical Scientists, Except Epidemiologists	0.970	0.937	-0.475 (0)
176	Insurance Appraisers, Auto Damage	0.970	0.490	0.332 ($< 10^{-297}$)
177	Real Estate Sales Agents	0.970	0.929	0.247 (0)
178	Library Assistants, Clerical	0.970	0.777	0.067 ($< 10^{-60}$)
179	Environmental Scientists and Specialists, Including Health	0.970	0.796	-0.131 ($< 10^{-200}$)
180	Counter Attendants, Cafeteria, Food Concession, and Coffee Shop	0.970	0.958	0.099 ($< 10^{-137}$)
181	Civil Engineers	0.970	1.039	-0.233 (0)
182	Librarians	0.970	0.873	-0.031 ($< 10^{-16}$)
183	Construction Managers	0.970	1.027	-0.002 ($< 10^{0}$)
184	Mechanical Engineers	0.970	0.971	-0.166 (0)
185	Commercial and Industrial Designers	0.970	0.644	0.224 ($< 10^{-251}$)
186	Hairdressers, Hairstylists, and Cosmetologists	0.970	0.952	0.013 ($< 10^{-2}$)
187	Recreational Therapists	0.970	0.485	0.222 ($< 10^{-260}$)
188	Couriers and Messengers	0.970	0.834	0.236 (0)
189	Helpers–Production Workers	0.970	0.864	0.307 (0)
190	Lathe and Turning Machine Tool Setters, Operators, and Tenders, Metal and Plastic	0.970	0.503	0.317 (0)

191	Residential Advisors	0.970	0.692	0.209 (0)
192	Psychiatric Technicians	0.970	0.412	0.206 ($< 10^{-182}$)
193	Film and Video Editors	0.971	0.916	0.089 ($< 10^{-22}$)
194	Substance Abuse and Behavioral Disorder Counselors	0.971	0.726	0.125 ($< 10^{-180}$)
195	Logisticians	0.971	0.936	-0.237 (0)
196	Architectural and Civil Drafters	0.971	0.934	0.022 ($< 10^{-5}$)
197	Chemical Technicians	0.971	0.760	0.155 ($< 10^{-221}$)
198	Biomedical Engineers	0.971	0.645	-0.565 (0)
199	Wholesale and Retail Buyers, Except Farm Products	0.971	0.986	-0.048 ($< 10^{-27}$)
200	Computer Systems Analysts	0.971	1.277	-0.584 (0)
201	Engineers, All Other	0.971	0.894	-0.262 (0)
202	Chemical Engineers	0.971	0.524	0.207 ($< 10^{-215}$)
203	Occupational Therapists	0.971	0.879	0.037 ($< 10^{-21}$)
204	Pharmacy Aides	0.971	0.627	0.399 (0)
205	Middle School Teachers, Except Special and Career/Technical Education	0.971	0.926	0.138 ($< 10^{-302}$)
206	Purchasing Agents, Except Wholesale, Retail, and Farm Products	0.971	1.047	-0.296 (0)
207	Computer Operators	0.971	0.858	0.041 ($< 10^{-14}$)
208	Postmasters and Mail Superintendents	0.971	0.348	0.344 (0)
209	Fitness Trainers and Aerobics Instructors	0.971	0.952	-0.204 (0)
210	Administrative Services Managers	0.971	1.020	-0.288 (0)
211	Medical Assistants	0.971	0.951	0.124 ($< 10^{-287}$)
212	Urban and Regional Planners	0.971	0.670	0.031 ($< 10^{-7}$)
213	Medical and Health Services Managers	0.971	0.924	-0.178 (0)
214	Natural Sciences Managers	0.971	0.711	-0.371 (0)
215	Preschool Teachers, Except Special Education	0.972	0.956	-0.145 (0)
216	Dietitians and Nutritionists	0.972	0.844	0.031 ($< 10^{-11}$)
217	Production, Planning, and Expediting Clerks	0.972	1.022	0.002 ($< 10^{0}$)
218	Lifeguards, Ski Patrol, and Other Recreational Protective Service Workers	0.972	0.908	0.151 ($< 10^{-241}$)
219	Floral Designers	0.972	0.751	0.296 (0)
220	Postal Service Mail Carriers	0.972	0.891	0.253 (0)
221	Multimedia Artists and Animators	0.972	1.012	-0.244 ($< 10^{-196}$)
222	Architectural and Engineering Managers	0.972	1.039	-0.390 (0)
223	Chemical Equipment Operators and Tenders	0.972	0.508	0.305 (0)
224	Soil and Plant Scientists	0.972	0.166	0.116 ($< 10^{-38}$)
225	Sewing Machine Operators	0.972	0.743	0.277 (0)
226	Chemists	0.972	0.881	-0.200 (0)
227	Psychiatric Aides	0.972	0.322	0.331 (0)
228	Inspectors, Testers, Sorters, Samplers, and Weighers	0.972	0.906	0.267 (0)
229	Tour Guides and Escorts	0.972	0.551	0.363 (0)
230	Maids and Housekeeping Cleaners	0.972	0.907	0.260 (0)
231	Compliance Officers	0.972	1.039	-0.111 ($< 10^{-217}$)
232	Microbiologists	0.972	0.531	-0.128 ($< 10^{-47}$)
233	Woodworking Machine Setters, Operators, and Tenders, Except Sawing	0.972	0.410	0.492 (0)
234	Funeral Attendants	0.973	0.509	0.452 (0)
235	Sawing Machine Setters, Operators, and Tenders, Wood	0.973	0.320	0.472 (0)
236	Paper Goods Machine Setters, Operators, and Tenders	0.973	0.514	0.251 (0)
237	Cutting and Slicing Machine Setters, Operators, and Tenders	0.973	0.514	0.411 (0)
238	Helpers–Electricians	0.973	0.640	0.441 (0)

239	Electrical and Electronic Equipment Assemblers	0.973	0.914	-0.030 ($< 10^{-8}$)
240	Postal Service Mail Sorters, Processors, and Processing Machine Operators	0.973	0.975	-0.023 ($< 10^{-5}$)
241	Interior Designers	0.973	1.038	0.080 ($< 10^{-40}$)
242	Physical Therapists	0.973	0.934	-0.057 ($< 10^{-56}$)
243	Industrial Engineering Technicians	0.973	0.600	0.136 ($< 10^{-155}$)
244	First-Line Supervisors of Correctional Officers	0.973	0.456	0.195 ($< 10^{-236}$)
245	Hosts and Hostesses, Restaurant, Lounge, and Coffee Shop	0.973	0.946	0.246 (0)
246	Buyers and Purchasing Agents, Farm Products	0.973	0.370	0.335 (0)
247	First-Line Supervisors of Personal Service Workers	0.973	0.913	0.090 ($< 10^{-142}$)
248	Amusement and Recreation Attendants	0.973	0.979	0.232 (0)
249	Coaches and Scouts	0.973	0.876	-0.014 ($< 10^{-2}$)
250	Self-Enrichment Education Teachers	0.973	0.948	-0.213 (0)
251	Medical and Clinical Laboratory Technologists	0.973	0.905	0.237 (0)
252	Pharmacy Technicians	0.973	0.882	0.310 (0)
253	Food Scientists and Technologists	0.973	0.373	0.268 ($< 10^{-216}$)
254	Painting, Coating, and Decorating Workers	0.973	0.570	0.519 (0)
255	Sales Engineers	0.973	1.004	-0.442 (0)
256	Food Servers, Nonrestaurant	0.973	0.912	0.064 ($< 10^{-60}$)
257	Geoscientists, Except Hydrologists and Geographers	0.974	0.515	0.320 (0)
258	Electronics Engineers, Except Computer	0.974	1.002	-0.350 (0)
259	Optometrists	0.974	0.813	0.175 ($< 10^{-216}$)
260	Occupational Therapy Assistants	0.974	0.583	0.323 (0)
261	Personal Care Aides	0.974	0.877	0.100 ($< 10^{-169}$)
262	Postal Service Clerks	0.974	0.814	0.219 (0)
263	Painters, Transportation Equipment	0.974	0.701	0.350 (0)
264	Food Batchmakers	0.974	0.663	0.311 (0)
265	Welding, Soldering, and Brazing Machine Setters, Operators, and Tenders	0.974	0.443	0.240 (0)
266	Dining Room and Cafeteria Attendants and Bartender Helpers	0.974	1.019	0.139 ($< 10^{-312}$)
267	Environmental Engineers	0.974	0.780	-0.154 ($< 10^{-199}$)
268	Industrial Engineers	0.974	0.899	0.080 ($< 10^{-94}$)
269	Electrical Engineers	0.974	1.020	-0.403 (0)
270	Industrial Production Managers	0.974	0.840	0.256 (0)
271	Pressers, Textile, Garment, and Related Materials	0.974	0.749	0.351 (0)
272	Materials Engineers	0.974	0.679	0.037 ($< 10^{-5}$)
273	Extruding, Forming, Pressing, and Compacting Machine Setters, Operators, and Tenders	0.974	0.540	0.329 (0)
274	Plating and Coating Machine Setters, Operators, and Tenders, Metal and Plastic	0.974	0.487	0.382 (0)
275	Cooks, Institution and Cafeteria	0.974	0.776	0.290 (0)
276	Bartenders	0.974	0.932	0.118 ($< 10^{-253}$)
277	General and Operations Managers	0.974	1.024	-0.269 (0)
278	Dispatchers, Except Police, Fire, and Ambulance	0.974	0.991	0.225 (0)
279	Cutting, Punching, and Press Machine Setters, Operators, and Tenders, Metal and Plastic	0.974	0.712	0.355 (0)
280	Licensed Practical and Licensed Vocational Nurses	0.974	0.845	0.293 (0)
281	First-Line Supervisors of Food Preparation and Serving Workers	0.974	0.881	0.363 (0)
282	Tool and Die Makers	0.974	0.531	0.256 (0)
283	Landscape Architects	0.974	0.680	0.087 ($< 10^{-21}$)
284	Lodging Managers	0.975	0.593	0.397 (0)

285	Physician Assistants	0.975	0.816	0.031 ($< 10^{-12}$)
286	Architects, Except Landscape and Naval	0.975	1.034	-0.288 (0)
287	Counter and Rental Clerks	0.975	0.933	0.215 (0)
288	Baggage Porters and Bellhops	0.975	0.783	0.439 (0)
289	Food Cooking Machine Operators and Tenders	0.975	0.359	0.486 (0)
290	Computer, Automated Teller, and Office Machine Repairers	0.975	0.982	0.028 ($< 10^{-9}$)
291	Cashiers	0.975	0.854	0.491 (0)
292	Electrical and Electronics Engineering Technicians	0.975	0.958	-0.310 (0)
293	Zoologists and Wildlife Biologists	0.975	0.299	0.147 ($< 10^{-82}$)
294	Nonfarm Animal Caretakers	0.975	0.960	0.048 ($< 10^{-37}$)
295	First-Line Supervisors of Transportation and Material-Moving Machine and Vehicle Operators	0.975	0.949	0.319 (0)
296	Dental Hygienists	0.975	0.898	0.103 ($< 10^{-184}$)
297	Dental Assistants	0.975	0.920	0.017 ($< 10^{-5}$)
298	Ophthalmic Laboratory Technicians	0.975	0.632	0.152 ($< 10^{-95}$)
299	Retail Salespersons	0.975	0.904	0.456 (0)
300	Chiropractors	0.975	0.727	0.295 (0)
301	Coin, Vending, and Amusement Machine Servicers and Repairers	0.975	0.635	0.400 (0)
302	Upholsterers	0.975	0.459	0.345 (0)
303	Motorcycle Mechanics	0.975	0.429	0.490 (0)
304	Hazardous Materials Removal Workers	0.975	0.712	0.319 (0)
305	Grinding, Lapping, Polishing, and Buffing Machine Tool Setters, Operators, and Tenders, Metal and Plastic	0.975	0.624	0.335 (0)
306	Dentists, General	0.975	0.900	0.074 ($< 10^{-73}$)
307	Photographers	0.975	0.929	0.203 (0)
308	Jewelers and Precious Stone and Metal Workers	0.975	0.743	0.187 ($< 10^{-108}$)
309	Maintenance Workers, Machinery	0.975	0.685	0.412 (0)
310	Stationary Engineers and Boiler Operators	0.975	0.572	0.205 ($< 10^{-253}$)
311	Cabinetmakers and Bench Carpenters	0.976	0.726	0.329 (0)
312	Medical Equipment Preparers	0.976	0.766	0.182 ($< 10^{-291}$)
313	Coating, Painting, and Spraying Machine Setters, Operators, and Tenders	0.976	0.657	0.398 (0)
314	Butchers and Meat Cutters	0.976	0.844	0.307 (0)
315	Driver/Sales Workers	0.976	0.943	0.204 (0)
316	Aircraft Mechanics and Service Technicians	0.976	0.812	-0.034 ($< 10^{-9}$)
317	Glaziers	0.976	0.728	0.249 ($< 10^{-305}$)
318	Home Health Aides	0.976	0.937	0.018 ($< 10^{-4}$)
319	Power Plant Operators	0.976	0.549	0.364 (0)
320	Electromechanical Equipment Assemblers	0.976	0.625	0.103 ($< 10^{-41}$)
321	Broadcast Technicians	0.976	0.713	0.309 (0)
322	Prepress Technicians and Workers	0.976	0.742	0.199 ($< 10^{-280}$)
323	Tile and Marble Setters	0.976	0.646	0.378 (0)
324	Excavating and Loading Machine and Dragline Operators	0.976	0.423	0.335 (0)
325	Veterinarians	0.976	0.813	0.132 ($< 10^{-228}$)
326	Shipping, Receiving, and Traffic Clerks	0.976	1.026	0.181 (0)
327	Cooks, Fast Food	0.976	0.763	0.395 (0)
328	Logging Equipment Operators	0.976	0.104	0.150 ($< 10^{-65}$)
329	Cardiovascular Technologists and Technicians	0.976	0.807	0.350 (0)
330	Packaging and Filling Machine Operators and Tenders	0.976	0.805	0.273 (0)

331	Helpers–Brickmasons, Blockmasons, Stonemasons, and Tile and Marble Setters	0.976	0.510	0.374 (0)
332	Childcare Workers	0.976	0.945	0.003 ($< 10^{0}$)
333	Rolling Machine Setters, Operators, and Tenders, Metal and Plastic	0.976	0.422	0.446 (0)
334	Home Appliance Repairers	0.976	0.766	0.426 (0)
335	Helpers–Pipelayers, Plumbers, Pipefitters, and Steamfitters	0.976	0.640	0.247 (0)
336	Packers and Packagers, Hand	0.976	0.969	0.176 (0)
337	Farm Equipment Mechanics and Service Technicians	0.976	0.164	0.425 (0)
338	Emergency Management Directors	0.976	0.385	0.265 ($< 10^{-182}$)
339	Computer Numerically Controlled Machine Tool Programmers, Metal and Plastic	0.976	0.534	0.236 ($< 10^{-300}$)
340	Opticians, Dispensing	0.976	0.798	0.319 (0)
341	Laborers and Freight, Stock, and Material Movers, Hand	0.976	1.015	0.262 (0)
342	Crane and Tower Operators	0.976	0.509	0.323 (0)
343	Animal Control Workers	0.977	0.491	0.296 (0)
344	Grinding and Polishing Workers, Hand	0.977	0.499	0.392 (0)
345	Food Preparation Workers	0.977	0.914	0.219 (0)
346	Meter Readers, Utilities	0.977	0.605	0.320 (0)
347	Pipelayers	0.977	0.595	0.471 (0)
348	Audio and Video Equipment Technicians	0.977	0.974	0.072 ($< 10^{-36}$)
349	Police and Sheriff's Patrol Officers	0.977	0.918	0.137 (0)
350	First-Line Supervisors of Retail Sales Workers	0.977	0.868	0.479 (0)
351	Helpers–Installation, Maintenance, and Repair Workers	0.977	0.855	0.344 (0)
352	Occupational Health and Safety Specialists	0.977	0.786	0.077 ($< 10^{-71}$)
353	Medical and Clinical Laboratory Technicians	0.977	0.953	0.027 ($< 10^{-9}$)
354	Correctional Officers and Jailers	0.977	0.743	0.102 ($< 10^{-107}$)
355	Drywall and Ceiling Tile Installers	0.977	0.801	0.198 ($< 10^{-309}$)
356	Engineering Technicians, Except Drafters, All Other	0.977	0.724	-0.013 ($< 10^{0}$)
357	Cooks, Short Order	0.977	0.759	0.078 ($< 10^{-59}$)
358	Athletic Trainers	0.977	0.662	0.145 ($< 10^{-120}$)
359	Veterinary Technologists and Technicians	0.977	0.843	0.042 ($< 10^{-21}$)
360	Taxi Drivers and Chauffeurs	0.977	0.958	-0.014 ($< 10^{-2}$)
361	Detectives and Criminal Investigators	0.977	0.832	0.109 ($< 10^{-150}$)
362	Agricultural and Food Science Technicians	0.977	0.245	0.353 (0)
363	Physical Therapist Aides	0.977	0.702	0.294 (0)
364	Life, Physical, and Social Science Technicians, All Other	0.977	0.780	-0.109 ($< 10^{-92}$)
365	Veterinary Assistants and Laboratory Animal Caretakers	0.977	0.764	0.103 ($< 10^{-108}$)
366	First-Line Supervisors of Production and Operating Workers	0.977	0.856	0.350 (0)
367	Structural Metal Fabricators and Fitters	0.977	0.660	0.378 (0)
368	Drilling and Boring Machine Tool Setters, Operators, and Tenders, Metal and Plastic	0.977	0.482	0.576 (0)
369	Construction and Building Inspectors	0.977	0.875	0.081 ($< 10^{-88}$)
370	Nuclear Medicine Technologists	0.977	0.685	0.332 (0)
371	Electrical Power-Line Installers and Repairers	0.977	0.723	0.247 (0)
372	Dental Laboratory Technicians	0.977	0.722	0.298 (0)
373	Medical Equipment Repairers	0.977	0.794	0.207 ($< 10^{-322}$)
374	Physical Therapist Assistants	0.977	0.742	0.361 (0)
375	Locksmiths and Safe Repairers	0.977	0.669	0.406 (0)
376	Respiratory Therapists	0.977	0.865	0.282 (0)
377	Security and Fire Alarm Systems Installers	0.977	0.885	0.178 ($< 10^{-230}$)

378	Machine Feeders and Offbearers	0.977	0.628	0.281 (0)
379	Bakers	0.977	0.924	0.168 (0)
380	Parking Lot Attendants	0.977	1.174	0.063 ($< 10^{-29}$)
381	Recreation Workers	0.978	0.940	0.003 ($< 10^{0}$)
382	Landscaping and Groundskeeping Workers	0.978	0.972	0.163 (0)
383	Career/Technical Education Teachers, Secondary School	0.978	0.657	0.367 (0)
384	Surveyors	0.978	0.690	0.304 (0)
385	Office Machine Operators, Except Computer	0.978	0.944	-0.002 ($< 10^{0}$)
386	Commercial Pilots	0.978	0.586	0.249 (0)
387	Engine and Other Machine Assemblers	0.978	0.322	0.109 ($< 10^{-30}$)
388	Bus Drivers, Transit and Intercity	0.978	0.938	0.034 ($< 10^{-7}$)
389	Industrial Truck and Tractor Operators	0.978	0.917	0.345 (0)
390	Pesticide Handlers, Sprayers, and Applicators, Vegetation	0.978	0.293	0.523 (0)
391	Roofers	0.978	0.795	0.382 (0)
392	Motorboat Mechanics and Service Technicians	0.978	0.380	0.490 (0)
393	Light Truck or Delivery Services Drivers	0.978	0.964	0.174 (0)
394	Operating Engineers and Other Construction Equipment Operators	0.978	0.853	0.221 (0)
395	Mixing and Blending Machine Setters, Operators, and Tenders	0.978	0.747	0.317 (0)
396	Carpet Installers	0.978	0.644	0.244 ($< 10^{-162}$)
397	Industrial Machinery Mechanics	0.978	0.773	0.382 (0)
398	Brickmasons and Blockmasons	0.978	0.775	0.266 (0)
399	Surveying and Mapping Technicians	0.978	0.688	0.262 (0)
400	Agricultural Inspectors	0.978	0.137	0.520 (0)
401	First-Line Supervisors of Housekeeping and Janitorial Workers	0.978	0.938	0.088 ($< 10^{-140}$)
402	Janitors and Cleaners, Except Maids and Housekeeping Cleaners	0.978	0.940	0.114 ($< 10^{-247}$)
403	Welders, Cutters, Solderers, and Brazers	0.978	0.812	0.247 (0)
404	First-Line Supervisors of Police and Detectives	0.978	0.791	0.114 ($< 10^{-201}$)
405	Animal Trainers	0.978	0.437	0.420 (0)
406	Radiation Therapists	0.978	0.610	0.372 (0)
407	First-Line Supervisors of Landscaping, Lawn Service, and Groundskeeping Workers	0.978	0.902	0.226 (0)
408	Computer-Controlled Machine Tool Operators, Metal and Plastic	0.978	0.649	0.281 (0)
409	Fire Inspectors and Investigators	0.979	0.468	0.207 ($< 10^{-145}$)
410	Surgical Technologists	0.979	0.796	0.350 (0)
411	Sheet Metal Workers	0.979	0.870	0.167 (0)
412	Camera Operators, Television, Video, and Motion Picture	0.979	0.860	0.126 ($< 10^{-60}$)
413	Electrical and Electronics Repairers, Powerhouse, Substation, and Relay	0.979	0.448	0.204 ($< 10^{-125}$)
414	Environmental Science and Protection Technicians, Including Health	0.979	0.717	0.206 ($< 10^{-315}$)
415	Pest Control Workers	0.979	0.734	0.435 (0)
416	File Clerks	0.979	0.965	0.087 ($< 10^{-114}$)
417	Combined Food Preparation and Serving Workers, Including Fast Food	0.979	0.909	0.281 (0)
418	Conservation Scientists	0.979	0.255	0.092 ($< 10^{-37}$)
419	Millwrights	0.979	0.466	0.295 (0)
420	Dietetic Technicians	0.979	0.574	0.311 (0)
421	Structural Iron and Steel Workers	0.979	0.713	0.339 (0)
422	Mechanical Engineering Technicians	0.979	0.751	0.037 ($< 10^{-9}$)
423	Molders, Shapers, and Casters, Except Metal and Plastic	0.979	0.558	0.386 (0)
424	Conveyor Operators and Tenders	0.979	0.446	0.476 (0)
425	Transportation Inspectors	0.979	0.634	0.130 ($< 10^{-55}$)

426	Forensic Science Technicians	0.979	0.498	0.156 ($< 10^{-83}$)
427	Diagnostic Medical Sonographers	0.979	0.817	0.198 (0)
428	Cleaners of Vehicles and Equipment	0.979	0.930	0.313 (0)
429	Cement Masons and Concrete Finishers	0.979	0.847	0.277 (0)
430	Machinists	0.979	0.877	0.194 (0)
431	Dishwashers	0.979	0.945	0.133 (0)
432	Multiple Machine Tool Setters, Operators, and Tenders, Metal and Plastic	0.979	0.460	0.317 (0)
433	Chefs and Head Cooks	0.979	0.931	0.072 ($< 10^{-65}$)
434	Outdoor Power Equipment and Other Small Engine Mechanics	0.979	0.532	0.532 (0)
435	Insulation Workers, Mechanical	0.979	0.414	0.326 (0)
436	Heavy and Tractor-Trailer Truck Drivers	0.979	0.901	0.380 (0)
437	Electric Motor, Power Tool, and Related Repairers	0.979	0.479	0.443 (0)
438	Food Service Managers	0.980	0.906	0.048 ($< 10^{-41}$)
439	Automotive and Watercraft Service Attendants	0.980	0.762	0.155 ($< 10^{-280}$)
440	Refuse and Recyclable Material Collectors	0.980	0.763	0.329 (0)
441	Automotive Service Technicians and Mechanics	0.980	0.888	0.324 (0)
442	Electrical and Electronics Repairers, Commercial and Industrial Equipment	0.980	0.715	-0.076 ($< 10^{-57}$)
443	Occupational Health and Safety Technicians	0.980	0.420	0.436 (0)
444	Bus and Truck Mechanics and Diesel Engine Specialists	0.980	0.895	0.286 (0)
445	Paving, Surfacing, and Tamping Equipment Operators	0.980	0.635	0.376 (0)
446	Water and Wastewater Treatment Plant and System Operators	0.980	0.721	0.312 (0)
447	First-Line Supervisors of Construction Trades and Extraction Workers	0.980	0.955	0.155 (0)
448	Telecommunications Line Installers and Repairers	0.980	0.854	0.256 (0)
449	Laundry and Dry-Cleaning Workers	0.980	0.921	0.239 (0)
450	Chemical Plant and System Operators	0.980	0.099	0.412 (0)
451	Environmental Engineering Technicians	0.981	0.589	0.287 (0)
452	Crushing, Grinding, and Polishing Machine Setters, Operators, and Tenders	0.981	0.435	0.425 (0)
453	Mobile Heavy Equipment Mechanics, Except Engines	0.981	0.780	0.203 (0)
454	Cleaning, Washing, and Metal Pickling Equipment Operators and Tenders	0.981	0.349	0.255 ($< 10^{-234}$)
455	Heating, Air Conditioning, and Refrigeration Mechanics and Installers	0.981	0.916	0.208 (0)
456	Separating, Filtering, Clarifying, Precipitating, and Still Machine Setters, Operators, and Tenders	0.981	0.366	0.312 (0)
457	Plumbers, Pipefitters, and Steamfitters	0.981	0.960	0.037 ($< 10^{-24}$)
458	Heat Treating Equipment Setters, Operators, and Tenders, Metal and Plastic	0.981	0.415	0.366 (0)
459	Helpers–Carpenters	0.981	0.576	0.297 (0)
460	Tire Repairers and Changers	0.981	0.698	0.451 (0)
461	Electricians	0.981	0.964	0.062 ($< 10^{-70}$)
462	Automotive Body and Related Repairers	0.981	0.890	0.293 (0)
463	Highway Maintenance Workers	0.981	0.587	0.212 (0)
464	Electronic Home Entertainment Equipment Installers and Repairers	0.981	0.571	0.401 (0)
465	Meat, Poultry, and Fish Cutters and Trimmers	0.981	0.528	0.352 (0)
466	Cooks, Restaurant	0.982	0.964	0.197 (0)
467	Firefighters	0.982	0.851	0.207 (0)
468	Control and Valve Installers and Repairers, Except Mechanical Door	0.982	0.584	0.415 (0)
469	Painters, Construction and Maintenance	0.982	0.969	0.054 ($< 10^{-49}$)
470	Maintenance and Repair Workers, General	0.982	0.908	0.364 (0)

471	First-Line Supervisors of Mechanics, Installers, and Repairers	0.982	0.911	0.181 (0)
472	Telecommunications Equipment Installers and Repairers, Except Line Installers	0.982	0.963	0.062 ($< 10^{-55}$)
473	Septic Tank Servicers and Sewer Pipe Cleaners	0.982	0.522	0.475 (0)
474	First-Line Supervisors of Farming, Fishing, and Forestry Workers	0.982	0.035	0.321 (0)
475	First-Line Supervisors of Fire Fighting and Prevention Workers	0.982	0.734	0.205 (0)
476	Carpenters	0.983	0.957	0.115 ($< 10^{-250}$)
477	Emergency Medical Technicians and Paramedics	0.983	0.807	0.217 (0)
478	Construction Laborers	0.983	0.943	0.230 (0)
479	Extruding and Drawing Machine Setters, Operators, and Tenders, Metal and Plastic	0.984	0.463	0.429 (0)
480	Insulation Workers, Floor, Ceiling, and Wall	0.985	0.499	0.289 ($< 10^{-218}$)
481	Forest and Conservation Technicians	0.985	0.058	0.376 (0)

6.3 Job Groups

The O*NET skills database allows us to identify how important each of 230 workplace skills is to completing each of the BLS jobs. We use K-means clustering to group jobs into five groups according to the skills required to perform those jobs. The complete list of BLS jobs comprising each job group is presented in the table below. Our interpretation about the scaling behaviors of jobs, and how aggregate skills indicate those scaling behaviors, is the same if we use anywhere between three and seven job groups instead of five while computing the K-means clustering algorithm.

Group (β)	BLS Jobs
Purple (1.39)	Aerospace Engineers, Agricultural Engineers, Animal Scientists, Anthropologists and Archeologists, Architects, Except Landscape and Naval, Architectural and Civil Drafters, Architectural and Engineering Managers, Astronomers, Atmospheric and Space Scientists, Biochemists and Biophysicists, Biological Scientists, All Other, Biomedical Engineers, Cartographers and Photogrammetrists, Chemical Engineers, Chemists, Civil Engineering Technicians, Civil Engineers, Commercial and Industrial Designers, Computer Hardware Engineers, Computer Programmers, Computer Systems Analysts, Computer and Information Research Scientists, Computer and Information Systems Managers, Construction Managers, Database Administrators, Electrical Engineers, Electrical and Electronics Drafters, Electronics Engineers, Except Computer, Engineers, All Other, Environmental Engineers, Environmental Scientists and Specialists, Including Health, Food Scientists and Technologists, Geographers, Geoscientists, Except Hydrologists and Geographers, Health and Safety Engineers, Except Mining Safety Engineers and Inspectors, Hydrologists, Industrial Engineering Technicians, Industrial Engineers, Landscape Architects, Logisticians, Marine Engineers and Naval Architects, Materials Engineers, Materials Scientists, Mathematical Technicians, Mathematicians, Mechanical Drafters, Mechanical Engineers, Medical Scientists, Except Epidemiologists, Microbiologists, Mining and Geological Engineers, Including Mining Safety Engineers, Multimedia Artists and Animators, Natural Sciences Managers, Network and Computer Systems Administrators, Nuclear Engineers, Occupational Health and Safety Specialists, Operations Research Analysts, Petroleum Engineers, Physical Scientists, All Other, Physicists, Sales Engineers, Set and Exhibit Designers, Social Scientists and Related Workers, All Other, Software Developers, Applications, Software Developers, Systems Software, Soil and Plant Scientists, Statistical Assistants, Statisticians, Technical Writers

Green (1.08)	Accountants and Auditors, Actuaries, Administrative Law Judges, Adjudicators, and Hearing Officers, Administrative Services Managers, Adult Basic and Secondary Education and Literacy Teachers and Instructors, Advertising Sales Agents, Advertising and Promotions Managers, Agents and Business Managers of Artists, Performers, and Athletes, Air Traffic Controllers, Appraisers and Assessors of Real Estate, Arbitrators, Mediators, and Conciliators, Archivists, Art Directors, Audiologists, Broadcast News Analysts, Budget Analysts, Business Operations Specialists, All Other, Buyers and Purchasing Agents, Farm Products, Career/Technical Education Teachers, Middle School, Cargo and Freight Agents, Chief Executives, Child, Family, and School Social Workers, Choreographers, Claims Adjusters, Examiners, and Investigators, Clergy, Clinical, Counseling, and School Psychologists, Coaches and Scouts, Compensation and Benefits Managers, Compensation, Benefits, and Job Analysis Specialists, Compliance Officers, Concierges, Cost Estimators, Credit Analysts, Credit Authorizers, Checkers, and Clerks, Credit Counselors, Curators, Customer Service Representatives, Dietitians and Nutritionists, Directors, Religious Activities and Education, Dispatchers, Except Police, Fire, and Ambulance, Economists, Editors, Education Administrators, Elementary and Secondary School, Education Administrators, Postsecondary, Education Administrators, Preschool and Childcare Center/Program, Educational, Guidance, School, and Vocational Counselors, Elementary School Teachers, Except Special Education, Eligibility Interviewers, Government Programs, Emergency Management Directors, Epidemiologists, Executive Secretaries and Executive Administrative Assistants, Farm and Home Management Advisors, Fashion Designers, Film and Video Editors, Financial Analysts, Financial Examiners, Financial Managers, Financial Specialists, All Other, First-Line Supervisors of Non-Retail Sales Workers, First-Line Supervisors of Office and Administrative Support Workers, First-Line Supervisors of Personal Service Workers, First-Line Supervisors of Transportation and Material-Moving Machine and Vehicle Operators, Food Service Managers, Gaming Managers, Gaming Supervisors, General and Operations Managers, Graphic Designers, Health Diagnosing and Treating Practitioners, All Other, Health Educators, Healthcare Social Workers, Historians, Human Resources Assistants, Except Payroll and Timekeeping, Human Resources Managers, Industrial-Organizational Psychologists, Instructional Coordinators, Insurance Sales Agents, Insurance Underwriters, Interior Designers, Interpreters and Translators, Judges, Magistrate Judges, and Magistrates, Judicial Law Clerks, Kindergarten Teachers, Except Special Education, Lawyers, Librarians, Loan Interviewers and Clerks, Loan Officers, Lodging Managers, Management Analysts, Managers, All Other, Market Research Analysts and Marketing Specialists, Marketing Managers, Marriage and Family Therapists, Medical Assistants, Medical and Health Services Managers, Meeting, Convention, and Event Planners, Mental Health Counselors, Mental Health and Substance Abuse Social Workers, Middle School Teachers, Except Special and Career/Technical Education, Music Directors and Composers, New Accounts Clerks, Occupational Therapists, Opticians, Dispensing, Paralegals and Legal Assistants, Personal Financial Advisors, Pharmacists, Police, Fire, and Ambulance Dispatchers, Political Scientists, Postmasters and Mail Superintendents, Preschool Teachers, Except Special Education, Private Detectives and Investigators, Probation Officers and Correctional Treatment Specialists, Procurement Clerks, Producers and Directors, Production, Planning, and Expediting Clerks, Property, Real Estate, and Community Association Managers, Psychiatric Technicians, Psychologists, All Other, Public Relations Specialists, Public Relations and Fundraising Managers, Purchasing Agents, Except Wholesale, Retail, and Farm Products, Purchasing Managers, Radio and Television Announcers, Real Estate Brokers, Real Estate Sales Agents, Recreation Workers, Recreational Therapists, Rehabilitation Counselors, Reporters and Correspondents, Residential Advisors, Sales Managers, Sales Representatives, Wholesale and Manufacturing, Except Technical and Scientific Products, Sales Representatives, Wholesale and Manufacturing, Technical and Scientific Products, Secondary School Teachers, Except Special and Career/Technical Education, Securities, Commodities, and Financial Services Sales Agents, Social Science Research Assistants, Social and Community Service Managers, Social and Human Service Assistants, Sociologists, Special Education Teachers, Middle School, Special Education Teachers, Secondary School, Speech-Language Pathologists, Substance Abuse and Behavioral Disorder Counselors, Survey Researchers, Tax Examiners and Collectors, and Revenue Agents, Tax Preparers, Training and Development Managers, Training and Development Specialists, Transportation, Storage, and Distribution Managers, Travel Agents, Travel Guides, Urban and Regional Planners, Wholesale and Retail Buyers, Except Farm Products, Writers and Authors
Yellow (1.02)	Aerospace Engineering and Operations Technicians, Agricultural Inspectors, Agricultural and Food Science Technicians, Aircraft Cargo Handling Supervisors, Aircraft Mechanics and Service Technicians, Airfield Operations Specialists, Airline Pilots, Copilots, and Flight Engineers, Animal Control Workers, Animal Trainers, Athletic Trainers, Audio and Video Equipment Technicians, Audio-Visual and Multimedia Collections Specialists, Avionics Technicians, Biological Technicians, Broadcast Technicians, Captains, Mates, and Pilots of Water Vessels, Cardiovascular Technologists and Technicians, Career/Technical Education Teachers, Secondary School, Chefs and Head Cooks, Chemical Technicians, Chiropractors, Commercial Divers, Commercial Pilots, Computer Numerically Controlled Machine Tool Programmers, Metal and Plastic, Computer Operators, Computer, Automated Teller, and Office Machine Repairers, Conservation Scientists, Construction and Building Inspectors, Correctional Officers and Jailers, Dental Assistants, Dental Hygienists, Dental Laboratory Technicians, Dentists, General, Desktop Publishers, Detectives and Criminal Investigators, Diagnostic Medical Sonographers, Dietetic Technicians, Electrical Power-Line Installers and Repairers, Electrical and Electronics Engineering Technicians, Electrical and Electronics Repairers, Commercial and Industrial Equipment, Electrical and Electronics Repairers, Powerhouse, Substation, and Relay, Electricians, Electro-Mechanical Technicians, Electronic Equipment Installers and Repairers, Motor Vehicles, Electronic Home Entertainment Equipment Installers and Repairers, Elevator Installers and Repairers, Embalmers, Emergency Medical Technicians and Paramedics, Engineering Technicians, Except Drafters, All Other, Environmental Engineering Technicians, Environmental Science and Protection Technicians, Including Health, Explosives Workers, Ordnance Handling Experts, and Blasters, Fabric and Apparel Patternmakers, Farmers, Ranchers, and Other Agricultural Managers, Fire Inspectors and Investigators, Firefighters, First-Line Supervisors of Construction Trades and Extraction Workers, First-Line Supervisors of Correctional Officers, First-Line Supervisors of Farming, Fishing, and Forestry Workers, First-Line Supervisors of Fire Fighting and Prevention Workers, First-Line Supervisors of Landscaping, Lawn Service, and Groundskeeping Workers, First-Line Supervisors of Mechanics, Installers, and Repairers, First-Line Supervisors of Police and Detectives, First-Line Supervisors of Production and Operating Workers, Fish and Game Wardens, Forensic Science Technicians, Forest Fire Inspectors and Prevention Specialists, Forest and Conservation Technicians, Forest and Conservation Workers, Foresters, Gaming Surveillance Officers and Gaming Investigators, Geological and Petroleum Technicians, Hazardous Materials Removal Workers, Heating, Air Conditioning, and Refrigeration Mechanics and Installers, Industrial Production Managers, Licensed Practical and Licensed Vocational Nurses, Life, Physical, and Social Science Technicians, All Other, Manufactured Building and Mobile Home Installers, Mechanical Engineering Technicians, Medical Appliance Technicians, Medical Equipment Preparers, Medical Equipment Repairers, Medical and Clinical Laboratory Technicians, Medical and Clinical Laboratory Technologists, Museum Technicians and Conservators, Nuclear Medicine Technologists, Nuclear Power Reactor Operators, Nuclear Technicians, Occupational Health and Safety Technicians, Occupational Therapy Assistants, Optometrists, Oral and Maxillofacial Surgeons, Orthodontists, Orthotists and Prosthetists, Pest Control Workers, Photographers, Physical Therapist Assistants, Physical Therapists, Physician Assistants, Podiatrists, Police and Sheriff's Patrol Officers, Power Distributors and Dispatchers, Radiation Therapists, Radio, Cellular, and Tower Equipment Installers and Repairers, Railroad Conductors and Yardmasters, Respiratory Therapists, Respiratory Therapy Technicians, Service Unit Operators, Oil, Gas, and Mining, Ship Engineers, Sound Engineering Technicians, Stationary Engineers and Boiler Operators, Surgical Technologists, Surveying and Mapping Technicians, Surveyors, Telecommunications Equipment Installers and Repairers, Except Line Installers, Traffic Technicians, Transit and Railroad Police, Transportation Attendants, Except Flight Attendants, Transportation Inspectors, Veterinarians, Veterinary Assistants and Laboratory Animal Caretakers, Veterinary Technologists and Technicians, Water and Wastewater Treatment Plant and System Operators, Zoologists and Wildlife Biologists

Red (0.98)	Adhesive Bonding Machine Operators and Tenders, Aircraft Structure, Surfaces, Rigging, and Systems Assemblers, Ambulance Drivers and Attendants, Except Emergency Medical Technicians, Animal Breeders, Automotive Body and Related Repairers, Automotive Glass Installers and Repairers, Automotive Service Technicians and Mechanics, Automotive and Watercraft Service Attendants, Bakers, Bicycle Repairers, Boilermakers, Brickmasons and Blockmasons, Bridge and Lock Tenders, Bus Drivers, School or Special Client, Bus Drivers, Transit and Intercity, Bus and Truck Mechanics and Diesel Engine Specialists, Butchers and Meat Cutters, Cabinetmakers and Bench Carpenters, Camera Operators, Television, Video, and Motion Picture, Camera and Photographic Equipment Repairers, Carpenters, Carpet Installers, Cement Masons and Concrete Finishers, Chemical Equipment Operators and Tenders, Chemical Plant and System Operators, Cleaners of Vehicles and Equipment, Cleaning, Washing, and Metal Pickling Equipment Operators and Tenders, Coating, Painting, and Spraying Machine Setters, Operators, and Tenders, Coil Winders, Tapers, and Finishers, Coin, Vending, and Amusement Machine Servicers and Repairers, Computer-Controlled Machine Tool Operators, Metal and Plastic, Construction Laborers, Continuous Mining Machine Operators, Control and Valve Installers and Repairers, Except Mechanical Door, Conveyor Operators and Tenders, Cooks, Restaurant, Cooling and Freezing Equipment Operators and Tenders, Couriers and Messengers, Craft Artists, Crane and Tower Operators, Crushing, Grinding, and Polishing Machine Setters, Operators, and Tenders, Cutters and Trimmers, Hand, Cutting and Slicing Machine Setters, Operators, and Tenders, Cutting, Punching, and Press Machine Setters, Operators, and Tenders, Metal and Plastic, Derrick Operators, Oil and Gas, Dishwashers, Drilling and Boring Machine Tool Setters, Operators, and Tenders, Metal and Plastic, Drywall and Ceiling Tile Installers, Earth Drillers, Except Oil and Gas, Electric Motor, Power Tool, and Related Repairers, Electrical and Electronic Equipment Assemblers, Electrical and Electronics Installers and Repairers, Transportation Equipment, Electromechanical Equipment Assemblers, Engine and Other Machine Assemblers, Etchers and Engravers, Excavating and Loading Machine and Dragline Operators, Extruding and Drawing Machine Setters, Operators, and Tenders, Metal and Plastic, Extruding and Forming Machine Setters, Operators, and Tenders, Synthetic and Glass Fibers, Extruding, Forming, Pressing, and Compacting Machine Setters, Operators, and Tenders, Fabric Menders, Except Garment, Fallers, Farm Equipment Mechanics and Service Technicians, Fence Erectors, Fiberglass Laminators and Fabricators, Fishers and Related Fishing Workers, Floor Layers, Except Carpet, Wood, and Hard Tiles, Floor Sanders and Finishers, Food Batchmakers, Food Cooking Machine Operators and Tenders, Food and Tobacco Roasting, Baking, and Drying Machine Operators and Tenders, Forging Machine Setters, Operators, and Tenders, Metal and Plastic, Foundry Mold and Coremakers, Furnace, Kiln, Oven, Drier, and Kettle Operators and Tenders, Furniture Finishers, Gas Compressor and Gas Pumping Station Operators, Gas Plant Operators, Glaziers, Grinding and Polishing Workers, Hand, Grinding, Lapping, Polishing, and Buffing Machine Tool Setters, Operators, and Tenders, Metal and Plastic, Heat Treating Equipment Setters, Operators, and Tenders, Metal and Plastic, Heavy and Tractor-Trailer Truck Drivers, Helpers–Brickmasons, Blockmasons, Stonemasons, and Tile and Marble Setters, Helpers–Carpenters, Helpers–Electricians, Helpers–Extraction Workers, Helpers–Installation, Maintenance, and Repair Workers, Helpers–Painters, Paperhangers, Plasterers, and Stucco Masons, Helpers–Pipelayers, Plumbers, Pipefitters, and Steamfitters, Helpers–Production Workers, Helpers–Roofers, Highway Maintenance Workers, Hoist and Winch Operators, Home Appliance Repairers, Industrial Machinery Mechanics, Industrial Truck and Tractor Operators, Insulation Workers, Floor, Ceiling, and Wall, Insulation Workers, Mechanical, Janitors and Cleaners, Except Maids and Housekeeping Cleaners, Jewelers and Precious Stone and Metal Workers, Laborers and Freight, Stock, and Material Movers, Hand, Landscaping and Groundskeeping Workers, Lathe and Turning Machine Tool Setters, Operators, and Tenders, Metal and Plastic, Laundry and Dry-Cleaning Workers, Layout Workers, Metal and Plastic, Light Truck or Delivery Services Drivers, Locksmiths and Safe Repairers, Locomotive Engineers, Log Graders and Scalers, Logging Equipment Operators, Machine Feeders and Offbearers, Machinists, Maintenance Workers, Machinery, Maintenance and Repair Workers, General, Meat, Poultry, and Fish Cutters and Trimmers, Mechanical Door Repairers, Metal-Refining Furnace Operators and Tenders, Meter Readers, Utilities, Milling and Planing Machine Setters, Operators, and Tenders, Metal and Plastic, Millwrights, Mine Cutting and Channeling Machine Operators, Mine Shuttle Car Operators, Mixing and Blending Machine Setters, Operators, and Tenders, Mobile Heavy Equipment Mechanics, Except Engines, Model Makers, Metal and Plastic, Model Makers, Wood, Molders, Shapers, and Casters, Except Metal and Plastic, Molding, Coremaking, and Casting Machine Setters, Operators, and Tenders, Metal and Plastic, Motion Picture Projectionists, Motorboat Mechanics and Service Technicians, Motorboat Operators, Motorcycle Mechanics, Multiple Machine Tool Setters, Operators, and Tenders, Metal and Plastic, Musical Instrument Repairers and Tuners, Office Machine Operators, Except Computer, Operating Engineers and Other Construction Equipment Operators, Ophthalmic Laboratory Technicians, Outdoor Power Equipment and Other Small Engine Mechanics, Packaging and Filling Machine Operators and Tenders, Packers and Packagers, Hand, Painters, Construction and Maintenance, Painters, Transportation Equipment, Painting, Coating, and Decorating Workers, Paper Goods Machine Setters, Operators, and Tenders, Paperhangers, Parking Lot Attendants, Patternmakers, Metal and Plastic, Patternmakers, Wood, Paving, Surfacing, and Tamping Equipment Operators, Pesticide Handlers, Sprayers, and Applicators, Vegetation, Petroleum Pump System Operators, Refinery Operators, and Gaugers, Pile-Driver Operators, Pipelayers, Plant and System Operators, All Other, Plasterers and Stucco Masons, Plating and Coating Machine Setters, Operators, and Tenders, Metal and Plastic, Plumbers, Pipefitters, and Steamfitters, Pourers and Casters, Metal, Power Plant Operators, Pressers, Textile, Garment, and Related Materials, Print Binding and Finishing Workers, Printing Press Operators, Pump Operators, Except Wellhead Pumpers, Rail Car Repairers, Rail Yard Engineers, Dinkey Operators, and Hostlers, Rail-Track Laying and Maintenance Equipment Operators, Railroad Brake, Signal, and Switch Operators, Recreational Vehicle Service Technicians, Refractory Materials Repairers, Except Brickmasons, Refuse and Recyclable Material Collectors, Reinforcing Iron and Rebar Workers, Riggers, Rock Splitters, Quarry, Rolling Machine Setters, Operators, and Tenders, Metal and Plastic, Roof Bolters, Mining, Roofers, Rotary Drill Operators, Oil and Gas, Roustabouts, Oil and Gas, Sailors and Marine Oilers, Sawing Machine Setters, Operators, and Tenders, Wood, Security and Fire Alarm Systems Installers, Semiconductor Processors, Separating, Filtering, Clarifying, Precipitating, and Still Machine Setters, Operators, and Tenders, Septic Tank Servicers and Sewer Pipe Cleaners, Sewing Machine Operators, Sheet Metal Workers, Shoe Machine Operators and Tenders, Shoe and Leather Workers and Repairers, Signal and Track Switch Repairers, Slaughterers and Meat Packers, Stonemasons, Structural Iron and Steel Workers, Structural Metal Fabricators and Fitters, Subway and Streetcar Operators, Tailors, Dressmakers, and Custom Sewers, Tank Car, Truck, and Ship Loaders, Tapers, Taxi Drivers and Chauffeurs, Team Assemblers, Telecommunications Line Installers and Repairers, Terrazzo Workers and Finishers, Textile Bleaching and Dyeing Machine Operators and Tenders, Textile Cutting Machine Setters, Operators, and Tenders, Textile Knitting and Weaving Machine Setters, Operators, and Tenders, Textile Winding, Twisting, and Drawing Out Machine Setters, Operators, and Tenders, Tile and Marble Setters, Timing Device Assemblers and Adjusters, Tire Builders, Tire Repairers and Changers, Tool Grinders, Filers, and Sharpeners, Tool and Die Makers, Tree Trimmers and Pruners, Upholsterers, Watch Repairers, Welders, Cutters, Solderers, and Brazers, Welding, Soldering, and Brazing Machine Setters, Operators, and Tenders, Wellhead Pumpers, Woodworking Machine Setters, Operators, and Tenders, Except Sawing

Blue (0.94)	Actors, Amusement and Recreation Attendants, Athletes and Sports Competitors, Baggage Porters and Bellhops, Bailiffs, Barbers, Bartenders, Bill and Account Collectors, Billing and Posting Clerks, Bookkeeping, Accounting, and Auditing Clerks, Brokerage Clerks, Cashiers, Childcare Workers, Combined Food Preparation and Serving Workers, Including Fast Food, Cooks, Fast Food, Cooks, Institution and Cafeteria, Cooks, Short Order, Correspondence Clerks, Costume Attendants, Counter Attendants, Cafeteria, Food Concession, and Coffee Shop, Counter and Rental Clerks, Court Reporters, Court, Municipal, and License Clerks, Crossing Guards, Dancers, Data Entry Keyers, Demonstrators and Product Promoters, Dining Room and Cafeteria Attendants and Bartender Helpers, Door-to-Door Sales Workers, News and Street Vendors, and Related Workers, Driver/Sales Workers, Farm Labor Contractors, File Clerks, Fine Artists, Including Painters, Sculptors, and Illustrators, First-Line Supervisors of Food Preparation and Serving Workers, First-Line Supervisors of Housekeeping and Janitorial Workers, First-Line Supervisors of Retail Sales Workers, Fitness Trainers and Aerobics Instructors, Flight Attendants, Floral Designers, Food Preparation Workers, Food Servers, Nonrestaurant, Funeral Attendants, Gaming Cage Workers, Gaming Change Persons and Booth Cashiers, Gaming Dealers, Gaming and Sports Book Writers and Runners, Graders and Sorters, Agricultural Products, Hairdressers, Hairstylists, and Cosmetologists, Home Health Aides, Hosts and Hostesses, Restaurant, Lounge, and Coffee Shop, Hotel, Motel, and Resort Desk Clerks, Inspectors, Testers, Sorters, Samplers, and Weighers, Insurance Appraisers, Auto Damage, Insurance Claims and Policy Processing Clerks, Interviewers, Except Eligibility and Loan, Legal Secretaries, Library Assistants, Clerical, Library Technicians, Lifeguards, Ski Patrol, and Other Recreational Protective Service Workers, Locker Room, Coatroom, and Dressing Room Attendants, Maids and Housekeeping Cleaners, Mail Clerks and Mail Machine Operators, Except Postal Service, Makeup Artists, Theatrical and Performance, Manicurists and Pedicurists, Massage Therapists, Medical Records and Health Information Technicians, Medical Secretaries, Medical Transcriptionists, Merchandise Displayers and Window Trimmers, Models, Musicians and Singers, Nonfarm Animal Caretakers, Occupational Therapy Aides, Office Clerks, General, Order Clerks, Parking Enforcement Workers, Parts Salespersons, Payroll and Timekeeping Clerks, Personal Care Aides, Pharmacy Aides, Pharmacy Technicians, Photographic Process Workers and Processing Machine Operators, Physical Therapist Aides, Postal Service Clerks, Postal Service Mail Carriers, Postal Service Mail Sorters, Processors, and Processing Machine Operators, Prepress Technicians and Workers, Proofreaders and Copy Markers, Psychiatric Aides, Public Address System and Other Announcers, Radio Operators, Receptionists and Information Clerks, Reservation and Transportation Ticket Agents and Travel Clerks, Retail Salespersons, Secretaries and Administrative Assistants, Except Legal, Medical, and Executive, Security Guards, Self-Enrichment Education Teachers, Sewers, Hand, Shampooers, Shipping, Receiving, and Traffic Clerks, Skincare Specialists, Slot Supervisors, Stock Clerks and Order Fillers, Switchboard Operators, Including Answering Service, Teacher Assistants, Telemarketers, Telephone Operators, Tellers, Title Examiners, Abstractors, and Searchers, Tour Guides and Escorts, Umpires, Referees, and Other Sports Officials, Ushers, Lobby Attendants, and Ticket Takers, Waiters and Waitresses, Weighers, Measurers, Checkers, and Samplers, Recordkeeping, Word Processors and Typists

6.3.1 Alternative Job Groups using K-means

We demonstrate that our choice to focus on five groups of jobs according to skills produces results that are consistent for several alternative numbers of groups. Using K-means to identify between three and seven job groups continues demonstrate that computational/analytical and managerial skill are more indicative of super linear job growth, while physical skills are more indicative of linear or sub linear job growth. Likewise, our conclusions relating job scaling to expected job impact by comparing skills hold as well.

K-means clustering of similar jobs ($k = 3$)

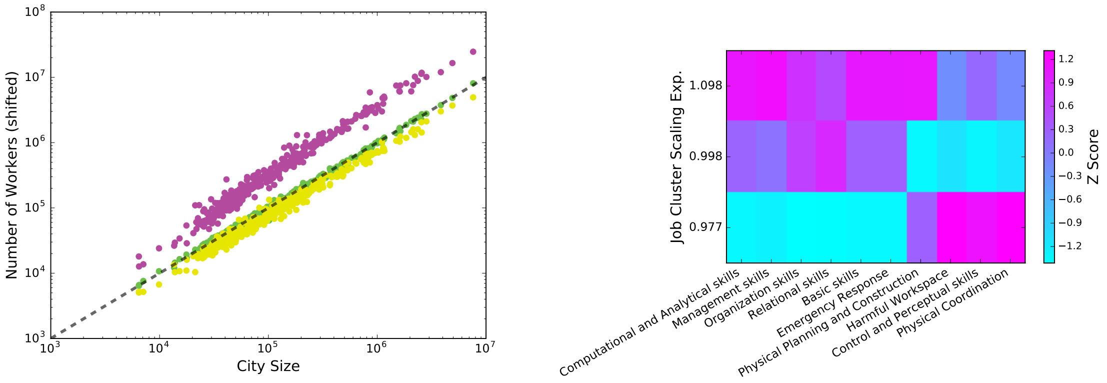

K-means clustering of similar jobs ($k = 4$)

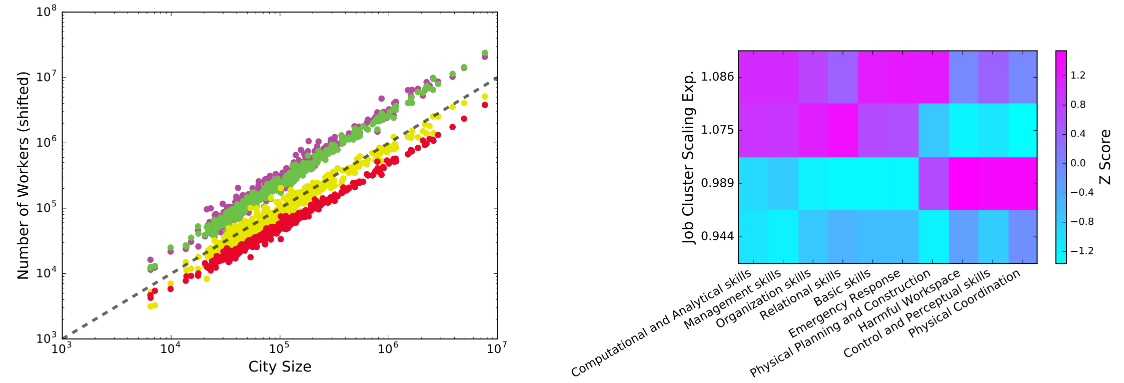

K-means clustering of similar jobs ($k = 5$)

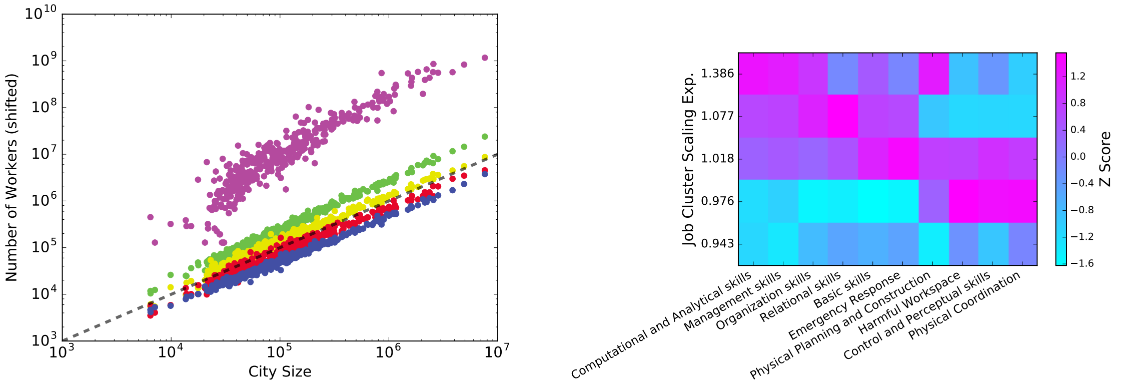

K-means clustering of similar jobs ($k = 6$)

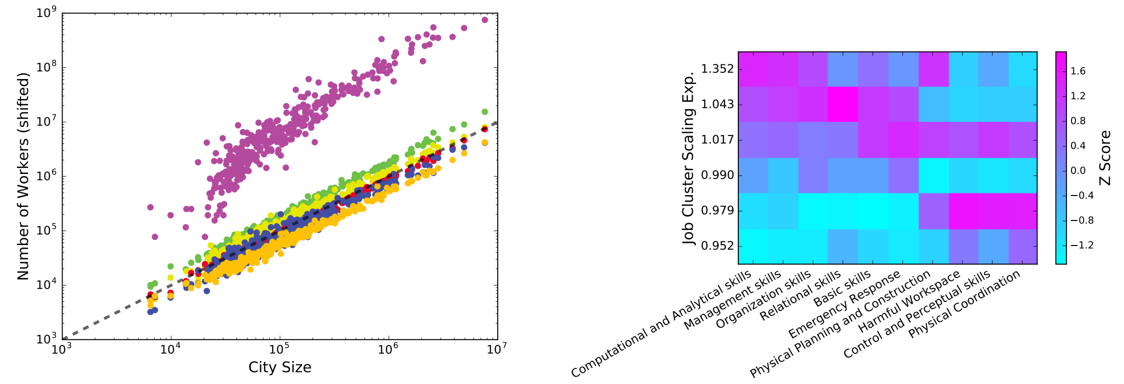

K-means clustering of similar jobs ($k = 7$)

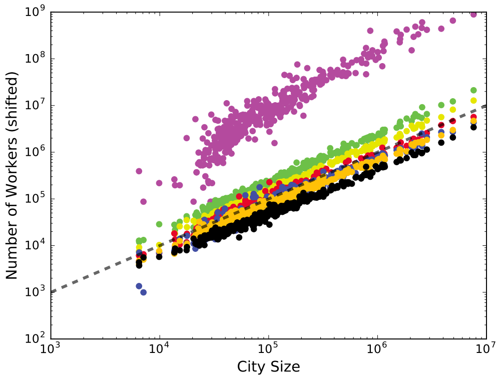

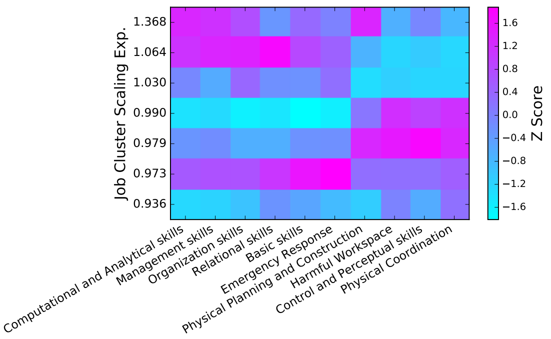

6.3.2 Stability Testing for Job Groups

We want to test the stability of the scaling results we observe when using five job clusters obtained from k-means clustering. In particular, how robust to sub-sampling is our observation that one job clusters scales faster than the rest? For a single trial, we sub-sample from the complete list of BLS occupations (percent indicated in plot titles) to obtain a matrix where each row represents a single occupation which was sub-sampled and each column represents the raw O*NET importance of a skill to each occupation. We apply k-means to this occupation-skill matrix (i.e. occupations are instances and skills are features) to obtain five occupation clusters (note: examination of prescribing between three and seven clusters is discussed in the SM). We then measure the scaling exponent (β) of each occupation cluster and rank the occupation clusters according to scaling exponent (rank indicated by color in plots). We perform 100 independent trials for each sub-sampling proportion in $\{10\%, 20\%, \ldots, 90\%, 100\%\}$ and plot the resulting scaling exponent distributions. In good agreement with our original findings, we find that indeed one occupation cluster (indicated in purple) tends to grow much faster than the other occupation clusters despite varying sub-sampling of occupations.

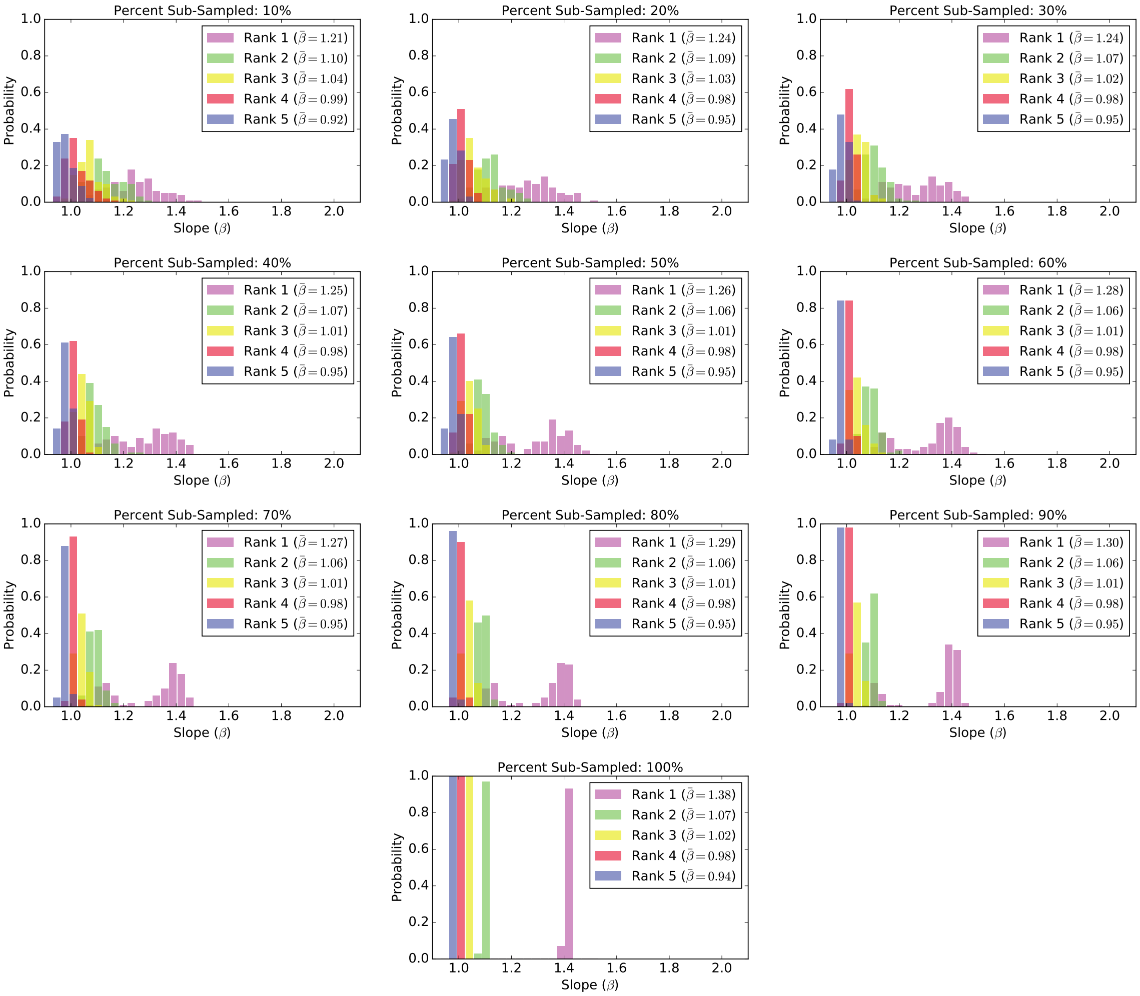

Figure 15: Boot-strapping at various rates of sub-sampling demonstrates the stability of our result that one job cluster scales at a greater rate than the rest when using five clusters obtained from k-means clustering.

6.3.3 Checking the Statistical Robustness of Job Group Scaling

Readers who are familiar with the urban scaling literature may be aware of an ongoing debate about the statistical significance of exponent measurements and identification of underlying statistical models to explain that growth. For example, what model should one assume to test if a trend is significantly superlinear? Rather than solving this ongoing and important problem, the goal of this study is only to understand the relationship between automation and urbanization. Our narrative requires only that highly specialized occupations (represented by purple dots in Figure 3A of the main text) exhibit superlinear growth and be notably different from the growth exhibited by other occupations.

Recent work by Leitao et al. [22] proposes several statistical models that may explain urban scaling trends, and they apply them to a variety of datasets to test the models' ability to explain urban scaling. Here, we employ these same models to test if our requirements on the scaling of highly specialized occupations are met according to the five job groups discussed in the main text (i.e. K-means clustering with $k = 5$). As an example, Figure 16 provides estimates of the scaling exponent along with standard errors for the scaling of each job group according to the unconstrained logarithm model. Table 9 details the complete analysis in line with the methods in [22]. For each model tested, we find that the purple job group, which represents highly specialized occupations, exhibits significantly superlinear scaling, and, furthermore, consistently exhibits faster growth rates than other job groups.

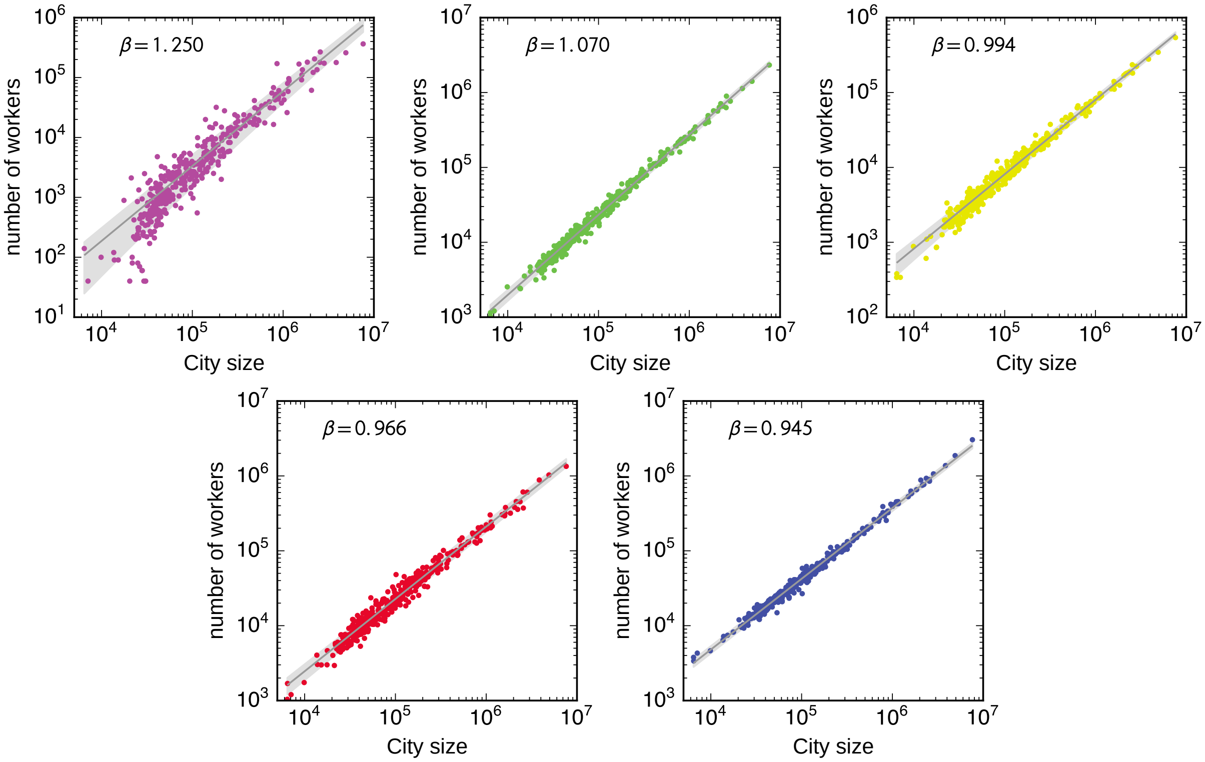

Figure 16: Following the work of Leitao et al., we provide slope estimates along with standard errors for the scaling of each occupation cluster according to unconstrained logarithm model.

	city model				
	lognormal		Gaussian		person model
data	$\delta = 2$	$\delta \in [1,3]$	$\delta = 1$	$\delta \in [1,2]$	
purple	1.380 (0.226)$\nearrow$	1.250 (0.079)$\nearrow$	1.289 (0.234)$\nearrow$	1.284 (0.065)$\nearrow$	1.130 (0.102)$\nearrow$
green	1.075 (0.097)$\nearrow$	1.070 (0.044)$\nearrow$	1.065 (0.013)$\nearrow$	1.071 (0.009)$\nearrow^*$	1.061 (0.012)$\nearrow$
yellow	1.021 (0.060)$\circ^*$	0.994 (0.040)$\rightarrow$	0.980 (0.018)$\searrow$	0.996 (0.014)$\rightarrow$	0.976 (0.017)$\searrow$
red	0.976 (0.038)$\circ^*$	0.966 (0.013)$\searrow^*$	0.963 (0.022)$\searrow$	0.966 (0.015)$\searrow$	0.965 (0.020)$\searrow$
blue	0.943 (0.009)$\searrow$	0.945 (0.010)$\searrow^*$	0.974 (0.019)$\searrow$	0.945 (0.009)$\searrow$	0.971 (0.018)$\searrow$

Table 9: An analysis of scaling exponents following the work of Leitao et al. The entries in the table represent the scaling exponent β. The value obtained through least-squares fitting in log-scale coincides with the value reported in the first column. The error bars were computed with bootstrap. The asterisk indicates that the model has a p-value higher than 0.05. If the difference ΔBIC between the BIC of each model with the same model with a fixed $\beta = 1$ is below 0, the model is linear ($\rightarrow$), between zero and six is inconclusive ($\circ$) and higher than six (strong evidence) is super-linear ($\nearrow$)/sublinear ($\searrow$).

6.4 Relating City Trends to O*NET Skill

Skill	Corr. to Job Impact	Corr to $H_{skill}(m)$	Corr. to Log_{10} City Size
Thinking Creatively	-0.71 ($< 10^{-56}$)	-0.24 ($< 10^{-4}$)	0.75 ($< 10^{-67}$)
Category Flexibility	-0.70 ($< 10^{-56}$)	-0.22 ($< 10^{-3}$)	0.63 ($< 10^{-41}$)
Inductive Reasoning	-0.70 ($< 10^{-56}$)	-0.27 ($< 10^{-5}$)	0.62 ($< 10^{-40}$)
Deductive Reasoning	-0.70 ($< 10^{-55}$)	-0.27 ($< 10^{-6}$)	0.57 ($< 10^{-32}$)
Active Learning	-0.70 ($< 10^{-55}$)	-0.32 ($< 10^{-9}$)	0.52 ($< 10^{-26}$)
Problem Sensitivity	-0.70 ($< 10^{-54}$)	-0.22 ($< 10^{-3}$)	0.63 ($< 10^{-41}$)
Originality	-0.70 ($< 10^{-54}$)	-0.32 ($< 10^{-9}$)	0.57 ($< 10^{-32}$)
Information Ordering	-0.70 ($< 10^{-54}$)	-0.20 ($< 10^{-3}$)	0.65 ($< 10^{-46}$)
Critical Thinking	-0.69 ($< 10^{-54}$)	-0.29 ($< 10^{-6}$)	0.61 ($< 10^{-38}$)
Complex Problem Solving	-0.69 ($< 10^{-53}$)	-0.18 ($< 10^{-2}$)	0.69 ($< 10^{-52}$)
Interpreting the Meaning of Information for Others	-0.69 ($< 10^{-53}$)	-0.27 ($< 10^{-6}$)	0.65 ($< 10^{-44}$)
Writing	-0.68 ($< 10^{-52}$)	-0.40 ($< 10^{-14}$)	0.39 ($< 10^{-13}$)
Written Comprehension	-0.68 ($< 10^{-51}$)	-0.38 ($< 10^{-12}$)	0.41 ($< 10^{-15}$)
Artistic	-0.68 ($< 10^{-51}$)	-0.32 ($< 10^{-9}$)	0.79 ($< 10^{-81}$)
Reading Comprehension	-0.68 ($< 10^{-50}$)	-0.37 ($< 10^{-11}$)	0.43 ($< 10^{-16}$)
Judgment and Decision Making	-0.68 ($< 10^{-50}$)	-0.26 ($< 10^{-5}$)	0.52 ($< 10^{-25}$)
Science	-0.68 ($< 10^{-50}$)	-0.16 ($< 10^{-1}$)	0.80 ($< 10^{-84}$)
Written Expression	-0.68 ($< 10^{-50}$)	-0.40 ($< 10^{-14}$)	0.36 ($< 10^{-11}$)
Computers and Electronics	-0.67 ($< 10^{-49}$)	-0.25 ($< 10^{-4}$)	0.57 ($< 10^{-33}$)
Fluency of Ideas	-0.67 ($< 10^{-49}$)	-0.34 ($< 10^{-9}$)	0.47 ($< 10^{-20}$)
Analyzing Data or Information	-0.67 ($< 10^{-48}$)	-0.21 ($< 10^{-3}$)	0.66 ($< 10^{-46}$)
Investigative	-0.66 ($< 10^{-47}$)	-0.12 ($< 10^{0}$)	0.82 ($< 10^{-92}$)
Making Decisions and Solving Problems	-0.66 ($< 10^{-47}$)	-0.19 ($< 10^{-2}$)	0.72 ($< 10^{-59}$)
Communications and Media	-0.66 ($< 10^{-46}$)	-0.47 ($< 10^{-20}$)	0.50 ($< 10^{-23}$)
History and Archeology	-0.66 ($< 10^{-46}$)	-0.34 ($< 10^{-10}$)	0.61 ($< 10^{-37}$)
Updating and Using Relevant Knowledge	-0.65 ($< 10^{-45}$)	-0.25 ($< 10^{-4}$)	0.66 ($< 10^{-46}$)
Processing Information	-0.64 ($< 10^{-43}$)	-0.23 ($< 10^{-4}$)	0.56 ($< 10^{-31}$)
Developing Objectives and Strategies	-0.63 ($< 10^{-41}$)	-0.20 ($< 10^{-3}$)	0.46 ($< 10^{-19}$)
Programming	-0.63 ($< 10^{-41}$)	-0.14 ($< 10^{-1}$)	0.63 ($< 10^{-41}$)
Getting Information	-0.63 ($< 10^{-40}$)	-0.32 ($< 10^{-8}$)	0.57 ($< 10^{-31}$)
Spend Time Sitting	-0.63 ($< 10^{-40}$)	-0.40 ($< 10^{-14}$)	0.51 ($< 10^{-24}$)
Operations Analysis	-0.62 ($< 10^{-40}$)	-0.14 ($< 10^{-1}$)	0.66 ($< 10^{-46}$)
Systems Analysis	-0.62 ($< 10^{-40}$)	-0.21 ($< 10^{-3}$)	0.37 ($< 10^{-12}$)
Systems Evaluation	-0.61 ($< 10^{-38}$)	-0.19 ($< 10^{-2}$)	0.35 ($< 10^{-10}$)
Flexibility of Closure	-0.61 ($< 10^{-38}$)	-0.07 ($< 10^{0}$)	0.83 ($< 10^{-97}$)
Fine Arts	-0.61 ($< 10^{-37}$)	-0.34 ($< 10^{-10}$)	0.79 ($< 10^{-81}$)
Near Vision	-0.61 ($< 10^{-37}$)	-0.16 ($< 10^{-1}$)	0.78 ($< 10^{-76}$)
Electronic Mail	-0.61 ($< 10^{-37}$)	-0.42 ($< 10^{-15}$)	0.25 ($< 10^{-5}$)
Documenting/Recording Information	-0.60 ($< 10^{-37}$)	-0.21 ($< 10^{-3}$)	0.47 ($< 10^{-20}$)
Identifying Objects, Actions, and Events	-0.60 ($< 10^{-37}$)	-0.24 ($< 10^{-4}$)	0.67 ($< 10^{-49}$)
Geography	-0.60 ($< 10^{-36}$)	-0.26 ($< 10^{-5}$)	0.61 ($< 10^{-37}$)
Technology Design	-0.60 ($< 10^{-36}$)	0.00 ($< 10^{0}$)	0.72 ($< 10^{-60}$)
Biology	-0.60 ($< 10^{-36}$)	-0.32 ($< 10^{-8}$)	0.63 ($< 10^{-41}$)
Freedom to Make Decisions	-0.60 ($< 10^{-36}$)	-0.21 ($< 10^{-3}$)	0.75 ($< 10^{-68}$)
Speed of Closure	-0.59 ($< 10^{-35}$)	-0.13 ($< 10^{0}$)	0.54 ($< 10^{-28}$)
Scheduling Work and Activities	-0.58 ($< 10^{-34}$)	-0.23 ($< 10^{-4}$)	0.31 ($< 10^{-8}$)

Selective Attention	-0.58 ($< 10^{-33}$)	0.03 ($< 10^{0}$)	0.84 ($< 10^{-101}$)
Education and Training	-0.58 ($< 10^{-33}$)	-0.08 ($< 10^{0}$)	0.34 ($< 10^{-10}$)
Estimating the Quantifiable Characteristics of Products, Events, or Information	-0.58 ($< 10^{-33}$)	-0.02 ($< 10^{0}$)	0.81 ($< 10^{-86}$)
Interacting With Computers	-0.57 ($< 10^{-32}$)	-0.30 ($< 10^{-7}$)	0.34 ($< 10^{-9}$)
Mathematical Reasoning	-0.56 ($< 10^{-31}$)	-0.19 ($< 10^{-2}$)	0.29 ($< 10^{-7}$)
Mathematics	-0.56 ($< 10^{-31}$)	-0.04 ($< 10^{0}$)	0.52 ($< 10^{-25}$)
Judging the Qualities of Things, Services, or People	-0.56 ($< 10^{-30}$)	-0.11 ($< 10^{0}$)	0.58 ($< 10^{-33}$)
English Language	-0.56 ($< 10^{-30}$)	-0.43 ($< 10^{-16}$)	0.17 ($< 10^{-2}$)
Provide Consultation and Advice to Others	-0.55 ($< 10^{-29}$)	-0.21 ($< 10^{-3}$)	0.27 ($< 10^{-6}$)
Monitoring	-0.55 ($< 10^{-29}$)	-0.07 ($< 10^{0}$)	0.26 ($< 10^{-5}$)
Physics	-0.53 ($< 10^{-27}$)	0.11 ($< 10^{0}$)	0.83 ($< 10^{-94}$)
Design	-0.53 ($< 10^{-26}$)	0.09 ($< 10^{0}$)	0.83 ($< 10^{-96}$)
Structured versus Unstructured Work	-0.53 ($< 10^{-26}$)	-0.34 ($< 10^{-9}$)	0.33 ($< 10^{-9}$)
Engineering and Technology	-0.52 ($< 10^{-26}$)	0.13 ($< 10^{0}$)	0.82 ($< 10^{-90}$)
Visualization	-0.52 ($< 10^{-25}$)	0.06 ($< 10^{0}$)	0.87 ($< 10^{-114}$)
Oral Comprehension	-0.52 ($< 10^{-25}$)	-0.48 ($< 10^{-21}$)	0.07 ($< 10^{0}$)
Memorization	-0.51 ($< 10^{-24}$)	-0.27 ($< 10^{-6}$)	0.08 ($< 10^{0}$)
Duration of Typical Work Week	-0.50 ($< 10^{-23}$)	0.17 ($< 10^{-2}$)	0.67 ($< 10^{-49}$)
Level of Competition	-0.50 ($< 10^{-23}$)	-0.16 ($< 10^{-1}$)	0.79 ($< 10^{-80}$)
Oral Expression	-0.49 ($< 10^{-22}$)	-0.48 ($< 10^{-21}$)	0.03 ($< 10^{0}$)
Organizing, Planning, and Prioritizing Work	-0.49 ($< 10^{-22}$)	-0.33 ($< 10^{-9}$)	0.09 ($< 10^{0}$)
Number Facility	-0.48 ($< 10^{-21}$)	-0.17 ($< 10^{-2}$)	0.21 ($< 10^{-3}$)
Active Listening	-0.48 ($< 10^{-21}$)	-0.49 ($< 10^{-22}$)	0.04 ($< 10^{0}$)
Instructing	-0.48 ($< 10^{-21}$)	-0.22 ($< 10^{-3}$)	-0.03 ($< 10^{0}$)
Telecommunications	-0.47 ($< 10^{-20}$)	-0.22 ($< 10^{-3}$)	0.42 ($< 10^{-15}$)
Indoors, Environmentally Controlled	-0.47 ($< 10^{-20}$)	-0.54 ($< 10^{-28}$)	0.21 ($< 10^{-3}$)
Learning Strategies	-0.47 ($< 10^{-20}$)	-0.26 ($< 10^{-5}$)	-0.06 ($< 10^{0}$)
Chemistry	-0.47 ($< 10^{-20}$)	0.13 ($< 10^{-1}$)	0.78 ($< 10^{-75}$)
Far Vision	-0.46 ($< 10^{-19}$)	0.08 ($< 10^{0}$)	0.71 ($< 10^{-57}$)
Monitor Processes, Materials, or Surroundings	-0.46 ($< 10^{-19}$)	0.14 ($< 10^{-1}$)	0.65 ($< 10^{-45}$)
Drafting, Laying Out, and Specifying Technical Devices, Parts, and Equipment	-0.45 ($< 10^{-18}$)	0.18 ($< 10^{-2}$)	0.80 ($< 10^{-84}$)
Perceptual Speed	-0.45 ($< 10^{-18}$)	0.16 ($< 10^{-1}$)	0.77 ($< 10^{-74}$)
Evaluating Information to Determine Compliance with Standards	-0.44 ($< 10^{-17}$)	-0.00 ($< 10^{0}$)	0.30 ($< 10^{-7}$)
Public Speaking	-0.44 ($< 10^{-17}$)	-0.31 ($< 10^{-8}$)	-0.03 ($< 10^{0}$)
Speaking	-0.43 ($< 10^{-16}$)	-0.47 ($< 10^{-21}$)	-0.07 ($< 10^{0}$)
Importance of Being Exact or Accurate	-0.42 ($< 10^{-15}$)	-0.07 ($< 10^{0}$)	0.80 ($< 10^{-84}$)
Visual Color Discrimination	-0.41 ($< 10^{-14}$)	0.09 ($< 10^{0}$)	0.84 ($< 10^{-101}$)
Communicating with Supervisors, Peers, or Subordinates	-0.40 ($< 10^{-14}$)	-0.16 ($< 10^{-1}$)	-0.01 ($< 10^{0}$)
Face-to-Face Discussions	-0.38 ($< 10^{-12}$)	-0.11 ($< 10^{0}$)	0.34 ($< 10^{-9}$)
Letters and Memos	-0.38 ($< 10^{-12}$)	-0.37 ($< 10^{-12}$)	-0.12 ($< 10^{0}$)
Impact of Decisions on Co-workers or Company Results	-0.37 ($< 10^{-12}$)	-0.14 ($< 10^{-1}$)	0.48 ($< 10^{-21}$)
Time Management	-0.37 ($< 10^{-11}$)	-0.27 ($< 10^{-5}$)	-0.18 ($< 10^{-2}$)
Training and Teaching Others	-0.36 ($< 10^{-11}$)	-0.04 ($< 10^{0}$)	0.02 ($< 10^{0}$)
Quality Control Analysis	-0.36 ($< 10^{-11}$)	0.25 ($< 10^{-5}$)	0.80 ($< 10^{-84}$)

Consequence of Error	-0.35 ($< 10^{-10}$)	0.16 ($< 10^{-1}$)	0.75 ($< 10^{-69}$)
Communicating with Persons Outside Organization	-0.35 ($< 10^{-10}$)	-0.50 ($< 10^{-23}$)	-0.03 ($< 10^{0}$)
Repairing and Maintaining Electronic Equipment	-0.35 ($< 10^{-10}$)	0.19 ($< 10^{-2}$)	0.81 ($< 10^{-88}$)
Speech Clarity	-0.35 ($< 10^{-10}$)	-0.47 ($< 10^{-20}$)	-0.18 ($< 10^{-2}$)
Philosophy and Theology	-0.34 ($< 10^{-9}$)	-0.35 ($< 10^{-10}$)	-0.06 ($< 10^{0}$)
Monitoring and Controlling Resources	-0.33 ($< 10^{-9}$)	-0.28 ($< 10^{-6}$)	0.01 ($< 10^{0}$)
Management of Personnel Resources	-0.33 ($< 10^{-9}$)	-0.18 ($< 10^{-2}$)	-0.19 ($< 10^{-2}$)
Sociology and Anthropology	-0.33 ($< 10^{-9}$)	-0.34 ($< 10^{-10}$)	-0.13 ($< 10^{0}$)
Equipment Selection	-0.28 ($< 10^{-6}$)	0.30 ($< 10^{-7}$)	0.79 ($< 10^{-80}$)
Law and Government	-0.27 ($< 10^{-6}$)	-0.27 ($< 10^{-5}$)	-0.08 ($< 10^{0}$)
Exposed to Radiation	-0.27 ($< 10^{-6}$)	-0.09 ($< 10^{0}$)	0.41 ($< 10^{-14}$)
Troubleshooting	-0.27 ($< 10^{-6}$)	0.32 ($< 10^{-8}$)	0.76 ($< 10^{-72}$)
Time Pressure	-0.27 ($< 10^{-6}$)	0.07 ($< 10^{0}$)	0.60 ($< 10^{-36}$)
Work Schedules	-0.26 ($< 10^{-5}$)	-0.01 ($< 10^{0}$)	0.71 ($< 10^{-57}$)
Operation Monitoring	-0.25 ($< 10^{-5}$)	0.33 ($< 10^{-9}$)	0.75 ($< 10^{-67}$)
Mechanical	-0.25 ($< 10^{-5}$)	0.38 ($< 10^{-12}$)	0.73 ($< 10^{-62}$)
Telephone	-0.23 ($< 10^{-4}$)	-0.44 ($< 10^{-17}$)	-0.17 ($< 10^{-2}$)
Persuasion	-0.23 ($< 10^{-4}$)	-0.32 ($< 10^{-8}$)	-0.27 ($< 10^{-6}$)
Management of Material Resources	-0.22 ($< 10^{-3}$)	-0.07 ($< 10^{0}$)	-0.24 ($< 10^{-4}$)
Production and Processing	-0.20 ($< 10^{-3}$)	0.35 ($< 10^{-10}$)	0.60 ($< 10^{-36}$)
Building and Construction	-0.20 ($< 10^{-3}$)	0.32 ($< 10^{-8}$)	0.51 ($< 10^{-24}$)
Wear Specialized Protective or Safety Equipment such as Breathing Apparatus, Safety Harness, Full Protection Suits, or Radiation Protection	-0.20 ($< 10^{-3}$)	0.32 ($< 10^{-8}$)	0.66 ($< 10^{-47}$)
Management of Financial Resources	-0.19 ($< 10^{-2}$)	-0.18 ($< 10^{-2}$)	-0.26 ($< 10^{-5}$)
Depth Perception	-0.18 ($< 10^{-2}$)	0.34 ($< 10^{-10}$)	0.73 ($< 10^{-61}$)
Establishing and Maintaining Interpersonal Relationships	-0.18 ($< 10^{-2}$)	-0.41 ($< 10^{-15}$)	-0.35 ($< 10^{-10}$)
Exposed to Hazardous Conditions	-0.17 ($< 10^{-2}$)	0.37 ($< 10^{-12}$)	0.69 ($< 10^{-53}$)
Hearing Sensitivity	-0.17 ($< 10^{-2}$)	0.40 ($< 10^{-14}$)	0.60 ($< 10^{-37}$)
Coaching and Developing Others	-0.16 ($< 10^{-1}$)	-0.13 ($< 10^{-1}$)	-0.39 ($< 10^{-13}$)
Finger Dexterity	-0.16 ($< 10^{-1}$)	0.24 ($< 10^{-4}$)	0.71 ($< 10^{-57}$)
Installation	-0.16 ($< 10^{-1}$)	0.37 ($< 10^{-11}$)	0.58 ($< 10^{-34}$)
Developing and Building Teams	-0.15 ($< 10^{-1}$)	-0.11 ($< 10^{0}$)	-0.39 ($< 10^{-13}$)
Guiding, Directing, and Motivating Subordinates	-0.15 ($< 10^{-1}$)	-0.08 ($< 10^{0}$)	-0.30 ($< 10^{-7}$)
Equipment Maintenance	-0.15 ($< 10^{-1}$)	0.38 ($< 10^{-12}$)	0.69 ($< 10^{-54}$)
Repairing	-0.14 ($< 10^{-1}$)	0.40 ($< 10^{-14}$)	0.68 ($< 10^{-51}$)
Medicine and Dentistry	-0.13 ($< 10^{-1}$)	-0.27 ($< 10^{-6}$)	-0.11 ($< 10^{0}$)
Third Interest High-Point	-0.13 ($< 10^{0}$)	-0.07 ($< 10^{0}$)	-0.18 ($< 10^{-2}$)
Negotiation	-0.12 ($< 10^{0}$)	-0.27 ($< 10^{-6}$)	-0.42 ($< 10^{-15}$)
Inspecting Equipment, Structures, or Material	-0.12 ($< 10^{0}$)	0.39 ($< 10^{-13}$)	0.64 ($< 10^{-43}$)
Operation and Control	-0.12 ($< 10^{0}$)	0.36 ($< 10^{-11}$)	0.69 ($< 10^{-52}$)
Realistic	-0.11 ($< 10^{0}$)	0.31 ($< 10^{-8}$)	0.70 ($< 10^{-55}$)
Coordination	-0.11 ($< 10^{0}$)	-0.20 ($< 10^{-3}$)	-0.50 ($< 10^{-23}$)
Controlling Machines and Processes	-0.10 ($< 10^{0}$)	0.38 ($< 10^{-12}$)	0.67 ($< 10^{-48}$)
Coordinating the Work and Activities of Others	-0.10 ($< 10^{0}$)	-0.10 ($< 10^{0}$)	-0.45 ($< 10^{-18}$)

Social Perceptiveness	-0.10 ($< 10^{0}$)	-0.30 ($< 10^{-7}$)	-0.46 ($< 10^{-19}$)
Auditory Attention	-0.09 ($< 10^{0}$)	0.47 ($< 10^{-20}$)	0.58 ($< 10^{-33}$)
Speech Recognition	-0.08 ($< 10^{0}$)	-0.38 ($< 10^{-12}$)	-0.50 ($< 10^{-23}$)
Repairing and Maintaining Mechanical Equipment	-0.08 ($< 10^{0}$)	0.43 ($< 10^{-17}$)	0.63 ($< 10^{-41}$)
Foreign Language	-0.07 ($< 10^{0}$)	-0.21 ($< 10^{-3}$)	-0.33 ($< 10^{-9}$)
Psychology	-0.07 ($< 10^{0}$)	-0.27 ($< 10^{-5}$)	-0.42 ($< 10^{-16}$)
Degree of Automation	-0.07 ($< 10^{0}$)	0.18 ($< 10^{-2}$)	0.36 ($< 10^{-11}$)
Administration and Management	-0.06 ($< 10^{0}$)	-0.11 ($< 10^{0}$)	-0.46 ($< 10^{-19}$)
Staffing Organizational Units	-0.06 ($< 10^{0}$)	-0.20 ($< 10^{-3}$)	-0.44 ($< 10^{-18}$)
Spend Time Using Your Hands to Handle, Control, or Feel Objects, Tools, or Controls	-0.06 ($< 10^{0}$)	0.22 ($< 10^{-3}$)	0.65 ($< 10^{-45}$)
Sales and Marketing	-0.03 ($< 10^{0}$)	-0.31 ($< 10^{-8}$)	-0.18 ($< 10^{-2}$)
Control Precision	-0.03 ($< 10^{0}$)	0.34 ($< 10^{-9}$)	0.64 ($< 10^{-43}$)
Performing Administrative Activities	-0.03 ($< 10^{0}$)	-0.29 ($< 10^{-7}$)	-0.51 ($< 10^{-25}$)
Exposed to Hazardous Equipment	-0.03 ($< 10^{0}$)	0.49 ($< 10^{-22}$)	0.56 ($< 10^{-30}$)
Exposed to High Places	-0.03 ($< 10^{0}$)	0.43 ($< 10^{-16}$)	0.47 ($< 10^{-20}$)
Rate Control	-0.02 ($< 10^{0}$)	0.42 ($< 10^{-15}$)	0.61 ($< 10^{-37}$)
Indoors, Not Environmentally Controlled	-0.01 ($< 10^{0}$)	0.52 ($< 10^{-26}$)	0.48 ($< 10^{-21}$)
Public Safety and Security	-0.00 ($< 10^{0}$)	0.21 ($< 10^{-3}$)	-0.03 ($< 10^{0}$)
Reaction Time	-0.00 ($< 10^{0}$)	0.44 ($< 10^{-17}$)	0.57 ($< 10^{-32}$)
Selling or Influencing Others	0.00 ($< 10^{0}$)	-0.30 ($< 10^{-7}$)	-0.34 ($< 10^{-9}$)
Pace Determined by Speed of Equipment	0.01 ($< 10^{0}$)	0.40 ($< 10^{-14}$)	0.53 ($< 10^{-27}$)
Clerical	0.02 ($< 10^{0}$)	-0.24 ($< 10^{-4}$)	-0.56 ($< 10^{-31}$)
Required Level of Education	0.02 ($< 10^{0}$)	-0.04 ($< 10^{0}$)	0.02 ($< 10^{0}$)
Glare Sensitivity	0.03 ($< 10^{0}$)	0.43 ($< 10^{-16}$)	0.50 ($< 10^{-23}$)
In an Enclosed Vehicle or Equipment	0.04 ($< 10^{0}$)	0.07 ($< 10^{0}$)	-0.00 ($< 10^{0}$)
Extremely Bright or Inadequate Lighting	0.04 ($< 10^{0}$)	0.45 ($< 10^{-18}$)	0.46 ($< 10^{-19}$)
Therapy and Counseling	0.04 ($< 10^{0}$)	-0.13 ($< 10^{0}$)	-0.49 ($< 10^{-22}$)
Frequency of Decision Making	0.04 ($< 10^{0}$)	-0.09 ($< 10^{0}$)	-0.06 ($< 10^{0}$)
Outdoors, Under Cover	0.05 ($< 10^{0}$)	0.25 ($< 10^{-5}$)	0.12 ($< 10^{0}$)
Personnel and Human Resources	0.06 ($< 10^{0}$)	-0.10 ($< 10^{0}$)	-0.59 ($< 10^{-35}$)
Transportation	0.06 ($< 10^{0}$)	0.29 ($< 10^{-6}$)	0.11 ($< 10^{0}$)
Wear Common Protective or Safety Equipment such as Safety Shoes, Glasses, Gloves, Hearing Protection, Hard Hats, or Life Jackets	0.06 ($< 10^{0}$)	0.52 ($< 10^{-25}$)	0.45 ($< 10^{-18}$)
On-the-Job Training	0.06 ($< 10^{0}$)	-0.02 ($< 10^{0}$)	-0.10 ($< 10^{0}$)
Responsibility for Outcomes and Results	0.07 ($< 10^{0}$)	0.27 ($< 10^{-5}$)	-0.17 ($< 10^{-2}$)
Work With Work Group or Team	0.07 ($< 10^{0}$)	-0.11 ($< 10^{0}$)	-0.56 ($< 10^{-31}$)
Arm-Hand Steadiness	0.07 ($< 10^{0}$)	0.30 ($< 10^{-7}$)	0.51 ($< 10^{-24}$)
Coordinate or Lead Others	0.08 ($< 10^{0}$)	-0.04 ($< 10^{0}$)	-0.63 ($< 10^{-41}$)
Exposed to Contaminants	0.09 ($< 10^{0}$)	0.50 ($< 10^{-23}$)	0.44 ($< 10^{-17}$)
Related Work Experience	0.09 ($< 10^{0}$)	0.16 ($< 10^{-1}$)	-0.34 ($< 10^{-9}$)
Spatial Orientation	0.10 ($< 10^{0}$)	0.42 ($< 10^{-16}$)	0.34 ($< 10^{-9}$)
Sounds, Noise Levels Are Distracting or Uncomfortable	0.10 ($< 10^{0}$)	0.56 ($< 10^{-30}$)	0.34 ($< 10^{-9}$)
Wrist-Finger Speed	0.11 ($< 10^{0}$)	0.37 ($< 10^{-12}$)	0.44 ($< 10^{-17}$)
Response Orientation	0.11 ($< 10^{0}$)	0.45 ($< 10^{-18}$)	0.40 ($< 10^{-14}$)
Night Vision	0.12 ($< 10^{0}$)	0.42 ($< 10^{-15}$)	0.33 ($< 10^{-9}$)

Cramped Work Space, Awkward Positions	0.12 ($< 10^{0}$)	0.46 ($< 10^{-19}$)	0.37 ($< 10^{-12}$)
Spend Time Climbing Ladders, Scaffolds, or Poles	0.12 ($< 10^{0}$)	0.47 ($< 10^{-20}$)	0.26 ($< 10^{-5}$)
On-Site or In-Plant Training	0.12 ($< 10^{0}$)	-0.05 ($< 10^{0}$)	-0.25 ($< 10^{-5}$)
Operating Vehicles, Mechanized Devices, or Equipment	0.13 ($< 10^{0}$)	0.49 ($< 10^{-22}$)	0.33 ($< 10^{-9}$)
Economics and Accounting	0.13 ($< 10^{-1}$)	-0.21 ($< 10^{-3}$)	-0.57 ($< 10^{-31}$)
Sound Localization	0.14 ($< 10^{-1}$)	0.49 ($< 10^{-23}$)	0.31 ($< 10^{-7}$)
Manual Dexterity	0.16 ($< 10^{-1}$)	0.33 ($< 10^{-9}$)	0.44 ($< 10^{-17}$)
Peripheral Vision	0.17 ($< 10^{-1}$)	0.46 ($< 10^{-19}$)	0.26 ($< 10^{-5}$)
Importance of Repeating Same Tasks	0.19 ($< 10^{-2}$)	0.04 ($< 10^{0}$)	-0.08 ($< 10^{0}$)
Outdoors, Exposed to Weather	0.20 ($< 10^{-3}$)	0.27 ($< 10^{-5}$)	-0.06 ($< 10^{0}$)
Service Orientation	0.21 ($< 10^{-3}$)	-0.28 ($< 10^{-6}$)	-0.75 ($< 10^{-68}$)
Resolving Conflicts and Negotiating with Others	0.23 ($< 10^{-4}$)	-0.14 ($< 10^{-1}$)	-0.75 ($< 10^{-68}$)
Social	0.24 ($< 10^{-4}$)	-0.21 ($< 10^{-3}$)	-0.73 ($< 10^{-61}$)
Spend Time Making Repetitive Motions	0.25 ($< 10^{-5}$)	0.17 ($< 10^{-2}$)	0.18 ($< 10^{-2}$)
Multilimb Coordination	0.26 ($< 10^{-5}$)	0.42 ($< 10^{-15}$)	0.28 ($< 10^{-6}$)
Deal With External Customers	0.26 ($< 10^{-5}$)	-0.30 ($< 10^{-7}$)	-0.66 ($< 10^{-47}$)
Time Sharing	0.27 ($< 10^{-5}$)	0.00 ($< 10^{0}$)	-0.68 ($< 10^{-50}$)
In an Open Vehicle or Equipment	0.27 ($< 10^{-6}$)	0.56 ($< 10^{-30}$)	0.09 ($< 10^{0}$)
Exposed to Whole Body Vibration	0.28 ($< 10^{-6}$)	0.46 ($< 10^{-19}$)	-0.06 ($< 10^{0}$)
Customer and Personal Service	0.28 ($< 10^{-6}$)	-0.22 ($< 10^{-3}$)	-0.74 ($< 10^{-64}$)
Performing for or Working Directly with the Public	0.29 ($< 10^{-7}$)	-0.32 ($< 10^{-8}$)	-0.62 ($< 10^{-40}$)
Exposed to Disease or Infections	0.32 ($< 10^{-9}$)	-0.10 ($< 10^{0}$)	-0.69 ($< 10^{-53}$)
Contact With Others	0.33 ($< 10^{-9}$)	-0.12 ($< 10^{0}$)	-0.73 ($< 10^{-62}$)
Exposed to Minor Burns, Cuts, Bites, or Stings	0.38 ($< 10^{-12}$)	0.56 ($< 10^{-30}$)	0.03 ($< 10^{0}$)
Handling and Moving Objects	0.38 ($< 10^{-12}$)	0.43 ($< 10^{-16}$)	0.10 ($< 10^{0}$)
Assisting and Caring for Others	0.38 ($< 10^{-12}$)	-0.05 ($< 10^{0}$)	-0.78 ($< 10^{-77}$)
Gross Body Equilibrium	0.38 ($< 10^{-13}$)	0.49 ($< 10^{-22}$)	-0.02 ($< 10^{0}$)
Frequency of Conflict Situations	0.38 ($< 10^{-13}$)	0.01 ($< 10^{0}$)	-0.84 ($< 10^{-98}$)
Deal With Physically Aggressive People	0.39 ($< 10^{-13}$)	0.04 ($< 10^{0}$)	-0.73 ($< 10^{-61}$)
Explosive Strength	0.39 ($< 10^{-13}$)	0.20 ($< 10^{-3}$)	-0.44 ($< 10^{-17}$)
Very Hot or Cold Temperatures	0.40 ($< 10^{-13}$)	0.54 ($< 10^{-27}$)	-0.05 ($< 10^{0}$)
Enterprising	0.40 ($< 10^{-14}$)	-0.13 ($< 10^{0}$)	-0.86 ($< 10^{-109}$)
First Interest High-Point	0.40 ($< 10^{-14}$)	-0.13 ($< 10^{-1}$)	-0.89 ($< 10^{-127}$)
Performing General Physical Activities	0.41 ($< 10^{-15}$)	0.48 ($< 10^{-21}$)	0.01 ($< 10^{0}$)
Spend Time Keeping or Regaining Balance	0.45 ($< 10^{-18}$)	0.47 ($< 10^{-20}$)	-0.11 ($< 10^{0}$)
Static Strength	0.46 ($< 10^{-19}$)	0.43 ($< 10^{-16}$)	-0.07 ($< 10^{0}$)
Physical Proximity	0.47 ($< 10^{-20}$)	-0.03 ($< 10^{0}$)	-0.61 ($< 10^{-38}$)
Food Production	0.47 ($< 10^{-20}$)	0.04 ($< 10^{0}$)	-0.73 ($< 10^{-63}$)
Dynamic Strength	0.48 ($< 10^{-21}$)	0.45 ($< 10^{-18}$)	-0.10 ($< 10^{0}$)
Responsible for Others' Health and Safety	0.48 ($< 10^{-22}$)	0.50 ($< 10^{-23}$)	-0.49 ($< 10^{-22}$)
Trunk Strength	0.53 ($< 10^{-26}$)	0.41 ($< 10^{-14}$)	-0.21 ($< 10^{-3}$)
Deal With Unpleasant or Angry People	0.53 ($< 10^{-27}$)	0.03 ($< 10^{0}$)	-0.83 ($< 10^{-98}$)
Spend Time Kneeling, Crouching, Stooping, or Crawling	0.53 ($< 10^{-27}$)	0.44 ($< 10^{-17}$)	-0.36 ($< 10^{-11}$)
Dynamic Flexibility	0.53 ($< 10^{-27}$)	0.25 ($< 10^{-4}$)	-0.53 ($< 10^{-26}$)
Conventional	0.54 ($< 10^{-28}$)	0.07 ($< 10^{0}$)	-0.88 ($< 10^{-120}$)

Extent Flexibility	0.55 ($< 10^{-29}$)	0.45 ($< 10^{-19}$)	-0.21 ($< 10^{-3}$)
Spend Time Bending or Twisting the Body	0.57 ($< 10^{-31}$)	0.47 ($< 10^{-20}$)	-0.26 ($< 10^{-5}$)
Spend Time Standing	0.57 ($< 10^{-32}$)	0.37 ($< 10^{-11}$)	-0.42 ($< 10^{-15}$)
Gross Body Coordination	0.58 ($< 10^{-33}$)	0.41 ($< 10^{-15}$)	-0.36 ($< 10^{-11}$)
Speed of Limb Movement	0.60 ($< 10^{-37}$)	0.46 ($< 10^{-20}$)	-0.37 ($< 10^{-12}$)
Stamina	0.60 ($< 10^{-37}$)	0.38 ($< 10^{-13}$)	-0.44 ($< 10^{-17}$)
Second Interest High-Point	0.65 ($< 10^{-44}$)	0.20 ($< 10^{-3}$)	-0.71 ($< 10^{-58}$)
Spend Time Walking and Running	0.67 ($< 10^{-49}$)	0.33 ($< 10^{-9}$)	-0.66 ($< 10^{-46}$)

6.5 Skill Types

We provide example O*NET skills from each of ten skill types. These groups of skills are obtained from the co-occurrence of skills across jobs. The left column provides a subjective labelling for each skill type based on the skills comprising that cluster of skills.

Skill Types	O*NET Skills
Computational and Analytical Skills	Active Learning, Analyzing Data or Information, Communications and Media, Complex Problem Solving, Computers and Electronics, Developing Objectives and Strategies, Documenting/Recording Information, Fluency of Ideas, Instructing, Interacting With Computers, Interpreting the Meaning of Information for Others, Judgement and Decision Making, Learning Strategies, Making Decisions and Solving Problems, Mathematical Reasoning, Memorization, Number Facility, Originality, Processing Information, Provide Consultation and Advice to Others, Systems Analysis, Systems Evaluation, Updating and Using Relevant Knowledge
Physical Planning and Construction	Building and Construction, Chemistry, Design, Drafting, Engineering and Technology, Estimating the Quantifiable Characteristics of Products, Events, or Information, Explosive Strength, Far Vision, Installation, Perceptual Speed, Physics, Production and Processing, Public Safety and Security,Transportation, Visualization
Harmful Workspace Management Skills	Cramped Work Space, Awkward Positions, Dynamic Flexibility, Exposed to Contaminants, Exposed to Hazardous Conditions, Exposed to High Places, Exposed to Minor Burns, Cuts, Bites, or Stings, Exposed to Whole Body Vibration, Extremely Bright or Inadequate Lighting, Finger Dexterity, Outdoors, Exposed to Weather, Pace Determined by Speed of Equipment, Responsible for Others' Health and Safety, Sounds, Noise Levels Are Distracting or Uncomfortable, Spend Time Bending or Twisting the Body, Spend Time Climbing Ladders, Scaffolds, or Poles, Spend Time Keeping or Regaining Balance, Spend Time Kneeling, Crouching, Stooping, or Crawling, Spend Time Making Repetitive Motions, Spend Time Standing, Spend Time Using Your Hands to Handle, Control, or Feel Objects, Tools, or Controls, Spend Time Walking and Running, Very Hot or Cold Temperatures, Wear Specialized Protective or Safety Equipment such as Breathing Apparatus, Safety Harness, Full Protection Suits, or Radiation Protection
Relational/Social Skills	Customer and Personal Service, Education and Training, Fine Arts, Foreign Language, History and Archeology, Identifying Objects, Actions, and Events, Law and Government, Performing for or Working Directly with the Public, Philosophy and Theology, Psychology, Resolving Conflicts and Negotiating with Others , Sales and Marketing, Selling or Influencing Others, Sociology and Anthropology, Therapy and Counseling, Training and Teaching Others

Control and Perceptual Skills	Auditory Attention, Depth Perception, Equipment Maintenance, Equipment Selection, Glare Sensitivity, Hearing Sensitivity, Inspecting Equipment, Structures, or Material, Mechanical, Night Vision, Operating Vehicles, Mechanized Devices, or Equipment, Operation Monitoring, Operation and Control, Peripheral Vision, Quality Control Analysis, Rate Control, Repairing, Repairing and Maintaining Electronic Equipment, Repairing and Maintaining Mechanical Equipment, Response Orientation, Sound Localization, Spatial Orientation, Troubleshooting, Visual Color Discrimination
Emergency Response	Consequence of Error, Contact With Others ,Coordinate or Lead Others, Deal With External Customers, Deal With Physically Aggressive People, Deal With Unpleasant or Angry People, Degree of Automation, Exposed to Disease or Infections, Exposed to Radiation, Frequency of Conflict Situations, Importance of Being Exact or Accurate, Importance of Repeating Same Tasks, Indoors, Environmentally Controlled, Near Vision, Responsibility for Outcomes and Results, Selective Attention
Basic skills	Artistic, Assisting and Caring for Others, Biology, Conventional, Duration of Typical, Work Week, Electronic Mail, Enterprising, Face-to-Face Discussions, First Interest High-Point, Flexibility of Closure, Food Production, Freedom to Make Decisions, Frequency of Decision Making, Impact of Decisions on Coworkers or Company Results, In an Enclosed Vehicle or Equipment, In an Open Vehicle or Equipment, Indoors, Not Environmentally Controlled, Investigative, Letters and Memos, Level of Competition, Medicine and Dentistry, Monitor Processes, Materials, or Surroundings, On-Site or In-Plant Training, On-the-Job Training, Outdoors, Under Cover, Physical Proximity, Public Speaking, Realistic, Related Work Experience, Required Level of Education, Second Interest High-Point, Social, Structured versus Unstructured Work, Technology Design, Telecommunications, Telephone, Third Interest High-Point, Time Pressure, Time Sharing,Work Schedules, Work With Work Group or Team
Organization Skills	Active Listening, Category Flexibility, Clerical, Communicating with Persons Outside, Organization, Communicating with Supervisors, Peers, or Subordinates, Coordination, Critical Thinking, Deductive Reasoning, English Language, Establishing and Maintaining Interpersonal Relationships, Getting Information, Inductive Reasoning, Information, Ordering, Monitoring, Negotiation, Oral Comprehension, Oral Expression, Organizing, Planning, and Prioritizing Work, Performing Administrative Activities, Persuasion, Problem, Sensitivity, Reading Comprehension, Service Orientation, Social Perceptiveness, Speaking, Speech Clarity, Speech Recognition, Spend Time Sitting, Time Management, Writing, Written Comprehension, Written Expression
Management Skills	Administration and Management, Coaching and Developing Others, Coordinating the Work and Activities of Others, Developing and Building Teams, Economics and Accounting, Evaluating Information to Determine Compliance with Standards, Geography, Guiding, Directing, and Motivating Subordinates, Judging the Qualities of Things, Services, or People, Management of Financial Resources, Management of Material Resources, Management of Personnel Resources, Mathematics, Monitoring and Controlling Resources, Operations Analysis, Personnel and Human Resources, Programming, Scheduling Work and Activities, Science, Speed of Closure, Staffing Organizational Units, Thinking Creatively

Physical Coordination	Arm-Hand Steadiness, Control Precision, Controlling Machines and Processes, Dynamic Strength, Exposed to Hazardous Equipment, Extent Flexibility, Gross Body Coordination, Gross Body Equilibrium, Handling and Moving Objects, Manual Dexterity, Multilimb Coordination, Performing General Physical Activities, Reaction Time, Speed of Limb Movement, Stamina, Static Strength, Trunk Strength, Wear Common Protective or Safety Equipment such as Safety Shoes, Glasses, Gloves, Hearing Protection, Hard Hats, or Life Jackets, Wrist-Finger Speed

References

[1] Youn, H. *et al.* Scaling and universality in urban economic diversification. *Journal of The Royal Society Interface* **13**, 20150937 (2016).

[2] Axtell, R. L. Zipf distribution of us firm sizes. *Science* **293**, 1818–1820 (2001).

[3] Kumar, U., Kumar, V. & KAPUR, J. N. Normalized measures of entropy. *International Journal Of General System* **12**, 55–69 (1986).

[4] Cabot, B. *et al.* Nucleotide and amino acid complexity of hepatitis c virus quasispecies in serum and liver. *Journal of virology* **74**, 805–811 (2000).

[5] Wijesekera, H. W. & Dillon, T. M. Shannon entropy as an indicator of age for turbulent overturns in the oceanic thermocline. *Journal of Geophysical Research: Oceans* **102**, 3279–3291 (1997).

[6] Eagle, N., Macy, M. & Claxton, R. Network diversity and economic development. *Science* **328**, 1029–1031 (2010).

[7] Brynjolfsson, E. & McAfee, A. *The second machine age: work, progress, and prosperity in a time of brilliant technologies* (WW Norton & Company, 2014).

[8] Carlsson, B. *Technological systems and economic performance: the case of factory automation*, vol. 5 (Springer Science & Business Media, 2012).

[9] Olsen, M. & Hemous, D. The rise of the machines: Automation, horizontal innovation and income inequality. In *2014 Meeting Papers*, 162 (Society for Economic Dynamics, 2014).

[10] Mazzucato, M. Financing innovation: creative destruction vs. destructive creation. *Industrial and Corporate Change* dtt025 (2013).

[11] Archibugi, D., Filippetti, A. & Frenz, M. Economic crisis and innovation: Is destruction prevailing over accumulation? *Research Policy* **42**, 303–314 (2013).

[12] Frey, C. B. & Osborne, M. A. The future of employment: how susceptible are jobs to computerisation? *Technological Forecasting and Social Change* (2016).

[13] Autor, D. H. Why are there still so many jobs? The history and future of workplace automation. *The Journal of Economic Perspectives* **29**, 3–30 (2015).

[14] Benessia, A. & Funtowicz, S. Sustainability and techno-science: What do we want to sustain and for whom? *International Journal of Sustainable Development* **18**, 329–348 (2015).

[15] Pajarinen, M., Rouvinen, P. & Ekeland, A. Computerization threatens one-third of Finnish and Norwegian employment. *ETLA Brief* (2015).

[16] Arntz, M., Gregory, T. & Zierahn, U. The risk of automation for jobs in OECD countries (2016).

[17] Imai, K., Keele, L. & Tingley, D. A general approach to causal mediation analysis. *Psychological methods* **15**, 309 (2010).

[18] Gerber, A. S. & Green, D. P. *Field experiments: Design, analysis, and interpretation* (WW Norton, 2012).

[19] Kok, S. & Weel, B. t. Cities, tasks, and skills. *Journal of Regional Science* **54**, 856–892 (2014).

[20] Autor, D. H., Levy, F. & Murnane, R. J. The skill content of recent technological change: An empirical exploration. Tech. Rep., National Bureau of Economic Research (2001).

[21] Autor, D. H. & Price, B. The changing task composition of the us labor market: An update of autor, levy, and murnane (2003). *Unpublished manuscript* (2013).

[22] Leitão, J. C., Miotto, J. M., Gerlach, M. & Altmann, E. G. Is this scaling nonlinear? *Royal Society Open Science* **3**, 150649 (2016).
